# Direct evidence for dynamics of cell heterogeneity in watercored apples: turgor-associated metabolic modifications and within-fruit water potential gradient unveiled by single-cell analyses

**DOI:** 10.1038/s41438-021-00603-1

**Published:** 2021-08-03

**Authors:** Hiroshi Wada, Keisuke Nakata, Hiroshi Nonami, Rosa Erra-Balsells, Miho Tatsuki, Yuto Hatakeyama, Fukuyo Tanaka

**Affiliations:** 1 Graduate School of Agriculture, Ehime University, Matsuyama, Ehime Japan; 2 The United Graduate School of Agricultural Sciences, Ehime University, Matsuyama, Ehime Japan; 3 Department of Organic Chemistry and CIHIDECAR (CONICET), University of Buenos Aires, Buenos Aires, Argentina; 4 Institute of Fruit Tree and Tea Science, National Agriculture and Food Research Organization, Tsukuba, Ibaraki, Japan; 5 Research Center for Advanced Analysis, National Agriculture and Food Research Organization, Tsukuba, Ibaraki, Japan

**Keywords:** Plant physiology, Cell biology, Plant cell biology

## Abstract

Watercore is a physiological disorder in apple (*Malus* *×* *domestica* Borkh.) fruits that appears as water-soaked tissues adjacent to the vascular core, although there is little information on what exactly occurs at cell level in the watercored apples, particularly from the viewpoint of cell water relations. By combining picolitre pressure-probe electrospray-ionization mass spectrometry (picoPPESI-MS) with freezing point osmometry and vapor pressure osmometry, changes in cell water status and metabolisms were spatially assayed in the same fruit. In the watercored fruit, total soluble solid was lower in the watercore region than the normal outer parenchyma region, but there was no spatial difference in the osmotic potentials determined with freezing point osmometry. Importantly, a disagreement between the osmotic potentials determined with two methods has been observed in the watercore region, indicating the presence of significant volatile compounds in the cellular fluids collected. In the watercored fruit, cell turgor varied across flesh, and a steeper water potential gradient has been established from the normal outer parenchyma region to the watercore region, retaining the potential to transport water to the watercore region. Site-specific analysis using picoPPESI-MS revealed that together with a reduction in turgor, remarkable metabolic modifications through fermentation have occurred at the border, inducing greater production of watercore-related volatile compounds, such as alcohols and esters, compared with other regions. Because alcohols including ethanol have low reflection coefficients, it is very likely that these molecules would have rapidly penetrated membranes to accumulate in apoplast to fill. In addition to the water potential gradient detected here, this would physically contribute to the appearance with high tissue transparency and changes in colour differences. Therefore, it is concluded that these spatial changes in cell water relations are closely associated with watercore symptoms as well as with metabolic alterations.

## Introduction

Watercored apples exhibit enhanced sweet and honey rich flavour, as they are called “Mitsu (honey)” apples in Japanese. Because of the marked sensory attributes, they have been preferred in most Asian countries and are considered to be commercially valuable in the markets; nevertheless, they are classified as a physiological disorder^1^. Watercore appears as water-soaked and translucent flesh tissues formed in the vicinity of sepal bundles in watercored apples, which generally occurs after the onset of climacteric on a tree^2^. It has been also reported that watercore is directly affected by low temperature without affecting the level of fruit maturity^3^. Therefore, elevating temperature would have an impact on the symptom formation in late (or low temperature-promoted) watercore as well as other attributes including fruit acidity and firmness, as reported previously^4^. Also, it is known that there are clear varietal differences in watercore development. For instance, ‘Fuji’ apples often exhibit watercore, whereas no or less watercore was formed in ‘Orin’ apples. Development of watercore often induces internal browning symptoms during storage, and hence ‘Fuji’ apples for long-term controlled atmosphere (CA) storage are usually harvested before being fully ripe to avoid watercore formation.

Many attempts have been made to investigate the underlying mechanism(s) of watercore in the fruits in several views^3,5–10^. In apple, it is well known that sorbitol is the primary transport carbohydrate^2,10^. It has been widely accepted that there is a close relationship between sorbitol accumulation in sink tissues and watercore symptoms^2,10,11^, and changes in membrane integrity associated with maturation and ripening have been attributed to the main cause of late watercore^2^. In contrast, the importance of sorbitol as a causal agent has been observed in early (i.e., high temperature-promoted) watercore, but not for late watercore^7^. Based on the related studies^3,6,12^, the role of sorbitol on late watercore suggested earlier has been called into the question^6^. During fruit development, phloem unloading would switch from symplasmic route to apoplastic route in apple^13^. It has been proposed that a defect in an apoplasmic active membrane transport step might induce sorbitol and sucrose accumulation, which induces water flow into the apoplastic space in parenchyma to cause late watercore^11^. Even with the many efforts, it remains obscure what exactly occurs in the affected tissue to cause the typical water-soaked (transparent) appearance. This implies the requirement of more comprehensive analysis.

Additionally, volatiles including ethanol, ethyl esters, and aldehydes are known to accumulate in the watercore region^14–16^. This illustrates that a clear shift change in glycolysis would occur in the watercore region under anoxic conditions^17^, and pyruvate would be fermented to lactate or ethanol in the cytosol. In fleshy fruits, a freezing point osmometer (e.g., Clifton osmometer) has been rarely used for determining the flesh osmotic potentials, except for few studies^18^. Most osmotic potential measurements in apple watercore have been conducted by vapor pressure methods, such as thermocouple psychrometry and a dewpoint hygrometer. Importantly, these vapor pressure methods are principally different from freezing point osmometers and capable for only determining the osmotic potential of non-volatile compounds, but not for the total osmotic potential including volatile compounds^19,20^, such as ethanol known as a major volatile in apple watercore^15,16^. Contrastingly, the freezing point osmometer allows detecting total osmotic potential of all solutes including volatile compounds. If the size of the osmotic potential of volatile compounds were significant in the watercore region^16,17^, then it is possible that the osmotic potential in the watercore region might have been underestimated. However, to the best of our knowledge, it seems likely that this possibility has never been examined in terms of watercore symptoms in any of fleshy fruits including apples. In this study, we have hypothesised that a considerable concentration of volatile compounds may exist in the water-soaked tissue in late apple watercore. Considering the cell heterogeneity of watercore symptoms, some site-specific analysis performable in the watercored tissue would be required to test this hypothesis.

A cell pressure probe has been used to measure cell water status and hydraulic properties of intact plant cells^21,22^. Combining the cell pressure probe and an Orbitrap mass spectrometer, picolitre Pressure-Probe Electrospray-Ionization Mass Spectrometry (picoPPESI-MS)^23^ technique has been successfully applied to several in situ cell-specific metabolic analyses^23–27^. In this work, the novel technique has been utilised to directly assay both the cell water status and metabolites in the following three regions: watercore region, outer region (i.e., normal cortical parenchyma) and the border (i.e., the outermost layer of watercore region) in the flesh, in both watercored and non-watercored (normal) fruit. The apparent and actual cell osmotic potentials were determined by using two methods, isopiestic psychrometry (vapor pressure method) and freezing point osmometry, respectively in order to test our hypothesis. Here, we show the presence of spatial differences in turgor pressure and the water potential gradient between the watercore region and cortical parenchyma tissue in the same fruit, demonstrating the significance of volatile compounds in the watercored tissues. From the viewpoint of cell water relations and metabolic regulation, the underlying cellular dynamics that forms a water-soaked appearance will be discussed.

## Results

### Cell water relations in each region

**Fig. 1:**
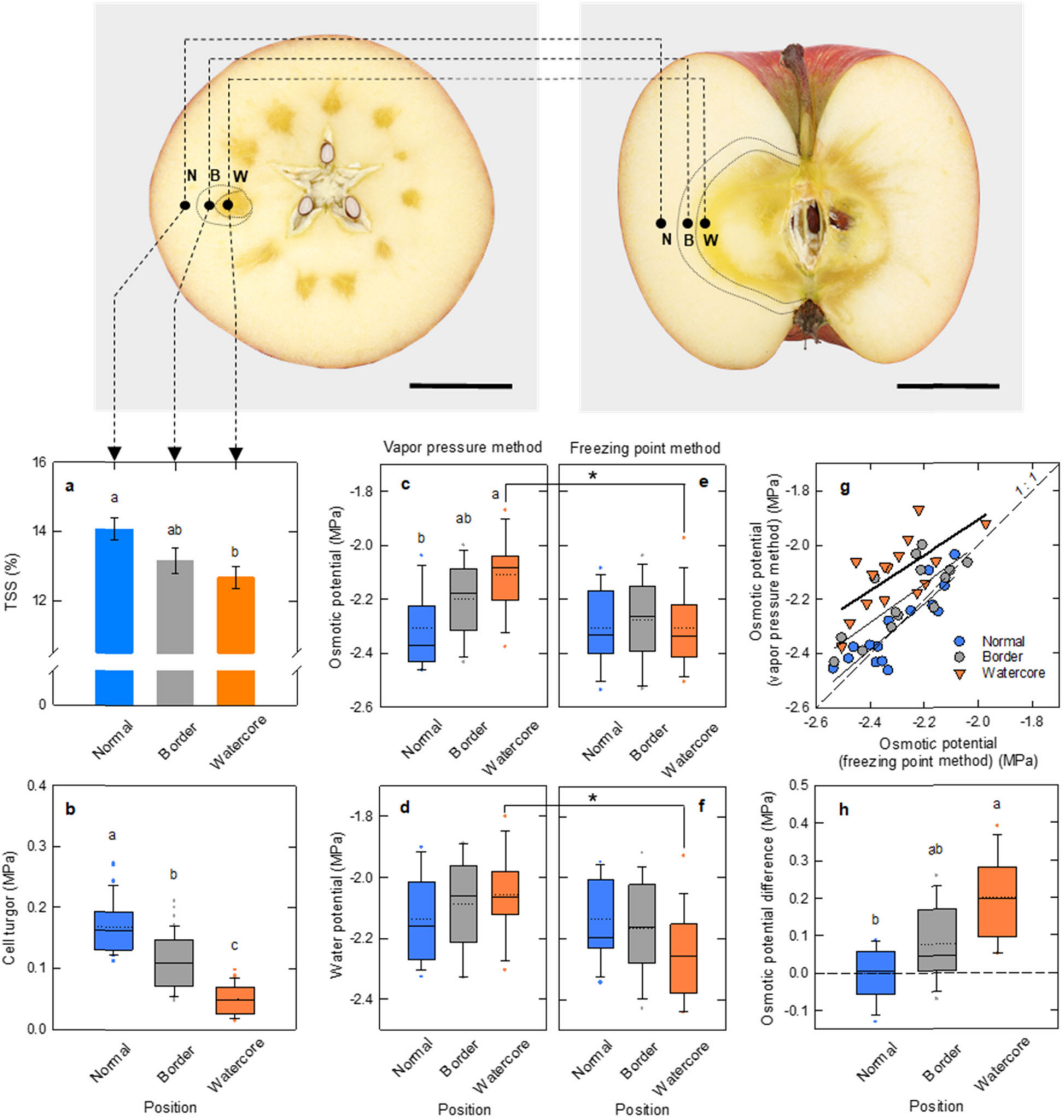
Water relations in each tissue (corresponding to normal outer parenchyma (normal), border and watercore region) in watercored apples. Total soluble solids (TSS) (**a**), cell turgor (**b**), osmotic potential determined by vapor pressure method (**c**), vapor pressure method-based calculated water potential (**d**), osmotic potential determined by freezing point method (**e**) and vapor pressure method-based calculated water potential (**f**) in each tissue in the watercored apple fruit. The osmotic potential determined by the vapor pressure method was plotted against the osmotic potential determined by the freezing point method in each tissue (**g**). The osmotic potential differences in each position were shown in **h**. The data in **a**, **c**–**f** and **h** indicate that the means ± SE of 14–15 tissues collected from 10 fruit in each treatment. The data in **b** indicate the means ± SE of 40–47 cells collected from 10 fruit in each treatment. Different letters indicate a significant difference (Tukey–Kramer test, *p* < 0.05). For watercore, asterisks are shown on the bracket across **c**–**e** and **d**–**f** indicate significance at the 0.05 probability level by *t*-test. The dashed line in **g** indicates a 1:1 line. Bars = 5 cm

The preliminary experiment comparing the water status in intact and cut fruit suggested that cell turgor of cortical cells located at 300–1500 μm below the cut surface does not change significantly for 30 min after being cut into quarters if water loss from the fruit is prevented (Fig. S1). Based on the results, cut fruit segments were provided in the following experiments (see Methods section). Total soluble solids (TSS) value was the highest in the normal outer region, followed by the border region and watercore region (Fig. 1a). When cell turgor was assayed, cell turgor in normal tissues were found to be the highest, followed by the border region and watercore regions (Fig. 1b). Spatial differences in the *apparent* osmotic potential have been detected between regions (Fig. 1c). That is, the isopiestically determined osmotic potential in normal tissues was the lowest, whereas that in watercore tissues was the highest (Fig. 1c). When calculating the water potential by subtracting the osmotic potential from the cell turgor, no spatial differences in the water potentials between the regions were observed (Fig. 1d). Contrastingly, there was essentially no difference between regions in the *actual* osmotic potential determined by the freezing point osmometer (Fig. 1e). However, a water potential gradient was found to be established between regions (Fig. 1f). When the osmotic potential determined isopiestically was plotted as a function of the osmotic potential determined by using a freezing point depression, the osmotic potential determined isopiestically was highly correlated with the osmotic potential determined with freezing point depression in normal tissues, as the line was over the equipotential line (Fig. 1g). A relatively small upward shift was observed from the equipotential line at the border, whereas the watercore regions showed a much greater shift from the equipotential line than the border, exhibiting a clear ‘osmotic gap’ (ca. 0.20 MPa) among two methods originated from the volatile compounds present in the cellular fluids (Fig. 1h).

### Spatial difference in cell metabolites

**Fig. 2:**
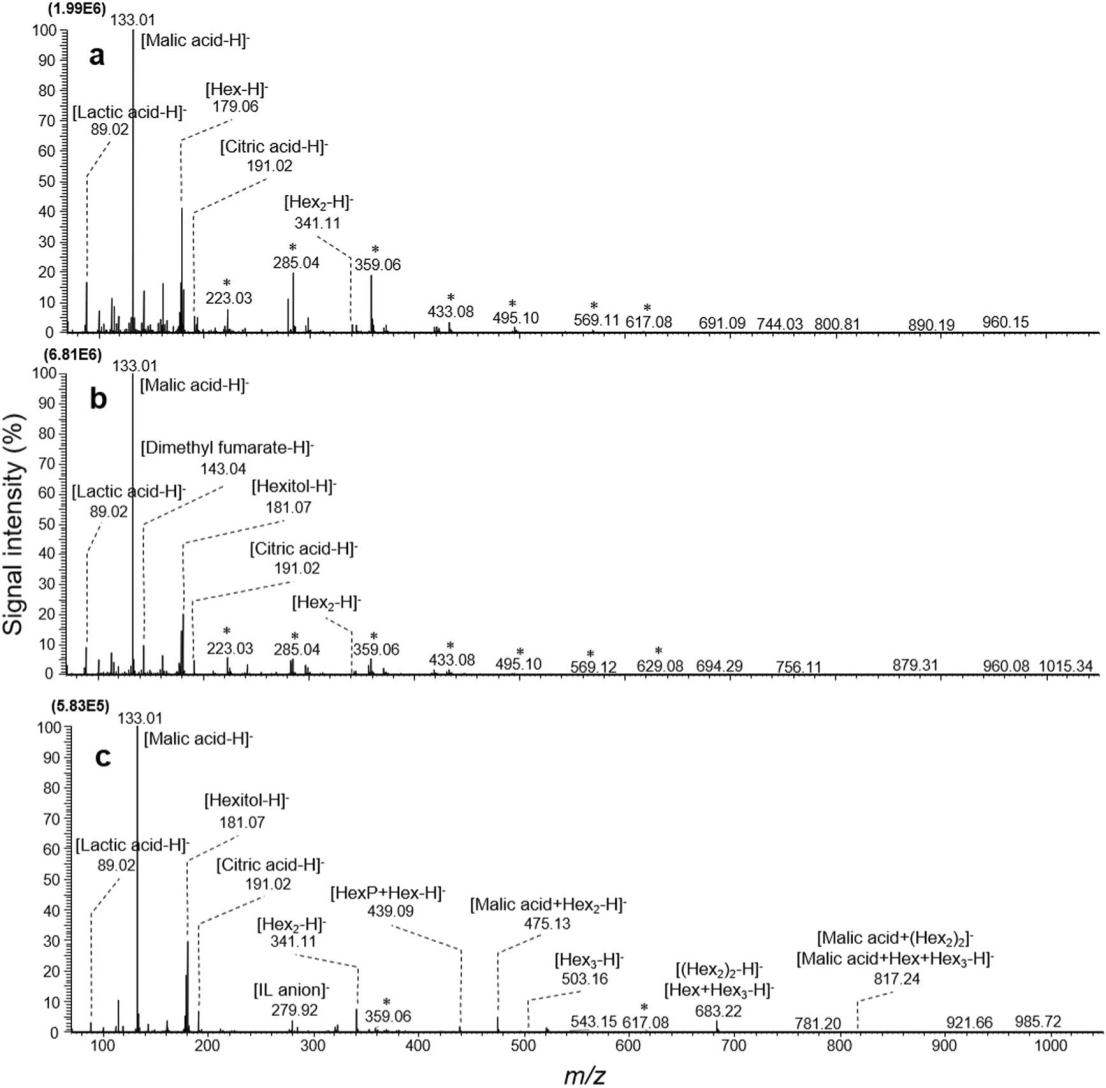
PicoPPESI negative ion mode mass spectra obtained from the cells of each region in watercored apple fruit. Data in normal outer parenchyma (**a**), border (**b**) and watercore (**c**) regions are representative of similar experiments with 10 apples in each. Asterisks indicate assigned background peaks from mixture silicone oil+ionic solution filling into the pressure probe capillary. Hex hexose, IL ionic liquid

By conducting picoPPESI-MS analysis, 102 metabolites in total (238 signals in total including cluster ions), belonging to the families of organic acids, amino acids, saccharides, volatile compounds, cell wall-related materials and phytohormones, were simultaneously detected in the watercore regions (Fig. 2 and Table S1). Metabolites were detected in negative ion mode as deprotonated [M − H]^−^ and/or [M + Cl]^−^ species, with M molecular weight as well as deprotonated [M′ − H]^−^ and/or [M′ + Cl]^−^ species, with M′ cluster weight. These clusters are the molecular aggregates stably formed in solution and in gas state because the intermolecular interaction among the molecular unities is quite strong (e.g., hydrogen bridge); its formation depends on the concentration of the molecular constituent in the analysed solution; at higher concentrations, the chance to obtain cluster signals in electrospray ionization mass spectrometry is higher. PicoPPESI mass spectrometry is an electrospray ionization mass spectrometry technique.

**Fig. 3:**
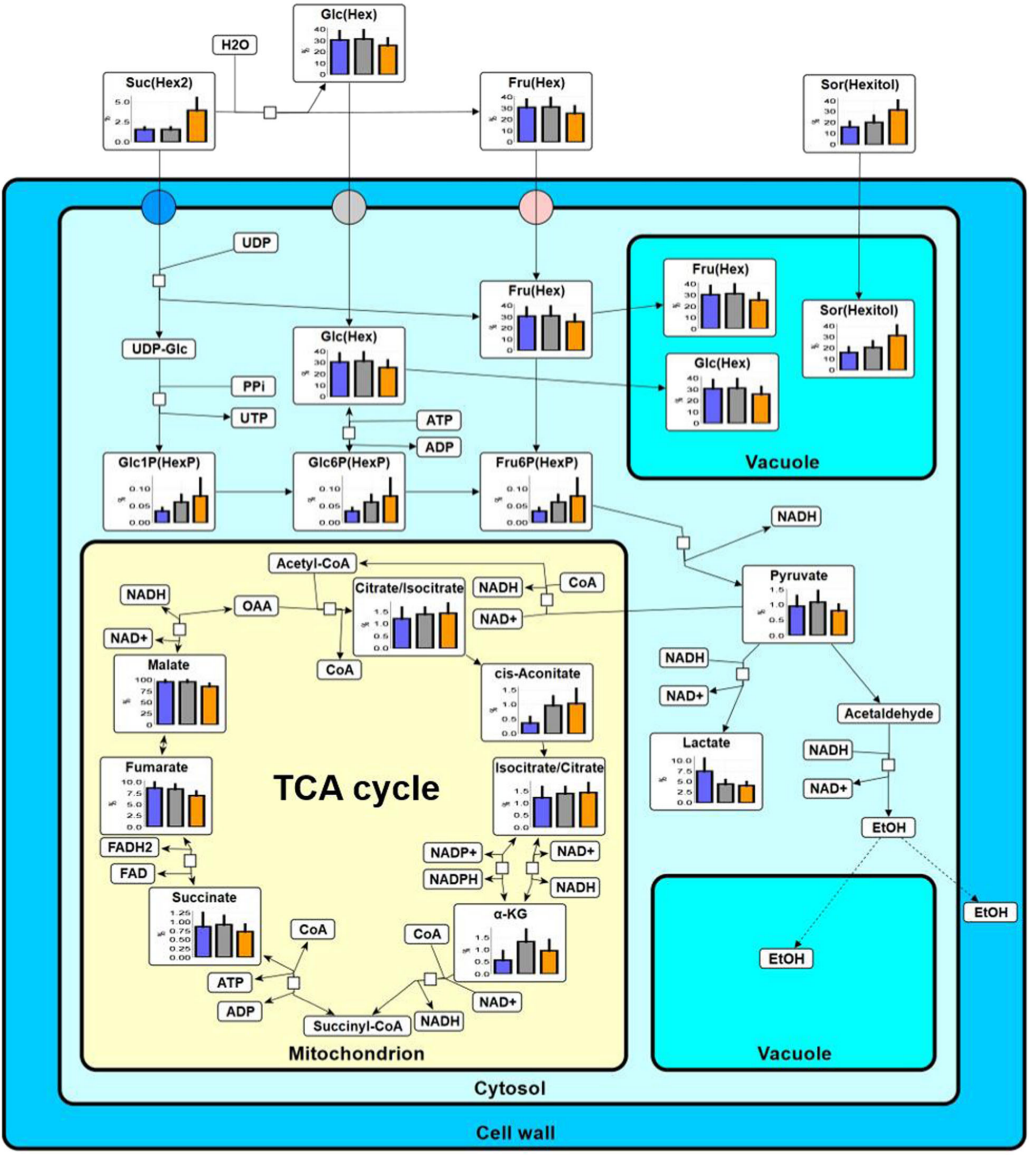
Metabolic network altered in watercored apples. In each metabolite, the horizontal line shows normal outer parenchyma (blue), border (grey) and watercore (orange) regions in the fruit. The vertical line shows the relative abundance calculated as a percentage to the base peak in picoPPESI negative ion mode mass spectra obtained from cells in each region. The data indicate the means ± SEs of 10 fruit in each region. Suc sucrose, Hex hexose, Glc glucose, Fru fructose, UDP uridine 5′-diphosphate, UTP uridine 5′-triphosphate, ATP adenosine 5′-triphosphate, ADP adenosine 5′-diphosphate, HexP hexose phosphate, Glc1P glucose 1-phosphate, Glc6P glucose 6-phosphate, Fru6P fructose 6-phosphate, NAD nicotinamide adenine dinucleotide, NADP nicotinamide adenine dinucleotide phosphate, EtOH ethanol, α-KG α-ketoglutaric acid, OAA oxaloacetic acid

**Fig. 4:**
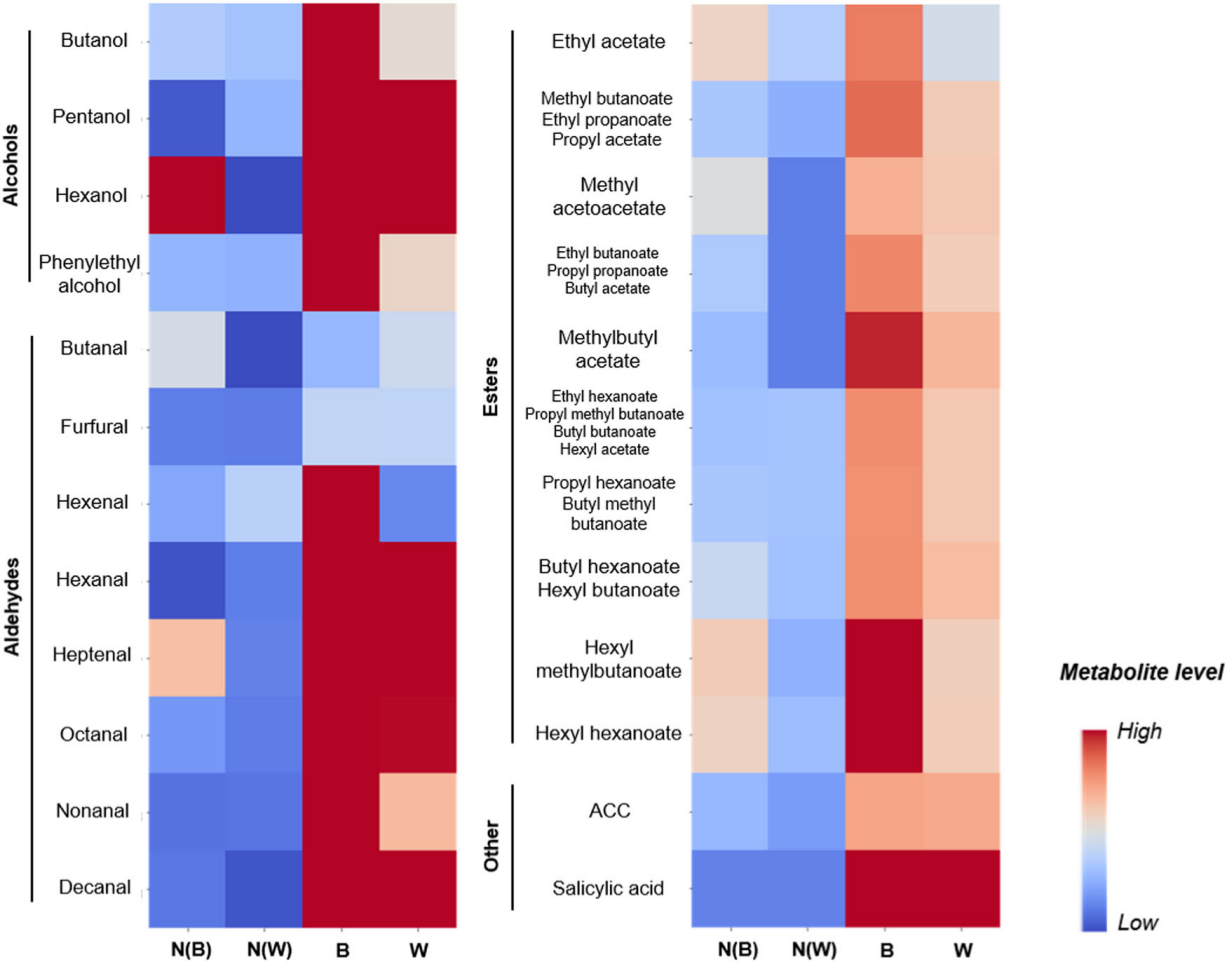
Comparison of metabolites level in the heatmap in each tissue located at the two regions corresponding to the border (N(B)) and watercore region (N(W)) in normal apple fruit and each tissue (border (B), and watercore (W) regions) in watercored apples. The metabolites level indicates the ratio of N(B) or N(W) region to normal parenchyma region of normal apple fruit, and B or W region to normal outer parenchyma region in the watercored apples. The data were the means ± SE of 7–10 apple fruit. ACC 1-aminocyclopropane-1-carboxylic acid

The peaks of metabolites related to glycolysis (e.g., Hex (*m/z* 179), Hex_2_ (*m/z* 341) and lactate (*m/z* 89)) and tricarboxylic acid (TCA) cycle (e.g., malate (*m/z* 133) and citrate (*m/z* 191)), sugar alcohol (hexitol (*m/z* 181)) and sugar-organic acid cluster ions (e.g., [Malic acid+Hex_2_-H]^−^ (*m/z* 439)) were also detected as major ions (Fig. 2). Contrastingly, the content of lactate was found to be lower in both border and watercore region, compared with the normal outer parenchyma region (Figs. 3, S2, and Table S1). Although the peaks related to both glycolysis and TCA cycle were detected in the extracted cell sap, the same trend as the decrease in lactate seen at cell level was not shown due to lack of detection sensitivity (Fig. S3). Considerable accumulation of hexitol (mostly sorbitol), saccharides (e.g., Hex_3_ (*m/z* 503)) and coumaryl-alcohol (*m/z* 149), and shikimic acid (*m/z* 173) was observed in the watercore region (Figs. 2 and 3). The concentration of wall-related metabolites was overall high in the border, followed by watercore and normal outer parenchyma region (Table S1). Besides, the peaks related to Hex_3_ were detected more frequently and strongly in the watercore region than in other regions we analysed (Figs. 2, S2, S4, and Table S2). Regarding volatile metabolites, the strong signals of alcohols (i.e., butanol (*m/z* 73), pentanol (*m/z* 87), hexanol (*m/z* 101), and phenylethyl alcohol (*m/z* 121)), aldehydes (i.e., butanal (*m/z* 71), furfural (*m/z* 95), hexenal (*m/z* 97), hexanal (*m/z* 99), heptenal (*m/z* 111), octanal (*m/z* 127), nonanal (*m/z* 141), and decanal (*m/z* 155)), and esters (i.e., ethyl acetate (*m/z* 87), methyl butanoate, ethyl propanoate, propyl acetate (*m/z* 101), methyl acetoacetate (*m/z* 115), ethyl butanoate, propyl propanoate, butyl acetate (*m/z* 115), ethyl hexanoate, propyl methyl butanoate, butyl butanoate, hexyl acetate (*m/z* 143), propyl hexanoate, butyl methyl butanoate (*m/z* 157), butyl hexanoate, hexyl butanoate (*m/z* 171) and hexyl hexanoate (*m/z* 199)) were detected in the border and watercore regions (Table S1). Ethylene precursor 1-aminocyclopropane-1-carboxylic acid (ACC) and salicylic acid were detected at both border and watercore region at high frequencies (Fig. 4 and Table S1). In order to identify hexitol, a sugar alcohol that was strongly detected in both positive and negative ion modes (Figs. 2 and S2–S5), MS/MS spectra obtained from d-Mannitol, d-Sorbitol, and the extracted cell sap were shown in Figs. S6-1, S6-2 and S6-3, respectively. The relative intensity ratio of [(M–H_2_O)-H]^−^ (*m/z* 163) signal to [M–H]^−^ (*m/z* 181) signal in Figs. S6-1, S6-2 and S6-3 was estimated to be approximately 0.34, 0.17 and 0.14, respectively. Thus, it can be considered that the peak at *m/z* 181 is composed mostly of sorbitol based on picoPPESI-MS spectra in negative ion mode.

### Differences in gene expression between normal and watercored apples

The expression levels of genes in relation to pyruvate decarboxylase (PDC), alcohol dehydrogenase (ADH), pectin methylesterase (PME) and polygalacturonase (PG) were compared in normal and watercored fruit (Fig. S7) to cross check the results of metabolome analysis on the shift change of glycolysis. There was no significant change in *MdADH1-1*, although expression levels of both *MdADH1-2* and *MdADH2-1* genes were greater in watercored fruit than that of normal fruit, suggesting the activation of fermentation, consistent with the metabolome data detected at cell level using picoPPESI-MS (Figs. 3 and S7a–d). In addition, there was no significant change in *MdPME2*, whereas *MdPG1* was dramatically down-regulated in the watercored fruit (Fig. S7e, f), suggesting that a partial modification of cell wall structure might also occur.

### Microscopic observations and colour difference in watercore regions

**Fig. 5:**
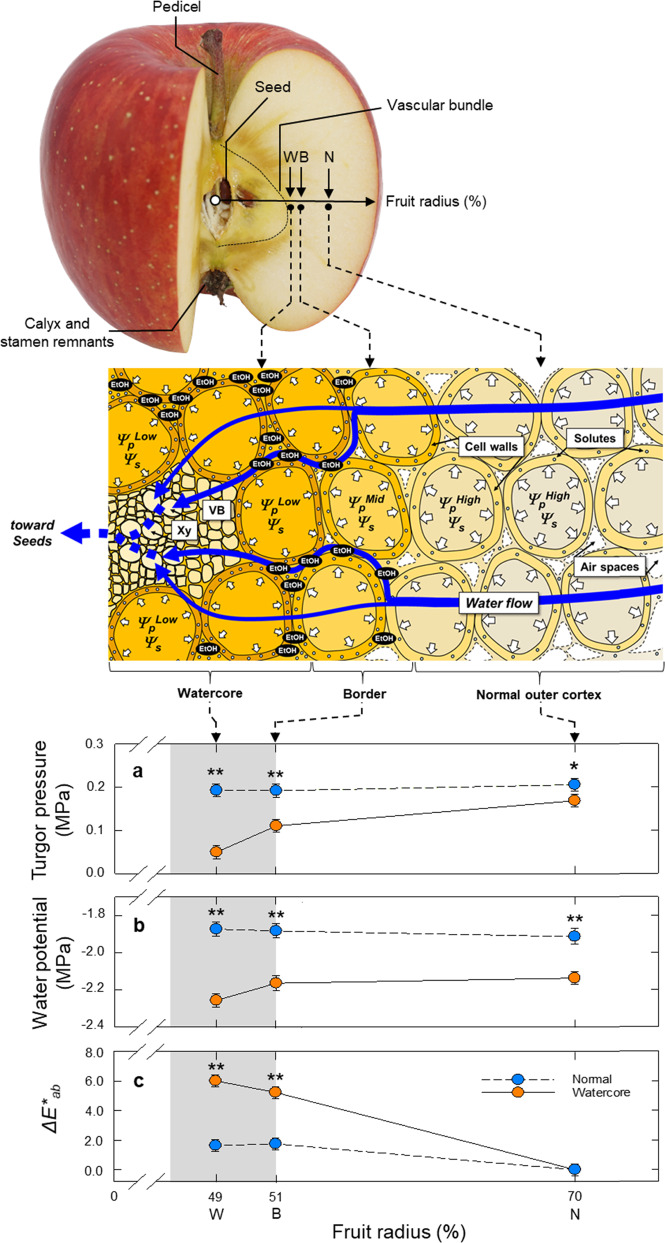
Diagram illustrating within-fruit water potential gradient associated with watercore appearance. Cell turgor in each tissue (corresponding to watercore (W), border (B) and normal outer parenchyma (N) regions) as a function of radius (%) in the normal or watercored apple fruit (**a**). Distribution of water potential determined by in situ turgor assay combined with freezing point depression method in each tissue in normal or watercored apple fruit (**b**). Colour difference (ΔE*_ab_) was calculated from the L*a*b* colour space in each position (see Methods section) (**c**). In the normal apple flesh, light reflection is randomly caused by the numerous air spaces present in apoplastic space (mostly intercellular air spaces, see the upper cartoon), and then light is scattered and does not pass through from the cut surface to deeper tissue, causing an increase in lightness value (L*). In contrast, apoplastic spaces including air spaces in watercore flesh were filled with water, alcohols, and solutes (see text), resulting in a decline in L* value contrastingly with an increase in b* value (yellowish appearance change) in the flesh. Note that in the cartoon, putative apoplastic solutes associated with turgor regulation were drawn, and the solutes contained in the cells are removed to simplify. The data were the means ± SE of 7–10 apple fruit. Significant difference at the 0.05 and 0.001 probability levels by *t*-test indicated with * and **, respectively. VB vascular bundle, Xy xylem

In the comparison of watercore regions in the watercored fruit and corresponding regions in the normal fruit, a major organelle in the cells was a central vacuole, whose volume corresponded to the range between 91.9 and 96.6% (*n* = 10) of the cells. The rest corresponds to the cytosolic compartmentation, located at the vicinity of plasma membranes and central vacuoles (Fig. S8). The transmission electron microscopy (TEM) after tissue fixation indicated that there were no obvious differences in cell anatomy regardless of the presence of watercore in the flesh (Fig. S8). In the cytosol, several organelles, such as mitochondria, peroxisome and small vacuole-like structures, were consistently observed regardless of the presence of symptoms (Fig. S8). There were no treatment differences in the number and size of the central vacuole, mitochondria, and small vacuole-like structures (Table S3). The ratio of apoplastic space per cell in watercore regions and corresponding regions in the outer parenchyma region were 26.2% and 24.9%, respectively, with no significant difference (Table S3). Colour measurements showed clear regional differences in L*, b* and ΔE*, but with relatively small changes for a*. Considerable reductions in L* with an increase in b* and ΔE* were also observed in watercore regions, corresponding to the spatial variation in cell water potentials (Figs. 5 and S9). Also, watercore development in the apple fruit used in this experiment occupied between 3.1 and 22.7% (12.1 ± 6.0% on average) per fruit cross-sectional area, and the watercore rating was 2.80 ± 1.03 (mean ± SD, *n* = 10).

## Discussion

Single-cell analyses combining picoPPESI-MS with two types of principally different osmometers unveiled that apple watercored fruits exhibited a remarkable spatial difference in cell water relations across the tissues (Fig. 1). Metabolic changes occurred dynamically between tissues in watercored fruit, compared to those of normal fruit (Fig. 3 and Table S1). These changes associated with watercore formation were tightly coordinated with changes in cell water relations. Particularly, cell metabolomics has revealed that at the border, pyruvate would have been fermented to actively produce alcohols and esters at moderately low turgor (Fig. 3), consistent with the gene expression data (Fig. S7). In contrast, high turgor was marked in the normal outer parenchyma region in fruit regardless of the presence of watercore, with no or little metabolic changes in glycolysis (Figs. 2–4). Importantly, a significant ‘osmotic gap’ has been detected between apparent osmotic potential determined with a vapor pressure method and actual osmotic potential determined by freezing point osmometry, but with no spatial variations in the actual osmotic potential across flesh. Since the vapor pressure method does not detect any volatiles^19,28^, the observed ‘osmotic gap’ could be attributed to the presence of significant concentration of volatile compounds including ethanol in the watercore region at least in ‘Fuji’ apple. Alcohols and esters generated in the cytosol under low oxygen conditions should have rapidly penetrated membranes because of the low reflection coefficients^29^, and hence it is very likely that they would penetrate plasma membranes to fill the apoplastic space. In addition to the concentration gradient of volatile compounds (i.e., osmotic gap), our single-cell analyses indicate that a large enough water potential gradient (ΔΨ_w_ = 0.16 MPa) was established between tissues in watercored fruit (Fig. 5b). These events would physically lead to high tissue transparency with changes in colour differences (Fig. 5) as a typical watercore appearance. Therefore, we conclude that site-specific changes in cell water relations and metabolisms are essential cellular events of watercored fruit, in addition to the accumulation of sorbitol and sucrose in the apoplast due to an arrest of active membrane transport previously proposed^2,11^. We also suggest that cares may be necessary when conducting the osmotic potential measurements in future studies, particularly in watercore studies.

In this work, the fruit cut in half was provided for assaying the cell water relations and metabolomics in the watercore tissues. Direct cell turgor measurements have been conducted in fleshy fruit, such as pericarp cells in tomato fruit^30^, cortical cells in apple fruit^31^ and mesocarp cells in grape berries^32–34^. Technically, cell pressure probe allows to determine the water status (turgor) of the inner cells located at up to 2.9 mm below epidermis^35^, which is to our knowledge the deepest record performed in the past pressure probe studies. There is no direct method available for assaying in much deeper cells located at >2.9 mm, such as ‘Fuji’ apple watercore region located at >25.3 mm on average below epidermis. When the excised tissues were compared with intact tissues in this study, there was a linear relation on cell turgor between intact and cut fruit segments in 30 min after being cut into quarters across a broad range (Fig. S1). It is possible that cell turgor loss might occur after cutting the fruit. However, this data ruled out the possibility if water loss from the fruit is prevented thoroughly. After conducting in situ turgor assay, cell osmotic potentials were determined by using two principally different methods, the vapor pressure method and freezing point osmometry in the same samples. If the concentration of volatile compounds in the fluids has an impact to total osmotic potential, a disagreement should be observed between methods. In this study, we tested this to detect considerable ‘osmotic gap’ at both watercore region and border (Fig. 1c, e). The fundamental assumption for the vapor pressure osmometer is that only water molecule is present in the vapor phase at equilibrium. Therefore, any volatile compounds including ethanol cannot be detected by the vapor pressure osmometry (as well as the isopiestic psychrometers used here)^19,28^, which is different from the freezing point osmometry.

In apple, this assumption was confirmed by providing a series of model apple juices that contained four different ethanol concentrations, 50, 100, 150, and 200 mM as final concentration, taking into account that ethanol is a predominant volatile in apple watercore^15^. The samples from cortical parenchymal cell sap in watercored fruit (see ‘N’ in the photograph in Fig. 1) were pooled and centrifuged. And then, the isopiestic psychrometer as vapor pressure method and nanolitre osmometer as freezing point osmometry were used to determine each of the osmotic potentials. The results indicate that the osmotic potential determined isopiestically (vapor pressure method) was essentially similar over the range regardless of the addition of ethanol. In contrast, it has been demonstrated that the osmotic potential determined by freezing point osmometry was highly correlated with the molarity of ethanol at least up to 200 mM (Fig. S11). In addition, the regression line for freezing point osmometry was within the confidential bands, matching with the theoretical curves obtained from Wolf et al.^20^ (see Fig. S11).

More recently, it has been reported that numerous volatile compounds are involved in the watercore apples^1^, and thus the discrepancy observed as the shift of regression lines (Fig. 1g) could be attributed to the presence of the volatile compounds in the cellular fluids collected. In contrast to the watercore regions, a positive correlation along with the equipotential line was observed in the corresponding zone in the normal fruit (see Fig. S10g). Based on the spatial differences in cell metabolites (Figs. 2–4, Tables S1 and S2), none of the normal tissues in watercored fruit and non-watercored fruit are likely to actively induce fermentation, consistent with other studies^15,17^. Although psychrometers have been long used to measure the water status in apple fruit^6,36,37^, these osmotic potential measurements conducted in watercore regions might be underestimated.

Our microscopic observations using transmission electron microscopy (TEM) confirmed that the majority space of the cells in watercore regions was filled with the central vacuole in the cells and the volume of cytosol was relatively small in the cells, and there were no obvious morphological differences as well as the ratio of apoplastic space itself at least at the stage examined (Fig. S8). Because TEM analysis requires tissue fixation at preparation, it appears that this fixation process led to no clear anatomical differences. The low porosity exhibiting in watercore regions^16,38^ leads to the appearance with high transparency and large colour differences as observed (Figs. 5c and S9).

In general, total water potential (Ψ_w_) of cells in plant tissues is described as

1 $$\psi _{\mathrm{w}} = \psi _{\mathrm{s}} + \psi _{\mathrm{p}}$$

where Ψ_p_ represents cell turgor, and the Ψ_s_ is equivalent to Ψ_s_^pro^ (i.e., protoplast osmotic potential determined by freezing point osmometer in this study). By recognising that apple fruit cells form two compartments separated by plasma membranes, the water potential of the protoplast (Ψ_w_^pro^) and apoplast (Ψ_w_^apo^) can be described separately as:

2 $$\psi _{\mathrm{w}}^{{\mathrm{pro}}} = \psi _{\mathrm{s}}^{{\mathrm{pro}}} + \psi _{\mathrm{p}}^{{\mathrm{pro}}}$$

3 $$\psi _{\mathrm{w}}^{{\mathrm{apo}}} = \psi _{\mathrm{s}}^{{\mathrm{apo}}} + \psi _{\mathrm{m}}^{{\mathrm{apo}}}$$

where Ψ_s_^apo^ and Ψ_m_^apo^ are regarded as the apoplastic osmotic potential and matric potential, respectively. Protoplast water potential can be equilibrated with the apoplastic water potential, i.e. Ψ_w_^pro^ ≈ Ψ_w_^apo^, as described previously^33,39^. In this study, Ψ_p_^pro^ and Ψ_s_^pro^ were directly determined, but not for Ψ_s_^apo^ and Ψ_m_^apo^. In grape berry development, Ψ_m_^apo^ gradually increased until the onset of ripening (i.e., veraison) to remain almost zero during the ripening^34^, whereas significant concentration of apoplastic solutes started to accumulate prior to veraison to reduce Ψ_s_^apo^, causing a loss of turgor correlated with fruit softening^33,34^. Likewise in apples, it has been reported that fruit softening would be caused by a reduction in turgor^31,40,41^, implying a potential role of solutes accumulated in apoplast that corresponds to a relatively small fraction^42^. This suggestion is consistent with the switching to apoplastic phloem transport during ripening in apples^13^. To date, there seems to be no similar studies on cell water relations during fruit development; however, turgor loss observed at both border and watercore region would be explained by the presence of higher concentration of apoplastic solutes, compared with the outer parenchyma region in watercored fruit and any regions in normal fruit (see illustration in Fig. 5). It is noteworthy that watercore regions exhibited the lowest turgor at least in the three regions assayed here (Fig. 1b). Considering the potential role of turgor on nuclear gene expression as a pressure pulse^43–46^ and the proposed metabolic network (Fig. 3) together, it is possible to speculate that changes in turgor might be associated with upregulation of the gene expression of alcohol dehydrogenases in the cells prior to the watercore formation on a tree. In this experiment, we conducted only a snapshot analysis in the detached fruit. It remains questionable whether turgor loss precedes to a series of metabolic changes in relation to watercore formation. Conducting time-course of site-specific analysis will be required to answer the question.

The reflection coefficient (*σ*) is a dimensionless quantity that indicates the percentage of solute that prevented from penetrating the membrane^22^. The values of less lipophilic solutes including ethanol are typically smaller than those of lipophilic solutes^22^. It has been known that ethanol is highly permeable to the membranes with a small reflection coefficient (*σ* = 0.21) in *Tradescantia virginiana* epidermal cells^29^, and hence the osmotic effect of ethanol over membranes is transient. In this study, ethanol signal was undetectable because of the technical limitation in Orbitrap MS we used (out of detectable *m/z* range); however, based on the previous reports^1,47^ and present study, it has been suggested that the *σ* of volatile compounds including alcohols and esters present in watercored apples would be either similar to or lower than ethanol, presumably much more smaller than that of sucrose (*σ* = 1.10)^29^. Taken together, it is quite reasonably assumed that alcohols including ethanol and esters generated in cytosol would rapidly penetrate membranes to accumulate in the apoplast, partially contributing to a slight volume increase in vacuole-like structures in the cytosol (Figs. 3, 4 and S8, and Table S3). One might expect that the degradation of cell wall components by PME may be associated with an increase in alcohol concentration, and an increase in the enzyme activity may accelerate the watercore formation, as suggested previously^1,17^. However, this may not be the case based on the gene expression data in this study (see *MdPME2* in Fig. S7e). This possibility remains to be investigated further. Interestingly, our single-cell analyses revealed that the content of major volatile metabolites (odours)^48^, such as alcohols and esters, was overall greater in the border rather than the inner watercored region (Fig. 4). It is anticipated that alcohols (mostly ethanol) produced in the cytosol of the watercore region may quickly penetrate the membranes to accumulate at the outermost layer of the watercore region, where intense signals of various volatiles have been detected (see Fig. 4 and Table S1). Also, it is possible that some alcohols might be immediately condensed with fatty acyl-CoAs in mitochondria^49^. Although the fate of volatiles (see Fig. 4) including ethanol originated from the cytosol was not determined in this study, it is reasonably speculated that most volatiles may be pneumatically diffused through air spaces in the surrounding cortical parenchyma toward epidermis, where retains high porosity^42,50^ (see Fig. 5). This would result in evolution of the typical rich flavour in watercored apples.

In normal fleshy fruits, it has been generally recognised that mesocarp turgor is spatially maintained (e.g., grape fruits^32^). In this study with the detached fruits, adopting single-cell analyses allowed us to detect remarkable turgor and water potential gradients across flesh in apple watercored fruit (Fig. 5a, b). It should not be ignored that watercored fruits exhibit the water-soaked symptom in the vicinity of the vascular core, filling up with water that presumably contained with ethanol and other volatiles, which leads to a considerable reduction in the gas phase in air spaces^16^ to reduce the porosity^50^ (see Fig. 5). This would also imply no or negligible matric potential existing in watercore regions, consistent with the above assumption. Similar to the previous studies^16^, our metabolome data support that watercore regions would be under low oxygen and high carbon dioxide concentrations (Figs. S2 and S4).

In addition, it should be noted that there was a remarkable water potential difference established between the watercore region and developing seeds, regardless of the presence of watercore symptoms in the fruit (Fig. S12). In watercored fruit, seed water potential determined isopiestically was found to be lower than that of normal fruit (Figs. S12). These data strongly suggest that watercored fruit established a fairly steep water potential gradient from normal outer cortical parenchyma region to seeds, passing through the watercore region at least in detached ‘Fuji’ apple (Figs. 5 and S13). Because hydration of the embryo is a prerequisite for dormancy removal in apple^51^, one can expect that the water potential gradient detected here may be associated with embryo development. Although this awaits future study, the observed within-fruit water potential disequilibrium as well as turgor change across flesh may be closely associated with the altered metabolic regulations simultaneously observed in the watercore region using picoPPESI-MS.

Currently, targeted mutagenesis technologies, such as genome editing, have been widely extended in most crops including apples^52^. Although further studies will be required in terms of varietal differences in watercore formation in the view of both genetics and cell-omics, the findings of this study also imply that both formation and suppression of watercore may be genetically manipulatable with the technologies, such as an exogenous small interfering ribonucleic acid (siRNA), besides low temperature. This may be a promising approach to contribute to stable apple production under elevated temperature conditions.

## Materials and methods

### Plant materials

Apple (*Malus* *×* *domestica* Borkh. cv. Fuji) fruits were grown in the orchard in Kurokawa, Morioka, Iwate, Japan (39° 63′ 30″ N, 141° 19′ 73″ E, altitude 188 m), and the watercore and normal fruit were both harvested on 11 November 2017, 16 November 2018 and 14 November 2020. The fruit was then temporarily stored in the storage facility located at ca. 1 km away from the orchard, and then transported to Ehime University on 15 November 2017, 20 November 2018 and 17 November 2020, and stored at the refrigerator (5 °C, 70% relative humidity (RH)) until the experiments. Prior to the measurements, the fruit was covered with a plastic bag and transferred to the room set at 20 °C and 70% RH to be equilibrated at the room for 20 min prior to the experiment. Additional ‘Fuji’ apple fruit were obtained commercially and transported to the laboratory to be used for a preliminary experiment on cell turgor measurement. Following the method of the previous study^3^, watercore development in each fruit was assessed by visual rating (1 = trace, 2 = light, 3 = moderate and 4 = severe).

### In situ turgor assay followed by the determination of cell metabolites

After the fresh weight of each fruit was measured, the fruit was moved to the water-saturated glove box^22^. In the glove box, the fruit was cut in half to photograph the pattern of watercore and immediately cut into quarters, and then the cut surface was gently coated with Vaseline and covered with cling film, so that the water loss from the fruit could be minimised during the experiment^32^. The fresh weight of the quarter sample was measured. The sample segment was gently fixed on the sample holder, and then the cell pressure probe technique^53^ was used to measure in situ cell turgor of cortical cells located between 420 and 1180 μm below the cut surface in watercore (located at 5 mm inside from the border), border (corresponding to the outermost layer edge of watercore appeared on the cut surface), and normal outer parenchyma region (non-watercore, located at 5 mm outside from the border of watercore) regions on the same sample were sequentially analysed (Fig. 5).

Additionally, several types of ‘Fuji’ apples, normal fruit harvested in early November 2017, watercored fruit harvested in early November 2017, and cold-storage fruit in late February 2018, were used to determine cell turgor changes after cutting, as described above. According to the previous work^31^, cell turgor of the several parenchyma cells, ranging between 300 and 1500 μm below epidermis in intact fruit, were assayed in situ (see inset in Fig. S1). And thereafter, the fruits were cut into quarters under humid conditions, and then the newly cut ¼ segment was fixed on the sample holder, so that the probe tip could be perpendicularly inserted into the corresponding region of the same fruit through the cut surface (see illustration in Fig. S1). And immediately, the same depth of cells in the adjacent region to where already penetrated were used to determine turgor (Fig. S1).

After in situ turgor determination, cell metabolome analysis was carried out by using picolitre Pressure-Probe Electro Spray-Ionization Mass Spectrometry (picoPPESI-MS)^23^ in each region. Cell sap was collected by depressurising inside the microcapillary, and the tip immediately oriented toward the orifice of an Orbitrap mass spectrometer (Orbitrap Elite, Thermo Fisher Scientific Inc., MA, the US) was electrified with −4 kV using a high voltage generator (AKTB-05k1PN/S, Touwa Keisoku Corp., Tokyo, Japan). The full scan spectra were acquired with the instrumental settings of 200 ms as maximum injection time, inlet ion transfer tube temperature of 275 °C, resolution of 60,000 and an automatic gain control (AGC) value of 1 × 10^6^. When the target cells were successfully impaled to collect picolitre cell sap without tip plugging, the entire process of picoPPESI-MS analysis on the cells in negative ion mode was completed within few minutes. Besides, extracted cell sap from each region after the in situ analysis (see the section below for extraction method) were individually measured in positive/negative ion modes and the results were double-checked. All the standard chemicals and organic solvents used in the experiments were liquid chromatography-mass spectrometry (LC/MS) grade and purchased from Wako Pure Chemical Industries, Ltd. (Osaka, Japan). For picoPPESI-MS operation, an ionic liquid, trihexyl (tetradecyl) phosphonium bis trifluoromethanesulfonyl amide (Cyphos IL109 Strem Chemicals Inc., MA, the US) was suspended in phenyl methyl silicone oil (Wacker silicone fluid AS4, Munich, Germany) at a concentration of 0.01% (v/v) to enhance electric conductivity of the silicone oil^23^. All manipulations were conducted under a digital microscope (KH-8700, HIROX Co. Ltd., Tokyo, Japan), and the sample attached to the sample holder was humidified during all processes. All the analysis in the same sample was completed in 30 min, at which no obvious colour change (browning) was observed on the cut surface.

Exact monoisotopic *m/z* values for all the peaks on the mass spectra acquired were extracted using the Qual Browser application in the Thermo Xcalibur software (ThermoFisher Scientific). Metabolites were identified from the theoretical masses of candidate metabolites in the METLIN online metabolomics database (http://metlin.scripps.edu/index.php), allowing differences of <5 ppm, and the limit of detection (LOD) was determined by the signal intensity of the substance reaches at least three times the signal noise of the baseline. In addition to the in situ analyses described above, MS/MS analysis for hexitol was conducted on d-Mannitol, d-Sorbitol and the extracted cell sap. Collision-induced dissociation (CID) tandem MS analysis in negative ion mode was performed using the same Orbitrap MS coupled with the picoPPESI system. The MS/MS scan spectra were acquired with the instrumental settings of 200 ms as maximum injection time, inlet ion transfer tube temperature of 275 °C, resolution of 240,000 and an AGC value of 5 × 10^4^. We have built a metabolic network with Visualization and Analysis of Networks conTaining Experimental Data (VANTED) v2.7.2 (https://www.cls.uni-konstanz.de/software/vanted/) and created a heatmap with Python 3.9.1 using Matplotlib and Seaborn.

### Osmotic potential determination of the cellular fluids

After the above in situ analysis, tissue segments from each region were gently extracted using a 4.0-mm cork borer along the direction of tip insertion of the cell pressure probe, and 5-mm height of tissue segment was cut out and collected in the sample tube to store −80 °C for >2 h. After thawing the segment, all the samples were centrifuged at 1500×*g* for 20 min at 4 °C, and extracted sap was collected from the bottom of the centrifuge tube^33^. An aliquot of the extracted fluid was then quickly transferred into high viscous microscopic oil placed onto a diamond plate^54^ and the osmotic potentials of cellular fluids to determine using both nanolitre freezing point osmometer (Clifton Technical Physics, Hartford, NY)^21,54^, and isopiestic psychrometry^22,55^. The content of total soluble solids (TSS) was determined by using a refractometer. In some cases, cortical parenchyma cell sap from watercored fruit used above were pooled. After centrifugation, an aliquot of the supernatant and ethanol were mixed to produce a series of model apple juice samples that have four different concentrations, 50, 100, 150 and 200 mM as the final concentration. The isopiestic psychrometer (vapor pressure method) and nanolitre osmometer (freezing point osmometry) were used to determine each osmotic potential of the samples.

### Gene expression analysis in normal and watercored tissues

Normal and watercored apples in the 2018 experiment (see Plant materials) were horizontally cut into 1 cm thickness at the equator of each fruit. In four positions, the normal outer parenchyma region and watercore region in watercored fruit, as well as the corresponding outer parenchyma region and non-watercore region in normal fruit, an 8-mm diameter cork borer was used to collect each tissue disk. The disk samples were frozen in liquid N_2_ and maintained at −80 °C for RNA extraction. Target genes were the genes encoding enzymes related to ethanol synthesis (PDC, ADH) and cell-wall degradation (PME, PG). Real-time quantitative reverse transcription-polymerase chain reaction (qRT-PCR) was carried out to assess expression levels of the relevant six genes in total. According to the previous studies^17,56^, total RNA was extracted from the frozen samples by using the hot borate method^57^ treated with DNase I (TURBO DNA-free Kit, Ambion, Austin, CA, USA) to minimise DNA contamination. The first-strand complementary DNA (cDNA) was prepared with a High-Capacity cDNA Reverse Transcription Kit (Applied Biosystems, Foster City, CA, USA). qRT-PCR was performed in a 7300 Real-Time PCR System (Applied Biosystems) with a SYBR Premix Ex Taq kit (TaKaRa, Kyoto, Japan) and a set of primers designed using Primer Express software (Applied Biosystems) for each of six amplified genes^17^.

### Microscopy

Watercored fruit was cut in half, and segments in each position were extracted by using a 4.0-mm diameter of cork borer. Preliminary experiments were conducted to determine osmotic potential in each region. All transverse segments (1–2 mm in thickness) isolated from each region were fixed with 4% (w/v) paraformaldehyde and 270 mM (watercore region in watercored fruit), 327 mM (normal outer parenchyma region in watercored fruit) and 300 mM (normal fruit) sucrose in 100 mM sodium phosphate (pH 7.2) for 3 h at room temperature, to be similar to the observed tissue osmotic potential, typically within ±0.15 MPa. Thereafter, tissues were washed in 100 mM phosphate buffer (pH 7.2). Fixed tissues were dehydrated through an ethanol series and embedded in LR White resin (London Resin, Hampshire, the UK) by two-days polymerising at 60 °C. Semi-thin sections (appropriately 900 nm) for light microscopy were viewed, and ultra-thin sections (appropriately 80–100 nm) for electron microscopy were observed with a transmission electron microscope (TEM; JEM-1010, JEOL Ltd., Tokyo, Japan). For the organelle image analysis, the outline of mitochondria, vacuoles and small vacuole-like structures in the cells in normal outer parenchyma and watercore regions, and the cells on the light microscopic images were traced by using ImageJ software (US National Institutes of Health, Bethesda, MD, the US) to determine the area. The spatial ratio of apoplastic space and small vacuole-like structures per cell was determined. The number per cell of mitochondria in normal outer parenchyma and watercore regions was counted.

### Colour measurements

For the watercore image analysis, a colour digital camera (SO-04H, Sony Corp., Tokyo, Japan) was located vertically over the background at a distance of 30 cm. The angle between the camera lens and lighting source (FLR 40 S W/M/36, Toshiba Corp., Tokyo, Japan) axis was set to be at 40°. The spectral energy distribution of the lighting source is shown in Fig. S14. The camera settings for this experiment were manual mode with the lens aperture at *f* = 2.0, shutter speed = 1/32 s, no zoom, 3840 × 2160 pixels and storage in JPEG format. The images were normalised by using OpenCV-Python to align the variation in brightness of the backgrounds from shot to shot. *RGB* colour space in the JPEG images of each fruit was acquired with ImageJ (U.S. National Institutes of Health, Bethesda, MD, USA), and the *RGB* colour space was converted into International Commission on Illumination (CIE) L*a*b* colour space. All the algorithms for processing the colour space conversion were written in Python 3.9.1.

### Statistical analysis

Analysis of variance with Tukey–Kramer test or Student’s *t*-test was conducted for testing the significance of groups in water status measurement, metabolic analysis, microscopy, and colour measurements. The difference in gene expression was analysed using Wilcoxon rank-sum test to evaluate the difference in the representative values between the two data obtained. All statistical analyses were written and conducted in R (version 4.0.2. SAS Institute Inc., Cary, NC).

## Supplementary information

Supplementary Data

## Data Availability

All data needed to evaluate the conclusions in the paper are present in the paper and/or the Supplementary Materials. Additional data related to this paper may be requested from the authors.
