# Supplementary material for: Direct evidence for dynamics of cell heterogeneity in watercored apples: turgor-associated metabolic modifications and within-fruit water potential gradient unveiled by single-cell analyses
Source: Hortic Res. 2021 Aug 3;8:187. doi: 10.1038/s41438-021-00603-1 (PMC8333330; doi:10.1038/s41438-021-00603-1)
Supplement: Supplementary file 1 — Supplementary Data [file 41438_2021_603_MOESM1_ESM.docx]

Supplementary Information

Title: Direct evidence for dynamics of cell heterogeneity in watercored apples: Turgor-associated metabolic modifications and within-fruit water potential gradient unveiled by single-cell analyses

**Authors:** Hiroshi Wada^1,2,^*^,†^, Keisuke Nakata^2,†^, Hiroshi Nonami^1^, Rosa Erra-Balsells^3^, Miho Tatsuki^4^, Yuto Hatakeyama^1^, Fukuyo Tanaka^5,^*

**Affiliations**

^1^Graduate School of Agriculture, Ehime University, Matsuyama, Ehime, Japan

^2^The United Graduate School of Agricultural Sciences, Ehime University, Matsuyama, Ehime, Japan

^3^Department of Organic Chemistry and CIHIDECAR (CONICET), University of Buenos Aires, Buenos Aires, Argentina

^4^Institute of Fruit Tree and Tea Science, National Agriculture and Food Research Organization, Tsukuba, Ibaraki, Japan

^5^Research Center for Advanced Analysis, National Agriculture and Food Research Organization, Tsukuba, Ibaraki, Japan

^†^These authors contributed equally to this manuscript.

*Corresponding authors: Hiroshi Wada (ORCID ID 0000-0003-0510-5744; Phone +81-89-946-9824; FAX +81-89-946-9867; Email: [hwada@agr.ehime-u.ac.jp](mailto:hwada@agr.ehime-u.ac.jp)) and Fukuyo Tanaka (ORCID ID 0000-0002-0904-3248; Phone +81-29-838-7351; FAX +81-29-838-7352; Email: [fukuyot@affrc.go.jp](mailto:fukuyot@affrc.go.jp))

**Table S1. Metabolites detected in each of mesocarp cells (normal outer parenchyma (N), border (B), and watercore (W)) in the watercore apple fruit using picoPPESI-MS in negative ion mode.**

| **Metabolites detected in each of mesocarp cells (normal outer parenchyma (N), border (B), and watercore (W)) in the watercore apple fruit using picoPPESI-MS in negative ion mode.** | | | | | | | | | | | | | | | | | | | | | | | | | |
| --- | --- | --- | --- | --- | --- | --- | --- | --- | --- | --- | --- | --- | --- | --- | --- | --- | --- | --- | --- | --- | --- | --- | --- | --- | --- |
| Metabolites | | Molecular  formula | | Ion type detected  [M=molecule]  [M’=cluster] ^&^ | | Theoretical*  *m/z* | | Relative abundance (%)† | | | | | | | | | | | | | | *P* value | Frequency of detection‡ | | |
|  |  |  |  |  |  |  |  | N | | | B | | | W | | | B / N | | | W / N | |  | N | B | W |
| **Metabolites in glycolysis / fermentation** | | | |  | |  | | |  | | | |  | |  | | |  | | |  | |  |  |  |
| Pyruvic acid | | C3H4O3 | | [M-H]^-^ | | 87.0088 | | 0.945 | | | 1.078 | | | 0.803 | | | 1.14 | | | 0.85 | | 0.810 | ++++ | ++++ | ++++ |
| Lactic acid | | C3H6O3 | | [M-H]^-^ | | 89.0244 | | 7.512 | | | 4.414 | | | 4.004 | | | 0.59 | | | 0.53 | | 0.354 | ++++ | ++++ | ++++ |
| Hex | | C6H12O6 | | [M-H]^-^ | | 179.0561 | | 30.494 | | | 31.226 | | | 25.500 | | | 1.02 | | | 0.84 | | 0.823 | ++++ | ++++ | ++++ |
| Hex | | C6H12O6 | | [M+Cl]^-^ | | 215.0328 | | 3.149 | | | 1.328 | | | 0.470 | | | 0.42 | | | 0.15 | | 0.362 | ++++ | ++++ | ++++ |
| HexP | | C6H13O9P | | [M-H]^-^ | | 259.0224 | | 0.035 | | | 0.061 | | | 0.079 | | | 1.75 | | | 2.27 | | 0.617 | +++ | +++ | +++ |
| Hex_2_ | | C12H22O11 | | [M-H]^-^ | | 341.1089 | | 1.574 | | | 1.533 | | | 3.906 | | | 0.97 | | | 2.48 | | 0.126 | ++++ | ++++ | ++++ |
| Hex_2_ | | C12H22O11 | | [M+Cl]^-^ | | 377.0856 | | 0.756 | | | 0.193 | | | 0.189 | | | 0.26 | | | 0.25 | | 0.139 | +++ | +++ | ++++ |
| UDP-Hex | | C15H24N2O17P2 | | [M-H]^-^ | | 565.0477 | | 0.000 | | | 0.005 | | | 0.001 | | | - | | | - | | 0.204 | - | + | + |
|  | |  | |  | |  | |  | | |  | | |  | | |  | | |  | |  |  |  |  |
| Phosphorous acid | | H3O3P | | [M-H]^-^ | | 78.9591 | | 0.015 | | | 0.051 | | | 0.041 | | | 3.50 | | | 2.80 | | 0.260 | ++ | +++ | +++ |
| Phosphoric acid | | H3O4P | | [M-H]^-^ | | 96.9696 | | 0.492 | | | 0.839 | | | 0.828 | | | 1.71 | | | 1.68 | | 0.616 | ++++ | ++++ | ++++ |
| Uridine 5'-diphosphate (UDP) | | C9H14N2O12P2 | | [M-H]^-^ | | 402.9949 | | 0.018 | | | 0.055 | | | 0.051 | | | 3.07 | | | 2.85 | | 0.093 | ++ | +++ | +++ |
|  | |  | |  | |  | |  | | |  | | |  | | |  | | |  | |  |  |  |  |
| **Cluster ions** | |  | |  | |  | |  | | |  | | |  | | |  | | |  | |  |  |  |  |
| Pyruvic acid+Hex | | C9H10O9 | | [M’-H]^-^ | | 267.0722 | | 0.393 | | | 0.187 | | | 0.105 | | | 0.48 | | | 0.27 | | 0.236 | ++++ | ++++ | ++++ |
| Pyruvic acid+Hex_2_ | | C15H26O14 | | [M’-H]^-^ | | 429.1250 | | 0.030 | | | 0.013 | | | 0.011 | | | 0.44 | | | 0.38 | | 0.135 | ++ | +++ | ++ |
| Pyruvic acid+Hex_2_ | | C15H26O14 | | [M’+Cl]^-^ | | 465.1017 | | 0.245 | | | 0.647 | | | 0.464 | | | 2.64 | | | 1.89 | | 0.169 | ++++ | ++++ | ++++ |
| Lactic acid+Hex | | C9H18O9 | | [M’-H]^-^ | | 269.0878 | | 5.342 | | | 0.749 | | | 0.474 | | | 0.14 | | | 0.09 | | 0.150 | ++++ | ++++ | ++++ |
| Lactic acid+Hex_2_ | | C15H28O14 | | [M’-H]^-^ | | 431.1406 | | 0.247 | | | 0.016 | | | 0.039 | | | 0.07 | | | 0.16 | | 0.348 | +++ | ++ | ++++ |
| (Hex)_2_ | | C12H24O12 | | [M’-H]^-^ | | 359.1195 | | 4.659 | | | 2.566 | | | 2.044 | | | 0.55 | | | 0.44 | | 0.145 | ++++ | ++++ | ++++ |
| (Hex)_2_ | | C12H24O12 | | [M’+Cl]^-^ | | 395.0962 | | 0.073 | | | 0.000 | | | 0.003 | | | 0.00 | | | 0.04 | | 0.382 | + | + | + |
| Hex+Hex_2_ | | C18H34O17 | | [M’-H]^-^ | | 521.1723 | | 1.088 | | | 0.889 | | | 1.091 | | | 0.82 | | | 1.00 | | 0.876 | ++++ | ++++ | ++++ |
| (Hex)_3_ | | C18H36O18 | | [M’-H]^-^ | | 539.1828 | | 0.007 | | | 0.006 | | | 0.009 | | | 0.89 | | | 1.27 | | 0.948 | + | + | + |
| (Hex)_2_+Hex_2_ | | C24H46O23 | | [M’-H]^-^ | | 701.2357 | | 0.040 | | | 0.020 | | | 0.043 | | | 0.51 | | | 1.06 | | 0.688 | ++ | + | ++ |
| Hex+Hex_4_  Hex_2_+Hex_3_ | | C30H54O25 | | [M’-H]^-^ | | 845.2780 | | 0.005 | | | 0.004 | | | 0.007 | | | 0.93 | | | 1.39 | | 0.798 | + | + | ++ |
| Hex+(Hex_2_)_2_  (Hex)_2_+Hex_3_ | | C30H54O27 | | [M’-H]^-^ | | 863.2885 | | 0.033 | | | 0.037 | | | 0.049 | | | 1.12 | | | 1.47 | | 0.833 | + | + | ++ |
| Hex+Hex_2_+Hex_3_  (Hex)_2_+Hex_4_  (Hex_2_)_3_ | | C36H66O33 | | [M’-H]^-^ | | 1025.3413 | | 0.065 | | | 0.074 | | | 0.088 | | | 1.14 | | | 1.36 | | 0.913 | ++ | ++ | ++ |
| (Hex)_2_+(Hex_2_)_2_ | | C36H66O33 | | [M’-H]^-^ | | 1043.3519 | | 0.245 | | | 0.018 | | | 0.023 | | | 0.07 | | | 0.10 | | 0.263 | ++ | ++ | ++ |
| HexP+Hex | | C12H25O15P | | [M’-H]^-^ | | 439.0856 | | 1.962 | | | 0.941 | | | 0.517 | | | 0.48 | | | 0.26 | | 0.089 | ++++ | ++++ | ++++ |
| HexP+Hex_2_ | | C18H35O20P | | [M’-H]^-^ | | 601.1386 | | 0.028 | | | 0.020 | | | 0.030 | | | 0.70 | | | 1.07 | | 0.745 | +++ | +++ | +++ |
| (Hex_2_)_2_  Hex+Hex_3_ | | C24H44O22 | | [M’-H]^-^ | | 683.2252 | | 0.921 | | | 0.616 | | | 1.198 | | | 0.67 | | | 1.30 | | 0.466 | ++++ | +++ | ++++ |
| (Hex_2_)_2_  Hex+Hex_3_ | | C24H44O22 | | [M’+Cl]^-^ | | 719.2018 | | 0.003 | | | 0.004 | | | 0.015 | | | 1.59 | | | 5.96 | | 0.323 | + | + | + |
|  | |  | |  | |  | |  | | |  | | |  | | |  | | |  | |  |  |  |  |
| H_3_PO_4_+Hex | | C6H14O10P | | [M’-H]^-^ | | 277.0330 | | 1.680^a^ | | | 0.714^ab^ | | | 0.521^b^ | | | 0.43 | | | 0.31 | | 0.013 | ++++ | ++++ | ++++ |
| UDP+Hex | | C15H26N2O18P2 | | [M’-H]^-^ | | 583.0583 | | 0.000 | | | 0.001 | | | 0.003 | | | 2.16 | | | 7.55 | | 0.549 | + | + | + |
|  |  | |  | |  | |  | | |  | |  | | | |  | | |  | |  | |  |  |  |

| (*Continued from previous page*.) | | |  | | |  | |  | | |  | | | |  | | | | |  | |  | | |  | | | |  | | | | | |  | | | | |
| --- | --- | --- | --- | --- | --- | --- | --- | --- | --- | --- | --- | --- | --- | --- | --- | --- | --- | --- | --- | --- | --- | --- | --- | --- | --- | --- | --- | --- | --- | --- | --- | --- | --- | --- | --- | --- | --- | --- | --- |
| Metabolites | | | Molecular  formula | | | Ion type detected  [M=molecule]  [M’=cluster] ^&^ | | Theoretical*  *m/z* | | Relative abundance (%)† | | | | | | | | | | | *P* value | | | | | | Frequency of detection‡ | | | | | | | | | | | | |
|  |  |  |  |  |  |  |  |  |  | N | | B | | W | | B / N | | W / N | | |  |  |  |  |  |  | N | | | | | B | | | | | | W | |
| **Metabolites in TCA cycle** | | |  | | |  | |  | |  | |  | |  | |  | |  | | |  | | | | | | | | |  | | |  | | | | | |  |
| Fumaric acid | | | C4H4O4 | | | [M-H]^-^ | | 115.0037 | | 8.667 | | 8.473 | | 6.985 | | 0.98 | | 0.81 | | | 0.447 | | | | |  | | ++++ | | | ++++ | | | | | | ++++ | | |
| Succinic acid semialdehyde | | | C4H6O3 | | | [M-H]^-^ | | 101.0244 | | 3.367 | | 2.041 | | 2.865 | | 0.61 | | 0.85 | | | 0.619 | | | | |  | | ++++ | | | ++++ | | | | | | ++++ | | |
| Succinic acid | | | C4H6O4 | | | [M-H]^-^ | | 117.0193 | | 0.866 | | 0.926 | | 0.723 | | 1.07 | | 0.83 | | | 0.868 | | | | |  | | ++++ | | | ++++ | | | | | | ++++ | | |
| Malic acid | | | C4H6O5 | | | [M-H]^-^ | | 133.0142 | | 95.833 | | 95.345 | | 84.965 | | 0.99 | | 0.89 | | | 0.141 | | | | |  | | ++++ | | | ++++ | | | | | | ++++ | | |
| α-ketoglutaric acid | | | C5H6O5 | | | [M-H]^-^ | | 145.0142 | | 0.571 | | 1.334 | | 0.960 | | 2.34 | | 1.68 | | | 0.442 | | | | |  | | ++++ | | | ++++ | | | | | | ++++ | | |
| *cis*-Aconitic acid | | | C6H6O6 | | | [M-H]^-^ | | 173.0092 | | 0.365 | | 0.959 | | 1.030 | | 2.63 | | 2.82 | | | 0.333 | | | | |  | | ++++ | | | ++++ | | | | | | ++++ | | |
| Citric acid  Isocitric acid | | | C6H8O7 | | | [M-H]^-^ | | 191.0197 | | 1.212 | | 1.390 | | 1.424 | | 1.15 | | 1.18 | | | 0.908 | | | | |  | | ++++ | | | ++++ | | | | | | ++++ | | |
|  | | |  | | |  | |  | |  | |  | |  | |  | |  | | |  | | | | |  | |  | | |  | | | | | |  | | |
| **Cluster ions** | | |  | | |  | |  | |  | |  | |  | |  | |  | | |  | | | | |  | |  | | |  | | | | | |  | | |
| Fumaric acid+Hex | | | C10H16O10 | | | [M’-H]^-^ | | 295.0671 | | 0.678 | | 0.221 | | 0.215 | | 0.33 | | 0.32 | | | 0.384 | | | | |  | | ++++ | | | ++++ | | | | | | ++++ | | |
| Fumaric acid+Hex_2_ | | | C16H26O15 | | | [M’-H]^-^ | | 457.1199 | | 0.029 | | 0.080 | | 0.077 | | 2.78 | | 2.69 | | | 0.351 | | | | |  | | +++ | | | +++ | | | | | | ++++ | | |
| Succinic acid semialdehyde+Hex | | | C10H18O9 | | | [M’-H]^-^ | | 281.0878 | | 0.022 | | 0.030 | | 0.027 | | 1.38 | | 1.27 | | | 0.882 | | | | |  | | ++ | | | ++ | | | | | | +++ | | |
| Succinic acid+Hex | | | C10H18O10 | | | [M’-H]^-^ | | 297.0827 | | 0.250 | | 0.031 | | 0.030 | | 0.12 | | 0.12 | | | 0.315 | | | | |  | | ++++ | | | +++ | | | | | | ++++ | | |
| Succinic acid+Hex | | | C10H18O10 | | | [M’+Cl]^-^ | | 333.0594 | | 0.089 | | 0.096 | | 0.130 | | 1.08 | | 1.46 | | | 0.780 | | | | |  | | ++++ | | | ++++ | | | | | | ++++ | | |
| Succinic acid+Hex_2_ | | | C16H28O15 | | | [M’-H]^-^ | | 459.1355 | | 0.045 | | 0.004 | | 0.003 | | 0.09 | | 0.06 | | | 0.340 | | | | |  | | ++ | | | ++ | | | | | | ++ | | |
| Malic acid+Hex | | | C10H18O11 | | | [M’-H]^-^ | | 313.0776 | | 3.073 | | 3.780 | | 4.272 | | 1.23 | | 1.39 | | | 0.589 | | | | |  | | ++++ | | | ++++ | | | | | | ++++ | | |
| Malic acid+Hex_2_ | | | C18H30O17 | | | [M’-H]^-^ | | 475.1304 | | 2.025 | | 1.658 | | 2.005 | | 0.82 | | 0.99 | | | 0.843 | | | | |  | | ++++ | | | ++++ | | | | | | ++++ | | |
| Malic acid+Hex_3_ | | | C22H38O21 | | | [M’-H]^-^ | | 637.1833 | | 0.021 | | 0.018 | | 0.023 | | 0.83 | | 1.10 | | | 0.810 | | | | |  | | +++ | | | +++ | | | | | | +++ | | |
| Malic acid+Hex+Hex_3_  Malic acid+(Hex_2_)_2_ | | | C30H52O26 | | | [M’-H]^-^ | | 817.2467 | | 0.039 | | 0.042 | | 0.040 | | 1.09 | | 1.03 | | | 0.989 | | | | |  | | ++ | | | ++ | | | | | | ++ | | |
| α-ketoglutaric acid+Hex | | | C11H18O11 | | | [M’-H]^-^ | | 325.0776 | | 0.103 | | 0.089 | | 0.065 | | 0.87 | | 0.63 | | | 0.580 | | | | |  | | ++++ | | | ++++ | | | | | | ++++ | | |
| *cis*-Aconitic acid +Hex | | | C12H18O12 | | | [M’-H]^-^ | | 353.0726 | | 0.053 | | 0.033 | | 0.017 | | 0.61 | | 0.33 | | | 0.430 | | | | |  | | ++ | | | ++ | | | | | | ++ | | |
|  | | |  | | |  | |  | |  | |  | |  | |  | |  | | |  | | | | |  | |  | | |  | | | | | |  | | |
| **Amino acids** | | |  | | |  | |  | |  | |  | |  | |  | |  | | |  | | | | |  | |  | | |  | | | | | |  | | |
| Alanine | | | C3H7NO2 | | | [M-H]^-^ | | 88.0404 | | 0.030 | | 0.018 | | 0.018 | | 0.59 | | 0.60 | | | 0.838 | | | | |  | | + | | | ++ | | | | | | ++ | | |
| Serine | | | C3H7NO3 | | | [M-H]^-^ | | 104.0353 | | 0.003 | | 0.006 | | 0.006 | | 2.05 | | 1.99 | | | 0.647 | | | | |  | | + | | | + | | | | | | ++ | | |
| Oxoproline | | | C5H7NO3 | | | [M-H]^-^ | | 128.0353 | | 0.485 | | 0.018 | | 0.020 | | 0.04 | | 0.04 | | | 0.383 | | | | |  | | ++ | | | ++ | | | | | | +++ | | |
| Asparagine | | | C4H8N2O3 | | | [M-H]^-^ | | 131.0462 | | 0.006 | | 0.021 | | 0.076 | | 3.63 | | 12.96 | | | 0.169 | | | | |  | | + | | | + | | | | | | ++ | | |
| Aspartic acid | | | C4H7NO4 | | | [M-H]^-^ | | 132.0302 | | 0.721 | | 0.425 | | 0.451 | | 0.59 | | 0.63 | | | 0.641 | | | | |  | | ++++ | | | +++ | | | | | | ++++ | | |
| Glutamine | | | C5H10N2O3 | | | [M-H]^-^ | | 145.0619 | | 0.001 | | 0.001 | | 0.003 | | 0.87 | | 3.71 | | | 0.213 | | | | |  | | + | | | + | | | | | | + | | |
| Glutamic acid | | | C5H9NO4 | | | [M-H]^-^ | | 146.0459 | | 0.071 | | 0.040 | | 0.092 | | 0.56 | | 1.29 | | | 0.587 | | | | |  | | +++ | | | +++ | | | | | | +++ | | |
| Tryptophan | | | C11H12N2O2 | | | [M-H]^-^ | | 203.0826 | | 0.003 | | 0.001 | | 0.004 | | 0.19 | | 1.29 | | | 0.195 | | | | |  | | ++ | | | + | | | | | | ++ | | |
|  | | |  | | |  | |  | |  | |  | |  | |  | |  | | |  | | | | |  | |  | | |  | | | | | |  | | |
| **Cluster ions** | | |  | | |  | |  | |  | |  | |  | |  | |  | | |  | | | | |  | |  | | |  | | | | | |  | | |
| Alanine+Hex | | | C9H20NO8 | | | [M’-H]^-^ | | 268.1038 | | 0.004 | | 0.000 | | 0.008 | | 0.07 | | 1.70 | | | 0.195 | | | | |  | | + | | | + | | | | | | ++ | | |
| Alanine+Hex_2_ | | | C15H29NO13 | | | [M’-H]^-^ | | 430.1566 | | 0.000 | | 0.000 | | 0.002 | | 0.87 | | 6.03 | | | 0.119 | | | | |  | | + | | | + | | | | | | + | | |
| Serine+Hex | | | C9H19NO9 | | | [M’-H]^-^ | | 284.0987 | | 0.040 | | 0.004 | | 0.004 | | 0.09 | | 0.09 | | | 0.395 | | | | |  | | + | | | + | | | | | | + | | |
| Serine+Hex_2_ | | | C15H29NO13 | | | [M’-H]^-^ | | 446.1515 | | 0.002 | | 0.002 | | 0.006 | | 0.96 | | 2.79 | | | 0.316 | | | | |  | | + | | | + | | | | | | ++ | | |
| Proline+Hex | | | C11H21NO8 | | | [M’-H]^-^ | | 294.1194 | | 0.001 | | 0.000 | | 0.000 | | - | | 0.64 | | | 0.581 | | | | |  | | + | | | - | | | | | | + | | |
| Oxoproline+Hex | | | C11H19NO9 | | | [M’-H]^-^ | | 308.0987 | | 0.070 | | 0.002 | | 0.003 | | 0.04 | | 0.04 | | | 0.374 | | | | |  | | ++ | | | + | | | | | | + | | |
| Oxoproline+Hex_2_ | | | C17H29NO14 | | | [M’-H]^-^ | | 470.1515 | | 0.002 | | 0.009 | | 0.002 | | 4.27 | | 0.76 | | | 0.505 | | | | |  | | + | | | + | | | | | | + | | |
| Leucine+Hex  Isoleucine+Hex | | | C12H23NO8 | | | [M’-H]^-^ | | 310.1507 | | 0.002 | | 0.000 | | 0.004 | | - | | 2.18 | | | 0.413 | | | | |  | | + | | | - | | | | | | + | | |
| Asparagine+Hex | | | C10H20N2O9 | | | [M’-H]^-^ | | 311.1096 | | 0.007 | | 0.005 | | 0.016 | | 0.65 | | 2.25 | | | 0.428 | | | | |  | | + | | | + | | | | | | ++ | | |
| Asparagine+Hex_2_ | | | C16H30N2O14 | | | [M’-H]^-^ | | 473.1624 | | 0.016 | | 0.008 | | 0.031 | | 0.49 | | 2.01 | | | 0.290 | | | | |  | | + | | | + | | | | | | ++ | | |
| Aspartic acid+Hex | | | C10H19NO10 | | | [M’-H]^-^ | | 312.0936 | | 0.074 | | 0.045 | | 0.030 | | 0.60 | | 0.41 | | | 0.502 | | | | |  | | ++ | | | ++ | | | | | | ++ | | |
| Aspartic acid+Hex_2_ | | | C16H29NO15 | | | [M’-H]^-^ | | 474.1464 | | 0.179 | | 0.105 | | 0.121 | | 0.59 | | 0.68 | | | 0.712 | | | | |  | | +++ | | | ++ | | | | | | ++++ | | |
| Aspartic acid+(Hex)_2_ | | | C16H31NO16 | | | [M’-H]^-^ | | 492.1570 | | 0.012 | | 0.009 | | 0.007 | | 0.75 | | 0.54 | | | 0.887 | | | | |  | | + | | | ++ | | | | | | + | | |
| Aspartic acid+Hex+Hex_2_ | | | C22H41NO21 | | | [M’-H]^-^ | | 654.2098 | | 0.001 | | 0.001 | | 0.000 | | 1.39 | | - | | | 0.567 | | | | |  | | + | | | + | | | | | | - | | |
| Aspartic acid+(Hex_2_)_2_  Aspartic acid+Hex+Hex_3_ | | | C28H52O24 | | | [M’-H]^-^ | | 816.2627 | | 0.002 | | 0.008 | | 0.009 | | 3.52 | | 3.65 | | | 0.622 | | | | |  | | + | | | + | | | | | | + | | |
| Glutamic acid+Hex | | | C11H21NO10 | | | [M’-H]^-^ | | 326.1093 | | 0.003 | | 0.003 | | 0.005 | | 0.98 | | 1.61 | | | 0.767 | | | | |  | | + | | | + | | | | | | ++ | | |
| Glutamic acid+Hex_2_ | | | C17H31NO15 | | | [M’-H]^-^ | | 488.1621 | | 0.012 | | 0.016 | | 0.017 | | 1.31 | | 1.40 | | | 0.876 | | | | |  | | ++ | | | ++ | | | | | | +++ | | |
|  |  |  | |  |  | |  | |  | | | |  | | | |  | |  | | | |  |  | | | | | | | | | |  | |  | | | |

| (*Continued from previous page*.) | | | |  | |  | | |  | | |  | |  | | | | | | | | | | | | |  | | | | |  | | | | |  | | |
| --- | --- | --- | --- | --- | --- | --- | --- | --- | --- | --- | --- | --- | --- | --- | --- | --- | --- | --- | --- | --- | --- | --- | --- | --- | --- | --- | --- | --- | --- | --- | --- | --- | --- | --- | --- | --- | --- | --- | --- |
| Metabolites | | | | Molecular  formula | | | | Ion type detected  [M=molecule]  [M’=cluster] ^&^ | | | Theoretical*  *m/z* | | Relative abundance (%)† | | | | | | | | | | | | | *P* value | | | | | Frequency of detection‡ | | | | | | | | |
|  |  |  |  |  |  |  |  |  |  |  |  |  | N | | | B | | W | | | B / N | | | W / N | |  |  |  |  |  | N | | | B | | | | | W |
| **Saccharides** | | | |  | | | |  | | |  | |  | | | |  | | |  | | |  | |  | | | | |  | | |  | | | | |  | |
|  | | | |  | | | |  | | |  | |  | | |  | |  | | |  | | |  | |  | | | | |  | | | |  | | | | |
| **Monosaccharide** | | | |  | | | |  | | |  | |  | | |  | |  | | |  | | |  | |  | | | | |  | | |  | | | | |  |
| Tetrose | | | | C4H8O4 | | | | [M-H]^-^ | | | 119.0350 | | 1.751 | | | 1.687 | | 1.495 | | | 0.96 | | | 0.85 | | 0.905 | | | | | ++++ | | | ++++ | | | | | ++++ |
| Pentose | | | | C5H10O5 | | | | [M-H]^-^ | | | 149.0455 | | 0.671 | | | 0.792 | | 0.644 | | | 1.18 | | | 0.96 | | 0.811 | | | | | ++++ | | | ++++ | | | | | ++++ |
| Heptose | | | | C7H14O7 | | | | [M-H]^-^ | | | 209.0667 | | 0.144 | | | 0.196 | | 0.247 | | | 1.37 | | | 1.72 | | 0.449 | | | | | ++++ | | | ++++ | | | | | ++++ |
| Octose | | | | C8H16O8 | | | | [M-H]^-^ | | | 239.0772 | | 0.378 | | | 0.457 | | 0.537 | | | 1.21 | | | 1.42 | | 0.604 | | | | | ++++ | | | ++++ | | | | | ++++ |
|  | | | |  | | | |  | | |  | |  | | |  | |  | | |  | | |  | |  | | | | |  | | |  | | | | |  |
| **Cluster ions** | | | |  | | | |  | | |  | |  | | |  | |  | | |  | | |  | |  | | | | |  | | |  | | | | |  |
| Tetrose+Hex | | | | C10H20O10 | | | | [M’-H]^-^ | | | 299.0984 | | 0.015 | | | 0.069 | | 0.010 | | | 4.59 | | | 0.69 | | 0.129 | | | | | ++ | | | ++ | | | | | ++ |
| Tetrose+Hex | | | | C10H20O10 | | | | [M’+Cl]^-^ | | | 335.0751 | | 0.011 | | | 0.027 | | 0.007 | | | 2.38 | | | 0.62 | | 0.157 | | | | | ++ | | | ++ | | | | | ++ |
| Tetrose+Hex_2_ | | | | C16H30O15 | | | | [M’-H]^-^ | | | 461.1512 | | 0.081 | | | 0.259 | | 0.273 | | | 3.18 | | | 3.34 | | 0.142 | | | | | ++ | | | ++++ | | | | | +++ |
| Pentose+Hex | | | | C11H22O11 | | | | [M’-H]^-^ | | | 329.1089 | | 0.028 | | | 0.016 | | 0.012 | | | 0.56 | | | 0.42 | | 0.340 | | | | | ++ | | | +++ | | | | | +++ |
| Pentose+Hex_2_ | | | | C17H32O16 | | | | [M’-H]^-^ | | | 491.1617 | | 0.002 | | | 0.003 | | 0.004 | | | 1.60 | | | 2.23 | | 0.731 | | | | | + | | | + | | | | | ++ |
| Heptose+Hex | | | | C13H26O13 | | | | [M’-H]^-^ | | | 389.1301 | | 0.016 | | | 0.035 | | 0.027 | | | 2.17 | | | 1.65 | | 0.655 | | | | | ++ | | | +++ | | | | | +++ |
| Heptose+Hex_2_ | | | | C19H36O18 | | | | [M’-H]^-^ | | | 551.1829 | | 0.001 | | | 0.000 | | 0.002 | | | - | | | 1.87 | | 0.255 | | | | | + | | | - | | | | | + |
| Octose+Hex | | | | C14H28O14 | | | | [M’-H]^-^ | | | 419.1406 | | 0.006 | | | 0.006 | | 0.011 | | | 0.91 | | | 1.76 | | 0.617 | | | | | ++ | | | + | | | | | ++ |
| Octose+Hex_2_ | | | | C20H28O19 | | | | [M’-H]^-^ | | | 581.1934 | | 0.008 | | | 0.009 | | 0.019 | | | 1.07 | | | 2.28 | | 0.440 | | | | | + | | | + | | | | | ++ |
| Octose+Hex_3_ | | | | C26H48O24 | | | | [M’-H]^-^ | | | 743.2463 | | 0.007 | | | 0.018 | | 0.026 | | | 2.44 | | | 3.52 | | 0.370 | | | | | + | | | ++ | | | | | ++ |
|  | | | |  | | | |  | | |  | |  | | |  | |  | | |  | | |  | |  | | | | |  | | |  | | | | |  |
| **Oligosaccharide** | | | |  | | | |  | | |  | |  | | |  | |  | | |  | | |  | |  | | | | |  | | |  | | | | |  |
| Hex_3_ | | | | C18H32O16 | | | | [M-H]^-^ | | | 503.1618 | | 0.022 | | | 0.011 | | 0.025 | | | 0.50 | | | 1.18 | | 0.276 | | | | | +++ | | | ++ | | | | | ++++ |
|  | | | |  | | | |  | | |  | |  | | |  | |  | | |  | | |  | |  | | | | |  | | |  | | | | |  |
| **Sugar alcohols** | | | |  | | | |  | | |  | |  | | |  | |  | | |  | | |  | |  | | | | |  | | |  | | | | |  |
| Hexitol | | | | C6H14O6 | | | | [M-H]^-^ | | | 181.0718 | | 15.850 | | | 20.146 | | 31.409 | | | 1.27 | | | 1.98 | | 0.223 | | | | | ++++ | | | ++++ | | | | | ++++ |
| Hexitol | | | | C6H14O6 | | | | [M+Cl]^-^ | | | 217.0484 | | 0.332 | | | 0.315 | | 0.962 | | | 0.95 | | | 2.90 | | 0.474 | | | | | ++++ | | | ++++ | | | | | ++++ |
|  | | | |  | | | |  | | |  | |  | | |  | |  | | |  | | |  | |  | | | | |  | | |  | | | | |  |
| **Cluster ions** | | | |  | | | |  | | |  | |  | | |  | |  | | |  | | |  | |  | | | | |  | | |  | | | | |  |
| Hexitol+Hex | | | | C12H26O12 | | | | [M’-H]^-^ | | | 361.1352 | | 0.884 | | | 0.843 | | 0.989 | | | 0.95 | | | 1.12 | | 0.924 | | | | | ++++ | | | ++++ | | | | | ++++ |
| Hexitol+Hex_2_ | | | | C18H36O17 | | | | [M’-H]^-^ | | | 523.1880 | | 0.152 | | | 0.167 | | 0.310 | | | 1.09 | | | 2.03 | | 0.425 | | | | | +++ | | | +++ | | | | | ++++ |
| Hexitol+Hex_3_ | | | | C24H46O22 | | | | [M’-H]^-^ | | | 685.2409 | | 0.002 | | | 0.000 | | 0.003 | | | 0.09 | | | 1.42 | | 0.318 | | | | | + | | | + | | | | | ++ |
|  | | | |  | | | |  | | |  | |  | | |  | |  | | |  | | |  | |  | | | | |  | | |  | | | | |  |
| **Deoxy sugar** | | | |  | | | |  | | |  | |  | | |  | |  | | |  | | |  | |  | | | | |  | | |  | | | | |  |
| Deoxyhexose | | | | C6H12O5 | | | | [M-H]^-^ | | | 163.0612 | | 4.336 | | | 15.413 | | 12.655 | | | 3.55 | | | 2.92 | | 0.082 | | | | | ++++ | | | ++++ | | | | | ++++ |
|  | | | |  | | | |  | | |  | |  | | |  | |  | | |  | | |  | |  | | | | |  | | |  | | | | |  |
| **Cluster ions** | | | |  | | | |  | | |  | |  | | |  | |  | | |  | | |  | |  | | | | |  | | |  | | | | |  |
| Deoxyhexose+Hex | | | | C12H24O11 | | | | [M’-H]^-^ | | | 343.1246 | | 0.060 | | | 0.026 | | 0.058 | | | 0.44 | | | 0.97 | | 0.604 | | | | | +++ | | | +++ | | | | | +++ |
| Deoxyhexose+Hex | | | | C12H24O11 | | | | [M’+Cl]^-^ | | | 379.1013 | | 0.001 | | | 0.005 | | 0.011 | | | 9.25 | | | 20.63 | | 0.229 | | | | | + | | | + | | | | | ++ |
|  | | | |  | | | |  | | |  | |  | | |  | |  | | |  | | |  | |  | | | | |  | | |  | | | | |  |
| **Saccharic acids** | | | |  | | | |  | | |  | |  | | |  | |  | | |  | | |  | |  | | | | |  | | |  | | | | |  |
| Glyceric acid | | | | C3H6O4 | | | | [M-H]^-^ | | | 105.0193 | | 0.473 | | | 0.777 | | 2.876 | | | 1.64 | | | 6.09 | | 0.423 | | | | | ++++ | | | ++++ | | | | | ++++ |
| Threonic acid | | | | C4H8O5 | | | | [M-H]^-^ | | | 135.0299 | | 0.357 | | | 0.462 | | 0.476 | | | 1.29 | | | 1.33 | | 0.796 | | | | | ++++ | | | ++++ | | | | | ++++ |
| Galacturonic acid | | | | C6H10O7 | | | | [M-H]^-^ | | | 193.0354 | | 0.492 | | | 0.700 | | 0.415 | | | 1.42 | | | 0.84 | | 0.594 | | | | | ++++ | | | ++++ | | | | | ++++ |
| Gluconic acid | | | | C6H12O7 | | | | [M-H]^-^ | | | 195.0510 | | 0.154 | | | 0.201 | | 0.198 | | | 1.31 | | | 1.28 | | 0.798 | | | | | ++++ | | | ++++ | | | | | ++++ |
| Galactaric acid | | | | C6H10O8 | | | | [M-H]^-^ | | | 209.0303 | | 0.067 | | | 0.074 | | 0.251 | | | 1.09 | | | 3.74 | | 0.526 | | | | | +++ | | | ++++ | | | | | ++++ |
|  | | | |  | | | |  | | |  | |  | | |  | |  | | |  | | |  | |  | | | | |  | | |  | | | | |  |
| **Cluster ions** | | | |  | | | |  | | |  | |  | | |  | |  | | |  | | |  | |  | | | | |  | | |  | | | | |  |
| Glyceric acid+Hex | | | | C9H18O10 | | | | [M’-H]^-^ | | | 285.0827 | | 0.027 | | | 0.025 | | 0.016 | | | 0.92 | | | 0.59 | | 0.805 | | | | | ++ | | | ++ | | | | | ++ |
| Glyceric acid+Hex_2_ | | | | C15H28O15 | | | | [M’-H]^-^ | | | 447.1355 | | 0.060 | | | 0.057 | | 0.044 | | | 0.96 | | | 0.74 | | 0.694 | | | | | +++ | | | ++++ | | | | | ++++ |
| Threonic acid+Hex | | | | C10H20O11 | | | | [M’-H]^-^ | | | 315.0933 | | 0.655 | | | 0.483 | | 0.679 | | | 0.74 | | | 1.04 | | 0.835 | | | | | ++++ | | | ++++ | | | | | ++++ |
| Threonic acid+Hex | | | | C10H20O11 | | | | [M’+Cl]^-^ | | | 351.0700 | | 0.004 | | | 0.004 | | 0.016 | | | 1.00 | | | 3.84 | | 0.086 | | | | | ++ | | | ++ | | | | | ++ |
| Galacturonic acid+Hex | | | | C12H22O13 | | | | [M’-H]^-^ | | | 373.0988 | | 0.012 | | | 0.009 | | 0.005 | | | 0.73 | | | 0.39 | | 0.351 | | | | | ++ | | | ++ | | | | | ++ |
| Galacturonic acid+Hex_2_ | | | | C18H32O18 | | | | [M’-H]^-^ | | | 535.1516 | | 0.033 | | | 0.011 | | 0.015 | | | 0.33 | | | 0.44 | | 0.188 | | | | | ++ | | | ++ | | | | | +++ |
| Gluconic acid+Hex | | | | C12H24O13 | | | | [M’-H]^-^ | | | 375.1144 | | 0.021 | | | 0.015 | | 0.006 | | | 0.72 | | | 0.30 | | 0.218 | | | | | +++ | | | +++ | | | | | +++ |
| Gluconic acid+Hex | | | | C12H24O13 | | | | [M’+Cl]^-^ | | | 411.0911 | | 0.001 | | | 0.006 | | 0.005 | | | 6.27 | | | 6.01 | | 0.377 | | | | | + | | | ++ | | | | | ++ |
| Gluconic acid+Hex_2_ | | | | C18H34O18 | | | | [M’-H]^-^ | | | 537.1672 | | 0.006 | | | 0.009 | | 0.002 | | | 1.54 | | | 0.43 | | 0.470 | | | | | ++ | | | + | | | | | ++ |
|  |  |  |  | |  | |  | | |  | | | | |  | | | |  | | |  | | | | | |  |  | |  | | | | |  | | | |

| (*Continued from previous page*.) |  |  |  | | | | | |  | |  |  | | | | | | | | | | |
| --- | --- | --- | --- | --- | --- | --- | --- | --- | --- | --- | --- | --- | --- | --- | --- | --- | --- | --- | --- | --- | --- | --- |
| Metabolites | Molecular  formula | Ion type detected  [M=molecule]  [M’=cluster] ^&^ | Theoretical*  *m/z* | Relative abundance (%)† | | | | | | *P* value | | | Frequency of detection‡ | | | | | | | | | |
|  |  |  |  | N | B | W | B / N | W / N | |  |  |  |  | N | | | B | | | W | | |
| **Alcohols** |  |  |  |  |  |  |  |  | |  | | |  |  | | | |  | | |  | |
| Acetol | C3H6O2 | [M-H]^-^ | 73.0295 | 0.308 | 0.374 | 0.248 | 1.21 | 0.81 | | 0.733 | | |  | ++++ | | | | ++++ | | | ++++ | |
| Butanol | C4H10O | [M-H]^-^ | 73.0659 | 0.014 | 0.064 | 0.018 | 4.58 | 1.32 | | 0.203 | | |  | ++++ | | | | ++++ | | | ++++ | |
| Pentanol | C5H12O | [M-H]^-^ | 87.0815 | 0.006 | 0.035 | 0.015 | 5.63 | 2.52 | | 0.121 | | |  | ++ | | | | ++++ | | | ++ | |
| Hexanol | C6H14O | [M-H]^-^ | 101.0972 | 0.000 | 0.002 | 0.005 | - | - | | 0.344 | | |  | - | | | | + | | | + | |
| Phenylethyl alcohol | C8H10O | [M-H]^-^ | 121.0659 | 0.043 | 0.110 | 0.060 | 2.53 | 1.39 | | 0.193 | | |  | ++++ | | | | ++++ | | | ++++ | |
|  |  |  |  |  |  |  |  |  | |  | | |  |  | | | |  | | |  | |
| **Esters** |  |  |  |  |  |  |  |  | |  | | |  |  | | | |  | | |  | |
| Ethyl formate | C3H6O2 | [M-H]^-^ | 73.0295 | 0.305 | 0.358 | 0.249 | 1.17 | 0.82 | | 0.791 | | |  | ++++ | | | | ++++ | | | ++++ | |
| Ethyl acetate | C4H8O2 | [M-H]^-^ | 87.0452 | 0.368 | 0.600 | 0.340 | 1.63 | 0.92 | | 0.576 | | |  | +++ | | | | ++++ | | | ++++ | |
| Methyl butanoate  Ethyl propanoate  Propyl acetate | C5H10O2 | [M-H]^-^ | 101.0608 | 0.209 | 0.355 | 0.251 | 1.70 | 1.20 | | 0.320 | | |  | ++++ | | | | ++++ | | | ++++ | |
| Methyl acetoacetate | C5H8O3 | [M-H]^-^ | 115.0401 | 0.166 | 0.228 | 0.204 | 1.38 | 1.23 | | 0.750 | | |  | ++++ | | | | ++++ | | | ++++ | |
| Ethyl butanoate  Propyl propanoate  Butyl acetate | C6H12O2 | [M-H]^-^ | 115.0765 | 0.487 | 0.772 | 0.576 | 1.58 | 1.18 | | 0.324 | | |  | ++++ | | | | ++++ | | | ++++ | |
| Methylbutyl acetate | C7H14O2 | [M-H]^-^ | 129.0921 | 0.189 | 0.367 | 0.256 | 1.94 | 1.35 | | 0.087 | | |  | ++++ | | | | ++++ | | | ++++ | |
| Dimethyl fumarate | C6H8O4 | [M-H]^-^ | 143.0350 | 3.844 | 2.754 | 3.011 | 0.72 | 0.78 | | 0.692 | | |  | ++++ | | | | ++++ | | | ++++ | |
| Ethyl hexanoate  Propyl methyl butanoate  Butyl butanoate  Hexyl acetate | C8H16O2 | [M-H]^-^ | 143.1078 | 0.794 | 1.243 | 0.975 | 1.56 | 1.23 | | 0.377 | | |  | ++++ | | | | ++++ | | | ++++ | |
| Propyl hexanoate  Butyl methyl butanoate | C9H18O2 | [M-H]^-^ | 157.1234 | 0.703 | 1.083 | 0.864 | 1.54 | 1.23 | | 0.452 | | |  | ++++ | | | | ++++ | | | ++++ | |
| Dimethyl malate | C6H10O5 | [M-H]^-^ | 161.0455 | 3.787 | 3.996 | 3.354 | 1.06 | 0.89 | | 0.875 | | |  | ++++ | | | | ++++ | | | ++++ | |
| Butyl hexanoate  Hexyl butanoate | C10H20O2 | [M-H]^-^ | 171.1391 | 0.568 | 0.878 | 0.736 | 1.55 | 1.30 | | 0.487 | | |  | ++++ | | | | ++++ | | | ++++ | |
| Gluconolactone | C6H10O6 | [M-H]^-^ | 177.0405 | 2.398 | 3.350 | 2.269 | 1.40 | 0.95 | | 0.547 | | |  | ++++ | | | | ++++ | | | ++++ | |
| Hexyl methylbutanoate | C11H22O2 | [M-H]^-^ | 185.1547 | 0.090 | 0.197 | 0.106 | 2.18 | 1.17 | | 0.112 | | |  | ++++ | | | | ++++ | | | ++++ | |
| Hexyl hexanoate | C12H24O2 | [M-H]^-^ | 199.1704 | 0.258 | 0.673 | 0.305 | 2.61 | 1.18 | | 0.094 | | |  | ++++ | | | | ++++ | | | ++++ | |
| Galacturonic acid methyl ester | C7H12O7 | [M-H]^-^ | 207.0510 | 0.245 | 0.251 | 0.192 | 1.02 | 0.78 | | 0.843 | | |  | ++++ | | | | ++++ | | | ++++ | |
|  |  |  |  |  |  |  |  |  | |  | | |  |  | | | |  | | |  | |
| **Cluster ions** |  |  |  |  |  |  |  |  | |  | | |  |  | | | |  | | |  | |
| Ethyl formate+Hex | C9H18O8 | [M’-H]^-^ | 253.0929 | 0.014 | 0.005 | 0.009 | 0.38 | 0.68 | | 0.382 | | |  | ++ | | | | ++ | | | +++ | |
| Ethyl formate+Hex_2_ | C15H28O13 | [M’-H]^-^ | 415.1457 | 0.000 | 0.008 | 0.003 | 16.98 | 5.24 | | 0.095 | | |  | + | | | | + | | | ++ | |
| Ethyl acetate+Hex | C10H20O8 | [M’-H]^-^ | 267.1086 | 0.009 | 0.012 | 0.005 | 1.31 | 0.49 | | 0.442 | | |  | ++ | | | | ++ | | | ++ | |
| Ethyl acetate+Hex_2_ | C16H30O13 | [M’-H]^-^ | 429.1614 | 0.001 | 0.002 | 0.002 | 1.29 | 1.62 | | 0.765 | | |  | + | | | | + | | | ++ | |
| Methyl butanoate+Hex  Ethyl propanoate+Hex  Propyl acetate+Hex | C11H22O8 | [M’-H]^-^ | 281.1242 | 0.006 | 0.006 | 0.003 | 0.92 | 0.47 | | 0.664 | | |  | ++ | | | | ++ | | | + | |
| Methyl butanoate+Hex_2_  Ethyl propanoate+Hex_2_  Propyl acetate+Hex_2_ | C17H32O13 | [M’-H]^-^ | 443.1770 | 0.000 | 0.001 | 0.001 | 5.10 | 5.92 | | 0.453 | | |  | + | | | | + | | | + | |
| Methyl butanoate+Hex_2_  Ethyl propanoate+Hex_2_  Propyl acetate+Hex_2_ | C17H32O13 | [M’+Cl]^-^ | 479.1537 | 0.002 | 0.025 | 0.016 | 10.08 | 6.47 | | 0.261 | | |  | + | | | | ++ | | | ++ | |
| Methyl acetoacetate+Hex | C11H20O9 | [M’-H]^-^ | 295.1035 | 0.016^a^ | 0.002^b^ | 0.004^ab^ | 0.10 | 0.24 | | 0.040 | | |  | ++ | | | | + | | | ++ | |
| Ethyl butanoate+Hex  Propyl propanoate+Hex  Butyl acetate+Hex | C12H24O8 | [M’-H]^-^ | 295.1399 | 0.005 | 0.005 | 0.002 | 1.02 | 0.33 | | 0.642 | | |  | ++ | | | | + | | | + | |
| Dimethyl fumarate+Hex | C12H20O10 | [M’-H]^-^ | 323.0984 | 0.076 | 0.042 | 0.037 | 0.56 | 0.48 | | 0.546 | | |  | +++ | | | | +++ | | | +++ | |
|  |  |  |  |  |  |  |  |  | |  | | | | |  |  | | |  | | |  |

| (*Continued from previous page*.) |  |  |  | | | | | |  | |  |  | | | | | | | | | | |
| --- | --- | --- | --- | --- | --- | --- | --- | --- | --- | --- | --- | --- | --- | --- | --- | --- | --- | --- | --- | --- | --- | --- |
| Metabolites | Molecular  formula | Ion type detected  [M=molecule]  [M’=cluster] ^&^ | Theoretical*  *m/z* | Relative abundance (%)† | | | | | | *P* value | | | Frequency of detection‡ | | | | | | | | | |
|  |  |  |  | N | B | W | B / N | W / C | |  |  |  |  | N | | | B | | | W | | |
| Ethyl hexanoate+Hex  Propyl methyl butanoate+Hex  Butyl butanoate+Hex  Hexyl acetate+Hex | C14H28O8 | [M’-H]^-^ | 323.1712 | 0.006 | 0.005 | 0.007 | 0.79 | 1.12 | | 0.863 | | |  | ++ | | | | ++ | | | ++ | |
| Ethyl hexanoate+Hex_2_  Propyl methyl butanoate+Hex_2_  Butyl butanoate+Hex_2_  Hexyl acetate+Hex_2_ | C20H38O13 | [M’-H]^-^ | 485.2240 | 0.002 | 0.003 | 0.000 | 1.84 | 0.27 | | 0.572 | | |  | + | | | | + | | | + | |
| Butyl hexanoate+Hex  Hexyl butanoate+Hex | C16H32O8 | [M’-H]^-^ | 351.2025 | 0.006^a^ | 0.001^ab^ | 0.001^b^ | 0.17 | 0.09 | | 0.046 | | |  | ++ | | | | + | | | + | |
| Gluconolactone+Hex | C12H22O12 | [M’-H]^-^ | 357.1039 | 0.132 | 0.183 | 0.103 | 1.38 | 0.78 | | 0.357 | | |  | ++++ | | | | ++++ | | | ++++ | |
| Gluconolactone+Hex_2_ | C18H32O17 | [M’-H]^-^ | 519.1567 | 0.021 | 0.005 | 0.012 | 0.22 | 0.56 | | 0.234 | | |  | +++ | | | | ++ | | | ++ | |
| Gluconolactone+Hex_3_ | C24H42O22 | [M’-H]^-^ | 681.2096 | 0.011 | 0.006 | 0.007 | 0.58 | 0.62 | | 0.777 | | |  | ++ | | | | + | | | ++ | |
| Galacturonic acid methyl ester+Hex | C13H24O13 | [M’-H]^-^ | 387.1144 | 0.188 | 0.290 | 0.407 | 1.54 | 2.16 | | 0.212 | | |  | ++++ | | | | ++++ | | | ++++ | |
| Hexyl hexanoate+Hex | C18H36O8 | [M’-H]^-^ | 253.0929 | 0.017 | 0.007 | 0.011 | 0.45 | 0.68 | | 0.350 | | |  | ++ | | | | ++ | | | +++ | |
|  |  |  |  |  |  |  |  |  | |  | | |  |  | | | |  | | |  | |
| **Aldehydes** |  |  |  |  |  |  |  |  | |  | | |  |  | | | |  | | |  | |
| Glycidaldehyde | C3H4O2 | [M-H]^-^ | 71.0139 | 1.887 | 1.203 | 1.749 | 0.64 | 0.93 | | 0.556 | | |  | ++++ | | | | ++++ | | | ++++ | |
| Butanal | C4H8O | [M-H]^-^ | 71.0502 | 0.008 | 0.006 | 0.009 | 0.70 | 1.09 | | 0.802 | | |  | ++ | | | | +++ | | | ++ | |
| Furfural | C5H4O2 | [M-H]^-^ | 95.0139 | 0.208 | 0.209 | 0.208 | 1.01 | 1.00 | | 1.000 | | |  | ++++ | | | | ++++ | | | ++++ | |
| Hexenal | C6H10O | [M-H]^-^ | 97.0659 | 0.011 | 0.036 | 0.004 | 3.22 | 0.36 | | 0.418 | | |  | +++ | | | | ++ | | | +++ | |
| Hexanal | C6H12O | [M-H]^-^ | 99.0815 | 0.000 | 0.005 | 0.002 | 22.24 | 11.18 | | 0.051 | | |  | + | | | | ++ | | | + | |
| Succinic acid semialdehyde | C4H6O3 | [M-H]^-^ | 101.0244 | 3.367 | 2.041 | 2.865 | 0.61 | 0.85 | | 0.619 | | |  | ++++ | | | | ++++ | | | ++++ | |
| Benzaldehyde | C7H6O | [M-H]^-^ | 105.0346 | 0.003 | 0.000 | 0.006 | 0.14 | 2.06 | | 0.323 | | |  | + | | | | + | | | ++ | |
| Heptenal | C7H12O | [M-H]^-^ | 111.0815 | 0.002 | 0.075 | 0.010 | 32.97 | 4.51 | | 0.088 | | |  | ++ | | | | +++ | | | ++ | |
| Octanal | C8H16O | [M-H]^-^ | 127.1128 | 0.006 | 0.034 | 0.014 | 6.12 | 2.49 | | 0.206 | | |  | + | | | | ++ | | | ++ | |
| Nonanal | C9H18O | [M-H]^-^ | 141.1285 | 0.007 | 0.047 | 0.011 | 7.07 | 1.65 | | 0.316 | | |  | ++ | | | | ++ | | | + | |
| Decanal | C10H20O | [M-H]^-^ | 155.1441 | 0.001 | 0.009 | 0.004 | 8.42 | 4.02 | | 0.307 | | |  | + | | | | ++ | | | + | |
|  |  |  |  |  |  |  |  |  | |  | | |  |  | | | |  | | |  | |
| **Cluster ions** |  |  |  |  |  |  |  |  | |  | | |  |  | | | |  | | |  | |
| Glycidaldehyde+Hex | C9H16O8 | [M’-H]^-^ | 251.0773 | 0.050 | 0.036 | 0.059 | 0.71 | 1.16 | | 0.687 | | |  | +++ | | | | +++ | | | +++ | |
|  |  |  |  |  |  |  |  |  | |  | | |  |  | | | |  | | |  | |
| **Ketone** |  |  |  |  |  |  |  |  | |  | | |  |  | | | |  | | |  | |
| Methylheptenone | C8H14O | [M-H]^-^ | 125.0972 | 0.003 | 0.022 | 0.007 | 6.68 | 2.15 | | 0.151 | | |  | + | | | | ++ | | | ++ | |
|  |  |  |  |  |  |  |  |  | |  | | |  |  | | | |  | | |  | |
| **Phenylpropene** |  |  |  |  |  |  |  |  | |  | | |  |  | | | |  | | |  | |
| Estragole | C10H12O | [M-H]^-^ | 147.0815 | 0.005 | 0.028 | 0.004 | 5.76 | 0.79 | | 0.096 | | |  | + | | | | +++ | | | ++ | |
|  |  |  |  |  |  |  |  |  | |  | | |  |  | | | |  | | |  | |
| **Terpene** |  |  |  |  |  |  |  |  | |  | | |  |  | | | |  | | |  | |
| Farnesene | C15H24 | [M-H]^-^ | 203.1805 | 0.000 | 0.002 | 0.005 | - | - | | 0.267 | | |  | - | | | | + | | | + | |
|  |  |  |  |  |  |  |  |  | |  | | |  |  | | | |  | | |  | |
| **Lipid** |  |  |  |  |  |  |  |  | |  | | |  |  | | | |  | | |  | |
| Phosphatidyl glycerol | C6H15O8P | [M-H]^-^ | 245.0432 | 0.064 | 0.086 | 0.065 | 1.33 | 1.01 | | 0.622 | | |  | +++ | | | | ++++ | | | +++ | |
|  |  |  |  |  |  |  |  |  | |  | | |  |  | | | |  | | |  | |
| **Cluster ion** |  |  |  |  |  |  |  |  | |  | | |  |  | | | |  | | |  | |
| Phosphatidyl glycerol+Hex | C12H27O14P | [M’-H]^-^ | 425.1066 | 0.000 | 0.003 | 0.002 | 10.14 | 5.56 | | 0.229 | | |  | + | | | | + | | | + | |
|  |  |  |  |  |  |  |  |  | |  | | | | |  |  | | |  | | |  |

| (*Continued from previous page*.) |  |  |  | | | | | |  | |  |  | | | | | | |
| --- | --- | --- | --- | --- | --- | --- | --- | --- | --- | --- | --- | --- | --- | --- | --- | --- | --- | --- |
| Metabolites | Molecular  formula | Ion type detected  [M=molecule]  [M’=cluster] ^&^ | Theoretical*  *m/z* | Relative abundance (%)† | | | | | | *P* value | | | Frequency of detection‡ | | | | | |
|  |  |  |  | N | B | W | B / N | W / N | |  |  |  |  | N | | B | | W |
| **Cell wall synthesis metabolism** |  |  |  |  |  |  |  |  | |  | | |  | |  | |  | |
| Glycerol | C3H8O3 | [M-H]^-^ | 91.0401 | 0.011 | 0.037 | 0.030 | 3.39 | 2.81 | | 0.232 | | | ++ | | ++++ | | +++ | |
| Glycerol | C3H8O3 | [M+Cl]^-^ | 127.0167 | 0.000 | 0.001 | 0.001 | - | - | | 0.583 | | | - | | + | | + | |
| Coumaryl-alcohol | C9H10O2 | [M-H]^-^ | 149.0608 | 0.026 | 0.051 | 0.036 | 1.98 | 1.40 | | 0.421 | | | +++ | | ++++ | | +++ | |
| Shikimic acid | C7H10O5 | [M-H]^-^ | 173.0455 | 0.062 | 0.119 | 0.095 | 1.94 | 1.54 | | 0.605 | | | ++++ | | ++++ | | ++++ | |
| Coniferyl alcohol | C10H12O3 | [M-H]^-^ | 179.0714 | 0.000 | 0.022 | 0.010 | - | - | | 0.275 | | | - | | ++ | | + | |
| Ferulic acid | C10H10O4 | [M-H]^-^ | 193.0506 | 0.007 | 0.051 | 0.019 | 7.49 | 2.81 | | 0.307 | | | + | | ++ | | ++ | |
| Sinapyl-alcohol | C11H14O4 | [M-H]^-^ | 209.0819 | 0.001 | 0.028 | 0.010 | 20.14 | 6.86 | | 0.084 | | | + | | ++ | | + | |
| UDP-Pentose | C14H22N2O16P2 | [M-H]^-^ | 535.0372 | 0.000 | 0.010 | 0.003 | - | - | | 0.466 | | | - | | + | | + | |
|  |  |  |  |  |  |  |  |  | |  | | |  | |  | |  | |
| **Cluster ions** |  |  |  |  |  |  |  |  | |  | | |  | |  | |  | |
| Glycerol+Hex | C9H20O9 | [M’-H]^-^ | 271.0350 | 0.403 | 0.172 | 0.157 | 0.43 | 0.39 | | 0.408 | | | ++++ | | +++ | | ++++ | |
| Glycerol+Hex | C9H20O9 | [M’+Cl]^-^ | 307.0802 | 0.000 | 0.000 | 0.001 | 1.05 | 2.44 | | 0.661 | | | + | | + | | + | |
| Shikimic acid+Hex | C13H22O11 | [M’-H]^-^ | 353.1089 | 0.058 | 0.122 | 0.162 | 2.12 | 2.81 | | 0.261 | | | +++ | | +++ | | ++++ | |
| Shikimic acid+Hex_2_ | C19H32O16 | [M’-H]^-^ | 515.1617 | 0.000 | 0.000 | 0.001 | 0.38 | 3.68 | | 0.229 | | | + | | + | | + | |
| Ferulic acid+Hex_2_ | C22H32O15 | [M’-H]^-^ | 535.1668 | 0.017 | 0.087 | 0.098 | 5.09 | 5.77 | | 0.131 | | | ++ | | +++ | | ++++ | |
| Sinapyl-alcohol+Hex | C17H26O10 | [M’-H]^-^ | 389.1453 | 0.000 | 0.001 | 0.002 | - | - | | 0.509 | | | - | | + | | + | |
| UDP-Pentose+Hex | C20H34N2O22P2 | [M’-H]^-^ | 715.1006 | 0.062 | 0.155 | 0.173 | 2.49 | 2.79 | | 0.215 | | | +++ | | ++++ | | +++ | |
|  |  |  |  |  |  |  |  |  | |  | | |  | |  | |  | |
| **Phytohormones and the precursors** | |  |  |  |  |  |  |  | |  | | |  | |  | |  | |
| 1-Aminocyclopropane-1-carboxylic acid | C4H7NO2 | [M-H]^-^ | 100.0404 | 0.002 | 0.006 | 0.016 | 2.69 | 6.86 | | 0.461 | | | + | | ++ | | + | |
| 1-Aminocyclopropane-1-carboxylic acid | C4H7NO2 | [M+Cl]^-^ | 136.0171 | 0.125 | 0.181 | 0.178 | 1.44 | 1.42 | | 0.594 | | | +++ | | ++++ | | ++++ | |
| Salicylic acid | C7H6O3 | [M-H]^-^ | 137.0244 | 0.075 | 0.205 | 0.164 | 2.71 | 2.18 | | 0.370 | | | +++ | | +++ | | ++++ | |
| 3-Indoleacetic Acid | C10H9NO2 | [M-H]^-^ | 174.0561 | 0.000 | 0.001 | 0.001 | 16.73 | 7.97 | | 0.388 | | | + | | + | | + | |
| Zeatin | C10H13N5O | [M-H]^-^ | 218.1047 | 0.001 | 0.000 | 0.004 | - | 6.44 | | 0.237 | | | + | | - | | ++ | |
| Abscisic acid | C15H20O4 | [M-H]^-^ | 263.1289 | 0.000 | 0.000 | 0.000 | 1.30 | 2.45 | | 0.738 | | | + | | + | | + | |
| Gibberellin | C19H22O6 | [M-H]^-^ | 345.1344 | 0.000 | 0.003 | 0.000 | 9.33 | 0.87 | | 0.417 | | | + | | + | | + | |
|  |  |  |  |  |  |  |  |  | |  | | |  | |  | |  | |
| **Cluster ions** |  |  |  |  |  |  |  |  | |  | | |  | |  | |  | |
| 1-Aminocyclopropane-1-carboxylic acid+Hex | C10H19NO8 | [M’-H]^-^ | 280.1038 | 0.010 | 0.017 | 0.002 | 1.73 | 0.16 | | 0.102 | | | + | | + | | + | |
| 1-Aminocyclopropane-1-carboxylic acid+Hex | C10H19NO8 | [M’+Cl]^-^ | 316.0805 | 0.001 | 0.005 | 0.007 | 4.44 | 5.61 | | 0.553 | | | + | | ++ | | + | |
| Salicylic acid+Hex | C13H18O9 | [M’-H]^-^ | 317.0878 | 0.025 | 0.090 | 0.109 | 3.57 | 4.35 | | 0.819 | | | ++ | | ++ | | ++ | |
| Salicylic acid+Hex | C13H18O9 | [M’+Cl]^-^ | 353.0645 | 0.000 | 0.001 | 0.001 | 14.65 | 12.36 | | 0.578 | | | + | | + | | + | |
| Salicylic acid+Hex_2_ | C19H28O14 | [M’-H]^-^ | 479.1406 | 0.012 | 0.033 | 0.012 | 2.74 | 0.98 | | 0.185 | | | ++ | | ++ | | ++ | |
| Gibberellin+Hex | C25H34O12 | [M’-H]^-^ | 525.1978 | 0.001 | 0.007 | 0.002 | 10.49 | 2.36 | | 0.369 | | | + | | + | | + | |
| Gibberellin+Hex_2_ | C31H44O17 | [M’-H]^-^ | 687.2506 | 0.000 | 0.000 | 0.000 | 2.52 | 1.55 | | 0.823 | | | + | | + | | + | |
|  |  |  |  |  |  |  |  |  | |  | | |  | |  | |  | |

| (*Continued from previous page*.) |  | |  |  | | |  | | |  | | |  | | | | | | |  | | |  |  | | | | | | |  | | | |  | |
| --- | --- | --- | --- | --- | --- | --- | --- | --- | --- | --- | --- | --- | --- | --- | --- | --- | --- | --- | --- | --- | --- | --- | --- | --- | --- | --- | --- | --- | --- | --- | --- | --- | --- | --- | --- | --- |
| Metabolites | Molecular  formula | | Ion type detected  [M=molecule]  [M’=cluster] ^&^ | | | Theoretical*  *m/z* | | | Relative abundance (%)† | | | | | | | | | | | | | *P* value | | |  | Frequency of detection‡ | | | | | | | | | | |
|  |  |  |  |  |  |  |  |  | N | | B | | | W | | B / N | | W / N | | | |  |  |  |  | N | | | | B | | | | W | | |
| **Organic acids** |  | |  | | |  | | |  | |  | | |  | |  | |  | | | |  | | |  | | | |  | | | |  | | | |
| Glyoxylic acid | C2H2O3 | | [M-H]^-^ | | | 72.9931 | | | 0.358 | | 0.595 | | | 0.493 | | 1.66 | | 1.38 | | | | 0.514 | | |  | ++++ | | | | ++++ | | | | ++++ | | |
| Glycolic acid | C2H4O3 | | [M-H]^-^ | | | 75.0088 | | | 0.368 | | 0.600 | | | 0.340 | | 1.63 | | 0.92 | | | | 0.626 | | |  | ++++ | | | | ++++ | | | | ++++ | | |
| Hydroxypyruvic acid | C3H4O4 | | [M-H]^-^ | | | 103.0037 | | | 1.156 | | 1.925 | | | 1.295 | | 1.66 | | 1.12 | | | | 0.635 | | |  | ++++ | | | | ++++ | | | | ++++ | | |
| Citramalic acid | C5H8O6 | | [M-H]^-^ | | | 147.0299 | | | 0.656 | | 0.991 | | | 0.907 | | 1.51 | | 1.38 | | | | 0.563 | | |  | +++ | | | | ++++ | | | | ++++ | | |
| Ascorbic acid | C6H8O6 | | [M-H]^-^ | | | 175.0247 | | | 0.738 | | 1.221 | | | 1.180 | | 1.66 | | 1.60 | | | | 0.706 | | |  | ++++ | | | | ++++ | | | | ++++ | | |
| Quinic acid | C7H12O6 | | [M-H]^-^ | | | 191.0561 | | | 3.953 | | 2.458 | | | 5.811 | | 0.62 | | 1.47 | | | | 0.390 | | |  | ++++ | | | | ++++ | | | | ++++ | | |
| Homocitric acid | C7H10O7 | | [M-H]^-^ | | | 205.0354 | | | 0.178 | | 0.233 | | | 0.163 | | 1.31 | | 0.91 | | | | 0.686 | | |  | ++++ | | | | ++++ | | | | ++++ | | |
| Chlorogenic acid | C16H18O9 | | [M-H]^-^ | | | 353.0875 | | | 0.366 | | 0.234 | | | 0.609 | | 0.64 | | 1.67 | | | | 0.517 | | |  | ++++ | | | | ++++ | | | | ++++ | | |
|  |  | |  | | |  | | |  | |  | | |  | |  | |  | | | |  | | |  |  | | | |  | | | |  | | |
| **Cluster ions** |  | |  | | |  | | |  | |  | | |  | |  | |  | | | |  | | |  |  | | | |  | | | |  | | |
| Glyoxylic acid+Hex | C8H14O9 | | [M’-H]^-^ | | | 253.0565 | | | 0.310 | | 0.248 | | | 0.173 | | 0.80 | | 0.56 | | | | 0.690 | | |  | +++ | | | | ++++ | | | | ++++ | | |
| Glycolic acid+Hex | C8H16O9 | | [M’-H]^-^ | | | 255.0722 | | | 0.799 | | 0.391 | | | 0.266 | | 0.49 | | 0.33 | | | | 0.298 | | |  | ++++ | | | | ++++ | | | | ++++ | | |
| Glycolic acid+Hex_2_ | C14H26O14 | | [M’-H]^-^ | | | 417.1250 | | | 0.090 | | 0.055 | | | 0.047 | | 0.61 | | 0.52 | | | | 0.431 | | |  | +++ | | | | +++ | | | | ++++ | | |
| Hydroxypyruvic acid+Hex | C9H16O10 | | [M’-H]^-^ | | | 283.0671 | | | 0.091 | | 0.089 | | | 0.090 | | 0.98 | | 0.98 | | | | 0.999 | | |  | +++ | | | | +++ | | | | ++++ | | |
| Hydroxypyruvic acid+Hex_2_ | C15H26O15 | | [M’-H]^-^ | | | 445.1199 | | | 0.049 | | 0.036 | | | 0.042 | | 0.73 | | 0.85 | | | | 0.790 | | |  | +++ | | | | +++ | | | | +++ | | |
| Citramalic acid+Hex | C11H20O12 | | [M’-H]^-^ | | | 327.0933 | | | 0.125 | | 0.477 | | | 1.182 | | 3.82 | | 9.46 | | | | 0.229 | | |  | +++ | | | | ++++ | | | | ++++ | | |
| Citramalic acid+Hex_2_ | C17H30O17 | | [M’-H]^-^ | | | 489.1461 | | | 0.008 | | 0.002 | | | 0.003 | | 0.27 | | 0.35 | | | | 0.221 | | |  | ++ | | | | + | | | | + | | |
| Ascorbic acid+Hex | C12H20O12 | | [M’-H]^-^ | | | 355.0882 | | | 0.166 | | 0.077 | | | 0.074 | | 0.46 | | 0.44 | | | | 0.199 | | |  | +++ | | | | ++++ | | | | ++++ | | |
| Quinic acid+Hex | C13H24O12 | | [M’-H]^-^ | | | 371.1195 | | | 0.414 | | 0.150 | | | 0.242 | | 0.36 | | 0.58 | | | | 0.182 | | |  | ++++ | | | | ++++ | | | | ++++ | | |
| Quinic acid+Hex_2_ | C19H34O17 | | [M’-H]^-^ | | | 533.1723 | | | 0.132 | | 0.061 | | | 0.118 | | 0.46 | | 0.89 | | | | 0.293 | | |  | ++++ | | | | +++ | | | | ++++ | | |
| Chlorogenic acid+Hex | C22H30O15 | | [M’-H]^-^ | | | 533.1509 | | | 0.022 | | 0.021 | | | 0.030 | | 0.94 | | 1.35 | | | | 0.737 | | |  | +++ | | | | ++ | | | | +++ | | |
| Chlorogenic acid+Hex_2_ | C28H40O20 | | [M’-H]^-^ | | | 695.2037 | | | 0.027 | | 0.025 | | | 0.024 | | 0.93 | | 0.90 | | | | 0.983 | | |  | ++ | | | | ++ | | | | +++ | | |
|  | |  | | |  | | |  | | | |  | | |  | |  | |  | |  | | | |  | |  |  | | | |  | | | |  |
| * All the theoretical values are quoted from Metlin (http://metlin.scripps.edu/index.php).  † Values were calculated as a percentage to the base peak. Different letters indicate a significant difference (Tukey-Kramer test, p < 0.05)  ‡ Signal detected (+); Signal not detected (-). Frequencies of detection (x) in 4 individual measurements are indicated as: ++++; 75%≦ x < 100%, +++; 50%≦ x < 75%, ++; 25%≦ x < 50%, +; 0%< x <25%.  ^&^ These clusters are the molecular aggregates stably formed in solution and in gas state because the intermolecular interaction among the molecular unities is quite strong (e.g., hydrogen bridge); its formation depends on the concentration of the molecular constituent in the analysed solution; at higher concentrations, the chance to obtain cluster signals in electrospray ionization mass spectrometry is higher. PicoPPESI mass spectrometry is an electrospray ionization mass spectrometry technique. | | | | | | | | | | | | | | | | | | | | | | | | | | | | | | | | | | | | |

**Table S2. Metabolites detected in each of mesocarp cells located at the outer parenchyma (N) and two regions corresponding to the border (N(B)) and watercore (N(W)) in the normal apple fruit using picoPPESI-MS in negative ion mode.**

| **Metabolites detected in each of mesocarp cells located at the outer parenchyma (N) and two regions corresponding to the border (N(B)) and watercore (N(W)) in the normal apple fruit using picoPPESI-MS in negative ion mode.** | | | | | | | | | | | | | | | | | | | | | | | | | | | | | | |
| --- | --- | --- | --- | --- | --- | --- | --- | --- | --- | --- | --- | --- | --- | --- | --- | --- | --- | --- | --- | --- | --- | --- | --- | --- | --- | --- | --- | --- | --- | --- |
| Metabolites | | Molecular  formula | | Ion type detected  [M=molecule]  [M’=cluster] ^&^ | | Theoretical*  *m/z* | | | Relative abundance (%)† | | | | | | | | | | | | | *P* value | | Frequency of detection‡ | | | | | | |
|  |  |  |  |  |  |  |  |  | N | | N(B) | | | N(W) | | | N(B) / N | | | N(W) / N | |  |  | N | | | N(B) | | | N(W) |
| **Metabolites in glycolysis / fermentation** | | | |  | |  | |  | | | | |  | |  | | |  | | |  | |  | | |  | | |  | |
| Pyruvic acid | | C3H4O3 | | [M-H]^-^ | | 87.0088 | | | 0.896 | | 0.708 | | | 0.615 | | | 0.79 | | | 0.69 | | 0.900 | | ++++ | | | ++++ | | | ++++ |
| Lactic acid | | C3H6O3 | | [M-H]^-^ | | 89.0244 | | | 4.309 | | 3.963 | | | 2.879 | | | 0.92 | | | 0.67 | | 0.867 | | ++++ | | | ++++ | | | ++++ |
| Hex | | C6H12O6 | | [M-H]^-^ | | 179.0561 | | | 32.322 | | 32.839 | | | 20.807 | | | 1.02 | | | 0.64 | | 0.610 | | ++++ | | | ++++ | | | ++++ |
| Hex | | C6H12O6 | | [M+Cl]^-^ | | 215.0328 | | | 1.434 | | 2.006 | | | 8.404 | | | 1.40 | | | 5.86 | | 0.395 | | ++++ | | | ++++ | | | ++++ |
| HexP | | C6H13O9P | | [M-H]^-^ | | 259.0224 | | | 0.094 | | 0.049 | | | 0.026 | | | 0.53 | | | 0.28 | | 0.400 | | +++ | | | ++++ | | | ++ |
| Hex_2_ | | C12H22O11 | | [M-H]^-^ | | 341.1089 | | | 0.846 | | 1.190 | | | 1.551 | | | 1.41 | | | 1.83 | | 0.467 | | ++++ | | | ++++ | | | ++++ |
| Hex_2_ | | C12H22O11 | | [M+Cl]^-^ | | 377.0856 | | | 0.361 | | 0.326 | | | 1.070 | | | 0.90 | | | 2.96 | | 0.532 | | +++ | | | ++++ | | | +++ |
| UDP-Hex | | C15H24N2O17P2 | | [M-H]^-^ | | 565.0477 | | | 0.000 | | 0.004 | | | 0.011 | | | 11.29 | | | 34.88 | | 0.500 | | + | | | + | | | + |
|  | |  | |  | |  | | |  | |  | | |  | | |  | | |  | |  | |  | | |  | | |  |
| Phosphorous acid | | H3O3P | | [M-H]^-^ | | 78.9591 | | | 0.184 | | 0.015 | | | 0.002 | | | 0.08 | | | 0.01 | | 0.235 | | +++ | | | +++ | | | + |
| Phosphoric acid | | H3O4P | | [M-H]^-^ | | 96.9696 | | | 0.386 | | 0.137 | | | 0.064 | | | 0.35 | | | 0.16 | | 0.243 | | +++ | | | ++++ | | | +++ |
| Uridine 5'-diphosphate (UDP) | | C9H14N2O12P2 | | [M-H]^-^ | | 402.9949 | | | 0.042 | | 0.009 | | | 0.032 | | | 0.22 | | | 0.76 | | 0.228 | | +++ | | | ++ | | | +++ |
|  | |  | |  | |  | | |  | |  | | |  | | |  | | |  | |  | |  | | |  | | |  |
| **Cluster ions** | |  | |  | |  | | |  | |  | | |  | | |  | | |  | |  | |  | | |  | | |  |
| Pyruvic acid+Hex | | C9H10O9 | | [M’-H]^-^ | | 267.0722 | | | 0.081 | | 0.127 | | | 0.053 | | | 1.58 | | | 0.66 | | 0.434 | | ++++ | | | ++++ | | | +++ |
| Pyruvic acid+Hex_2_ | | C15H26O14 | | [M’-H]^-^ | | 429.1250 | | | 0.011 | | 0.001 | | | 0.005 | | | 0.08 | | | 0.46 | | 0.237 | | ++ | | | - | | | ++ |
| Pyruvic acid+Hex_2_ | | C15H26O14 | | [M’+Cl]^-^ | | 465.1017 | | | 0.124 | | 0.357 | | | 0.196 | | | 2.87 | | | 1.58 | | 0.294 | | ++++ | | | ++++ | | | ++++ |
| Lactic acid+Hex | | C9H18O9 | | [M’-H]^-^ | | 269.0878 | | | 0.174 | | 1.301 | | | 0.337 | | | 7.47 | | | 1.94 | | 0.353 | | ++++ | | | ++++ | | | ++++ |
| Lactic acid+Hex_2_ | | C15H28O14 | | [M’-H]^-^ | | 431.1406 | | | 0.009 | | 0.070 | | | 0.029 | | | 7.87 | | | 3.30 | | 0.473 | | ++ | | | ++ | | | ++ |
| Glyceric acid+Hex | | C9H18O10 | | [M’-H]^-^ | | 285.0827 | | | 0.025 | | 0.002 | | | 0.001 | | | 0.06 | | | 0.06 | | 0.180 | | + | | | + | | | + |
| Glyceric acid+Hex_2_ | | C15H28O15 | | [M’+Cl]^-^ | | 447.1355 | | | 0.017 | | 0.015 | | | 0.041 | | | 0.87 | | | 2.37 | | 0.643 | | +++ | | | +++ | | | ++ |
| (Hex)_2_ | | C12H24O12 | | [M’-H]^-^ | | 359.1195 | | | 2.737 | | 2.950 | | | 0.988 | | | 1.08 | | | 0.36 | | 0.323 | | ++++ | | | ++++ | | | ++++ |
| (Hex)_2_ | | C12H24O12 | | [M’-H]^-^ | | 395.0962 | | | 0.000 | | 0.000 | | | 0.028 | | | - | | | - | | 0.265 | | - | | | - | | | + |
| Hex+Hex_2_ | | C18H34O17 | | [M’-H]^-^ | | 521.1723 | | | 0.165 | | 0.500 | | | 0.804 | | | 3.03 | | | 4.88 | | 0.164 | | +++ | | | ++++ | | | ++++ |
| (Hex)_3_ | | C18H36O18 | | [M’-H]^-^ | | 539.1828 | | | 0.000 | | 0.002 | | | 0.003 | | | - | | | - | | 0.477 | | - | | | + | | | + |
| (Hex)_2_+Hex_2_ | | C24H46O23 | | [M’-H]^-^ | | 701.2357 | | | 0.000 | | 0.004 | | | 0.004 | | | - | | | - | | 0.299 | | - | | | ++ | | | ++ |
| Hex+Hex_4_  Hex_2_+Hex_3_ | | C30H54O25 | | [M’-H]^-^ | | 845.2780 | | | 0.000 | | 0.000 | | | 0.006 | | | - | | | 12.75 | | 0.392 | | + | | | - | | | + |
| Hex+(Hex_2_)_2_  (Hex)_2_+Hex_3_ | | C30H54O27 | | [M’-H]^-^ | | 863.2885 | | | 0.000 | | 0.006 | | | 0.002 | | | - | | | - | | 0.372 | | - | | | + | | | + |
| Hex+Hex_2_+Hex_3_  (Hex)_2_+Hex_4_  (Hex_2_)_3_ | | C36H66O33 | | [M’-H]^-^ | | 1025.3413 | | | 0.002 | | 0.000 | | | 0.012 | | | - | | | 5.99 | | 0.273 | | + | | | - | | | ++ |
| (Hex)_2_+(Hex_2_)_2_ | | C36H66O33 | | [M’-H]^-^ | | 1043.3519 | | | 0.013 | | 0.601 | | | 0.392 | | | 45.69 | | | 29.80 | | 0.411 | | + | | | ++ | | | + |
| HexP+Hex | | C12H25O15P | | [M’-H]^-^ | | 439.0856 | | | 0.683 | | 1.198 | | | 1.246 | | | 1.75 | | | 1.83 | | 0.377 | | ++++ | | | ++++ | | | ++++ |
| HexP+Hex_2_ | | C18H35O20P | | [M’+Cl]^-^ | | 601.1386 | | | 0.042 | | 0.036 | | | 0.028 | | | 0.86 | | | 0.68 | | 0.866 | | +++ | | | +++ | | | +++ |
| (Hex_2_)_2_  Hex+Hex_3_ | | C24H44O22 | |  | | 683.2252 | | | 0.193 | | 0.502 | | | 0.467 | | | 2.61 | | | 2.42 | | 0.304 | | ++ | | | ++++ | | | +++ |
| (Hex_2_)_2_  Hex+Hex_3_ | | C24H44O22 | | [M’-H]^-^ | | 719.2018 | | | 0.006 | | 0.005 | | | 0.072 | | | 0.72 | | | 11.19 | | 0.459 | | + | | | + | | | + |
|  | |  | | [M’-H]^-^ | |  | | |  | |  | | |  | | |  | | |  | |  | |  | | |  | | |  |
| H_3_PO_4_+Hex | | C6H14O10P | | [M’-H]^-^ | | 277.0330 | | | 0.426 | | 1.072 | | | 0.708 | | | 2.52 | | | 1.66 | | 0.155 | | ++++ | | | ++++ | | | ++++ |
| UDP+Hex | | C15H26N2O18P2 | | [M’-H]^-^ | | 583.0583 | | | 0.000 | | 0.000 | | | 0.004 | | | - | | | - | | 0.150 | | - | | | - | | | + |
|  |  | |  | |  | |  | | |  | |  | | | |  | | |  | |  | |  | |  | | |  | | |

| (*Continued from previous page*.) | | | |  | |  | |  | | |  | | | |  | | | | |  | |  | | |  | | |  | | | | | | | |  |
| --- | --- | --- | --- | --- | --- | --- | --- | --- | --- | --- | --- | --- | --- | --- | --- | --- | --- | --- | --- | --- | --- | --- | --- | --- | --- | --- | --- | --- | --- | --- | --- | --- | --- | --- | --- | --- |
| Metabolites | | | | Molecular  formula | | Ion type detected  [M=molecule]  [M’=cluster] ^&^ | | Theoretical*  *m/z* | | Relative abundance (%)† | | | | | | | | | | | *P* value | | | | | Frequency of detection‡ | | | | | | | | | | |
|  |  |  |  |  |  |  |  |  |  | N | | N(B) | | N(W) | | | N(B) / N | | N(W) / N | |  |  |  |  |  | N | | | | N(B) | | | | | N(W) | |
| **Metabolites in TCA cycle** | | | |  | |  | |  | |  | |  | |  | | |  | |  | |  | | | | | | | |  | | | | | | | |
| Fumaric acid | | | | C4H4O4 | | [M-H]^-^ | | 115.0037 | | 11.393 | | 10.403 | | 9.488 | | | 0.91 | | 0.83 | | 0.704 | | | | |  | ++++ | | | | ++++ | | | ++++ | | |
| Succinic acid | | | | C4H6O4 | | [M-H]^-^ | | 117.0193 | | 0.893 | | 0.634 | | 0.298 | | | 0.71 | | 0.33 | | 0.346 | | | | |  | ++++ | | | | ++++ | | | ++++ | | |
| Malic acid | | | | C4H6O5 | | [M-H]^-^ | | 133.0142 | | 97.528 | | 96.082 | | 92.733 | | | 0.99 | | 0.95 | | 0.777 | | | | |  | ++++ | | | | ++++ | | | ++++ | | |
| α-ketoglutaric acid | | | | C5H6O5 | | [M-H]^-^ | | 145.0142 | | 1.084 | | 0.230 | | 0.261 | | | 0.21 | | 0.24 | | 0.128 | | | | |  | +++ | | | | +++ | | | +++ | | |
| *cis*-Aconitic acid | | | | C6H6O6 | | [M-H]^-^ | | 173.0092 | | 0.947 | | 0.144 | | 0.411 | | | 0.15 | | 0.43 | | 0.259 | | | | |  | +++ | | | | ++++ | | | ++++ | | |
| Citric acid  Isocitric acid | | | | C6H8O7 | | [M-H]^-^ | | 191.0197 | | 1.474 | | 0.829 | | 1.063 | | | 0.56 | | 0.72 | | 0.369 | | | | |  | ++++ | | | | ++++ | | | ++++ | | |
|  | | | |  | |  | |  | |  | |  | |  | | |  | |  | |  | | | | |  |  | | | |  | | |  | | |
| **Cluster ions** | | | |  | |  | |  | |  | |  | |  | | |  | |  | |  | | | | |  |  | | | |  | | |  | | |
| Fumaric acid+Hex | | | | C10H16O10 | | [M’-H]^-^ | | 295.0671 | | 0.227 | | 0.189 | | 0.220 | | | 0.83 | | 0.97 | | 0.872 | | | | |  | ++++ | | | | ++++ | | | ++++ | | |
| Fumaric acid+Hex_2_ | | | | C16H26O15 | | [M’-H]^-^ | | 457.1199 | | 0.087 | | 0.025 | | 0.070 | | | 0.28 | | 0.80 | | 0.584 | | | | |  | +++ | | | | ++++ | | | +++ | | |
| Succinic acid+Hex | | | | C10H18O10 | | [M’-H]^-^ | | 297.0827 | | 0.009 | | 0.011 | | 0.009 | | | 1.25 | | 0.96 | | 0.891 | | | | |  | ++ | | | | +++ | | | ++ | | |
| Succinic acid+Hex | | | | C10H18O10 | | [M’-H]^-^ | | 333.0594 | | 0.096 | | 0.146 | | 0.281 | | | 1.52 | | 2.92 | | 0.407 | | | | |  | ++++ | | | | ++++ | | | +++ | | |
| Succinic acid+Hex_2_ | | | | C16H28O15 | | [M’+Cl]^-^ | | 459.1355 | | 0.001 | | 0.008 | | 0.003 | | | 7.60 | | 2.44 | | 0.289 | | | | |  | + | | | | ++ | | | + | | |
| Malic acid+Hex | | | | C10H18O11 | | [M’-H]^-^ | | 313.0776 | | 2.848 | | 2.515 | | 3.793 | | | 0.88 | | 1.33 | | 0.724 | | | | |  | ++++ | | | | ++++ | | | ++++ | | |
| Malic acid+Hex_2_ | | | | C18H30O17 | | [M’-H]^-^ | | 475.1304 | | 0.581 | | 0.746 | | 1.214 | | | 1.28 | | 2.09 | | 0.202 | | | | |  | ++++ | | | | ++++ | | | +++ | | |
| Malic acid+Hex_3_ | | | | C22H38O21 | | [M’-H]^-^ | | 637.1833 | | 0.021 | | 0.012 | | 0.024 | | | 0.56 | | 1.17 | | 0.657 | | | | |  | ++ | | | | ++ | | | +++ | | |
| Malic acid+Hex+Hex_3_  Malic acid+(Hex_2_)_2_ | | | | C30H52O26 | | [M’-H]^-^ | | 817.2467 | | 0.001 | | 0.002 | | 0.012 | | | 2.62 | | 13.13 | | 0.266 | | | | |  | + | | | | + | | | ++ | | |
| α-ketoglutaric acid+Hex | | | | C11H18O11 | | [M’-H]^-^ | | 325.0776 | | 0.078 | | 0.032 | | 0.076 | | | 0.41 | | 0.98 | | 0.392 | | | | |  | +++ | | | | ++++ | | | +++ | | |
| *cis*-Aconitic acid +Hex | | | | C12H18O12 | | [M’-H]^-^ | | 353.0726 | | 0.040 | | 0.009 | | 0.020 | | | 0.22 | | 0.49 | | 0.353 | | | | |  | ++ | | | | ++ | | | ++ | | |
|  | | | |  | |  | |  | |  | |  | |  | | |  | |  | |  | | | | |  |  | | | |  | | |  | | |
| **Amino acids** | | | |  | |  | |  | |  | |  | |  | | |  | |  | |  | | | | |  |  | | | |  | | |  | | |
| Alanine | | | | C3H7NO2 | | [M-H]^-^ | | 88.0404 | | 0.001 | | 0.001 | | 0.002 | | | 0.95 | | 2.31 | | 0.674 | | | | |  | + | | | | + | | | + | | |
| Serine | | | | C3H7NO3 | | [M-H]^-^ | | 104.0353 | | 0.004 | | 0.011 | | 0.012 | | | 2.57 | | 2.82 | | 0.606 | | | | |  | ++ | | | | ++ | | | ++ | | |
| Oxoproline | | | | C5H7NO3 | | [M-H]^-^ | | 128.0353 | | 0.013 | | 0.013 | | 0.013 | | | 0.98 | | 1.01 | | 1.000 | | | | |  | ++ | | | | ++ | | | ++ | | |
| Asparagine | | | | C4H8N2O3 | | [M-H]^-^ | | 131.0462 | | 0.007 | | 0.053 | | 0.050 | | | 8.03 | | 7.60 | | 0.452 | | | | |  | ++ | | | | ++ | | | +++ | | |
| Aspartic acid | | | | C4H7NO4 | | [M-H]^-^ | | 132.0302 | | 0.074 | | 0.399 | | 0.875 | | | 5.40 | | 11.84 | | 0.097 | | | | |  | ++++ | | | | ++++ | | | ++++ | | |
| Glutamine | | | | C5H10N2O3 | | [M-H]^-^ | | 145.0619 | | 0.001 | | 0.000 | | 0.006 | | | - | | 4.38 | | 0.230 | | | | |  | + | | | | - | | | ++ | | |
| Glutamic acid | | | | C5H9NO4 | | [M-H]^-^ | | 146.0459 | | 0.003 | | 0.031 | | 0.094 | | | 11.78 | | 35.08 | | 0.250 | | | | |  | ++ | | | | +++ | | | +++ | | |
| Tryptophan | | | | C11H12N2O2 | | [M-H]^-^ | | 203.0826 | | 1.904 | | 0.003 | | 0.009 | | | 0.00 | | 0.00 | | 0.346 | | | | |  | ++ | | | | + | | | ++ | | |
|  | | | |  | |  | |  | |  | |  | |  | | |  | |  | |  | | | | |  |  | | | |  | | |  | | |
| **Cluster ions** | | | |  | |  | |  | |  | |  | |  | | |  | |  | |  | | | | |  |  | | | |  | | |  | | |
| Alanine+Hex | | | | C9H20NO8 | | [M’-H]^-^ | | 268.1038 | | 0.000 | | 0.002 | | 0.009 | | | - | | - | | 0.377 | | | | |  | - | | | | + | | | + | | |
| Alanine+Hex_2_ | | | | C15H29NO13 | | [M’-H]^-^ | | 430.1566 | | 0.000 | | 0.000 | | 0.009 | | | - | | - | | 0.166 | | | | |  | - | | | | - | | | ++ | | |
| Serine+Hex | | | | C9H19NO9 | | [M’-H]^-^ | | 284.0987 | | 0.005 | | 0.006 | | 0.016 | | | 1.41 | | 3.58 | | 0.463 | | | | |  | + | | | | + | | | ++ | | |
| Serine+Hex_2_ | | | | C15H29NO13 | | [M’-H]^-^ | | 446.1515 | | 0.000 | | 0.008 | | 0.023 | | | - | | - | | 0.212 | | | | |  | - | | | | + | | | ++ | | |
| Proline+Hex | | | | C11H21NO8 | | [M’-H]^-^ | | 294.1194 | | 0.000 | | 0.000 | | 0.001 | | | - | | - | | 0.304 | | | | |  | - | | | | + | | | + | | |
| Oxoproline+Hex | | | | C11H19NO9 | | [M’-H]^-^ | | 308.0987 | | 0.000 | | 0.003 | | 0.005 | | | - | | - | | 0.279 | | | | |  | - | | | | + | | | ++ | | |
| Oxoproline+Hex_2_ | | | | C17H29NO14 | | [M’-H]^-^ | | 470.1515 | | 0.000 | | 0.005 | | 0.003 | | | - | | - | | 0.471 | | | | |  | - | | | | + | | | + | | |
| Leucine+Hex  Isoleucine+Hex | | | | C12H23NO8 | | [M’-H]^-^ | | 310.1507 | | 0.000 | | 0.003 | | 0.005 | | | - | | - | | 0.503 | | | | |  | - | | | | + | | | + | | |
| Asparagine+Hex | | | | C10H20N2O9 | | [M’-H]^-^ | | 311.1096 | | 0.000 | | 0.050 | | 0.059 | | | - | | - | | 0.277 | | | | |  | - | | | | ++ | | | ++ | | |
| Asparagine+Hex_2_ | | | | C16H30N2O14 | | [M’-H]^-^ | | 473.1624 | | 0.001 | | 0.112 | | 0.146 | | | 200.79 | | 261.35 | | 0.194 | | | | |  | + | | | | +++ | | | ++ | | |
| Aspartic acid+Hex | | | | C10H19NO10 | | [M’-H]^-^ | | 312.0936 | | 0.004 | | 0.055 | | 0.087 | | | 14.42 | | 22.93 | | 0.105 | | | | |  | ++ | | | | ++ | | | +++ | | |
| Aspartic acid+Hex_2_ | | | | C16H29NO15 | | [M’-H]^-^ | | 474.1464 | | 0.060 | | 0.167 | | 0.426 | | | 2.80 | | 7.14 | | 0.117 | | | | |  | +++ | | | | ++++ | | | +++ | | |
| Aspartic acid+(Hex)_2_ | | | | C16H31NO16 | | [M’-H]^-^ | | 492.1570 | | 0.000 | | 0.000 | | 0.002 | | | - | | - | | 0.403 | | | | |  | - | | | | - | | | + | | |
| Aspartic acid+Hex+Hex_2_ | | | | C22H41NO21 | | [M’-H]^-^ | | 654.2098 | | 0.000 | | 0.000 | | 0.001 | | | - | | - | | 0.403 | | | | |  | - | | | | - | | | + | | |
| Aspartic acid+(Hex_2_)_2_  Aspartic acid+Hex+Hex_3_ | | | | C28H52O24 | | [M’-H]^-^ | | 816.2627 | | 0.000 | | 0.003 | | 0.015 | | | - | | - | | 0.373 | | | | |  | - | | | | + | | | ++ | | |
| Glutamic acid+Hex | | | | C11H21NO10 | | [M’-H]^-^ | | 326.1093 | | 0.000 | | 0.005 | | 0.006 | | | - | | - | | 0.499 | | | | |  | - | | | | ++ | | | + | | |
| Glutamic acid+Hex_2_ | | | | C17H31NO15 | | [M’-H]^-^ | | 488.1621 | | 0.002 | | 0.029 | | 0.025 | | | 17.24 | | 15.05 | | 0.184 | | | | |  | + | | | | ++ | | | ++ | | |
|  |  |  |  | |  | |  | |  | | | |  | | |  | |  | | | | |  |  | | | | | | | |  |  | | | |

| (*Continued from previous page*.) | | | | | | |  |  |  | | |  | | |  | | | | | | | | | | | | | |  | | |  | | |  | | | | |
| --- | --- | --- | --- | --- | --- | --- | --- | --- | --- | --- | --- | --- | --- | --- | --- | --- | --- | --- | --- | --- | --- | --- | --- | --- | --- | --- | --- | --- | --- | --- | --- | --- | --- | --- | --- | --- | --- | --- | --- |
| Metabolites | | | | | | | Molecular  formula | | | Ion type detected  [M=molecule]  [M’=cluster] ^&^ | | | Theoretical*  *m/z* | | | Relative abundance (%)† | | | | | | | | | *P* value | | Frequency of detection‡ | | | | | | | | | | | | |
|  |  |  |  |  |  |  |  |  |  |  |  |  |  |  |  | N | | N(B) | N(W) | | | | N(B) / N | N(W) / N |  |  | N | | | | | | N(B) | | | N(W) | | | |
| **Saccharides** | | | | | | |  | | |  | | |  | | |  | |  |  | | | |  |  |  | | |  | | |  | | | | | | | |  |
|  | | | | | | |  | | |  | | |  | | |  | |  |  | | | |  |  |  | | |  | |  | | | | | | |  | | |
| **Monosaccharide** | | | | | | |  | | |  | | |  | | |  | |  |  | | | |  |  |  | | |  |  | | | | |  | | | |  | |
| Tetrose | | | | | | | C4H8O4 | | | [M-H]^-^ | | | 119.0350 | | | 1.695 | | 1.811 | 1.010 | | | | 1.07 | 0.60 | 0.697 | | |  | ++++ | | | | | ++++ | | | | ++++ | |
| Pentose | | | | | | | C5H10O5 | | | [M-H]^-^ | | | 149.0455 | | | 0.746 | | 0.710 | 0.442 | | | | 0.95 | 0.59 | 0.649 | | |  | ++++ | | | | | ++++ | | | | ++++ | |
| Heptose | | | | | | | C7H14O7 | | | [M-H]^-^ | | | 209.0667 | | | 0.206 | | 0.064 | 0.231 | | | | 0.31 | 1.12 | 0.356 | | |  | ++++ | | | | | ++++ | | | | ++++ | |
| Octose | | | | | | | C8H16O8 | | | [M-H]^-^ | | | 239.0772 | | | 0.340 | | 0.368 | 0.256 | | | | 1.08 | 0.75 | 0.766 | | |  | ++++ | | | | | ++++ | | | | ++++ | |
|  | | | | | | |  | | |  | | |  | | |  | |  |  | | | |  |  |  | | |  |  | | | | |  | | | |  | |
| **Cluster ions** | | | | | | |  | | |  | | |  | | |  | |  |  | | | |  |  |  | | |  |  | | | | |  | | | |  | |
| Tetrose+Hex | | | | | | | C10H20O10 | | | [M’-H]^-^ | | | 299.0984 | | | 0.000 | | 0.020 | 0.283 | | | | - | - | 0.336 | | |  | - | | | | | + | | | | +++ | |
| Tetrose+Hex | | | | | | | C10H20O10 | | | [M’+Cl]^-^ | | | 335.0751 | | | 0.005 | | 0.007 | 0.024 | | | | 1.44 | 5.04 | 0.203 | | |  | ++ | | | | | ++ | | | | +++ | |
| Tetrose+Hex_2_ | | | | | | | C16H30O15 | | | [M’-H]^-^ | | | 461.1512 | | | 0.468 | | 0.130 | 0.126 | | | | 0.28 | 0.27 | 0.436 | | |  | +++ | | | | | +++ | | | | ++ | |
| Pentose+Hex | | | | | | | C11H22O11 | | | [M’-H]^-^ | | | 329.1089 | | | 0.012 | | 0.003 | 0.007 | | | | 0.21 | 0.58 | 0.435 | | |  | ++ | | | | | + | | | | ++ | |
| Pentose+Hex_2_ | | | | | | | C17H32O16 | | | [M’-H]^-^ | | | 491.1617 | | | 0.006 | | 0.000 | 0.007 | | | | - | 1.23 | 0.439 | | |  | + | | | | | - | | | | ++ | |
| Heptose+Hex | | | | | | | C13H26O13 | | | [M’-H]^-^ | | | 389.1301 | | | 0.021 | | 0.015 | 0.008 | | | | 0.72 | 0.36 | 0.542 | | |  | ++ | | | | | ++ | | | | ++ | |
| Heptose+Hex_2_ | | | | | | | C19H36O18 | | | [M’-H]^-^ | | | 551.1829 | | | 0.000 | | 0.000 | 0.006 | | | | - | - | 0.178 | | |  | - | | | | | - | | | | ++ | |
| Octose+Hex | | | | | | | C14H28O14 | | | [M’-H]^-^ | | | 419.1406 | | | 0.000 | | 0.000 | 0.000 | | | | - | - | 0.403 | | |  | - | | | | | - | | | | + | |
| Octose+Hex_2_ | | | | | | | C20H28O19 | | | [M’-H]^-^ | | | 581.1934 | | | 0.000 | | 0.000 | 0.002 | | | | - | - | 0.143 | | |  | - | | | | | - | | | | + | |
| Octose+Hex_3_ | | | | | | | C26H48O24 | | | [M’-H]^-^ | | | 743.2463 | | | 0.000 | | 0.000 | 0.001 | | | | - | - | 0.403 | | |  | - | | | | | - | | | | + | |
|  | | | | | | |  | | |  | | |  | | |  | |  |  | | | |  |  |  | | |  |  | | | | |  | | | |  | |
| **Oligosaccharide** | | | | | | |  | | |  | | |  | | |  | |  |  | | | |  |  |  | | |  |  | | | | |  | | | |  | |
| Hex_3_ | | | | | | | C18H32O16 | | | [M-H]^-^ | | | 503.1618 | | | 0.017 | | 0.032 | 0.048 | | | | 1.83 | 2.75 | 0.348 | | |  | ++ | | | | | ++++ | | | | +++ | |
|  | | | | | | |  | | |  | | |  | | |  | |  |  | | | |  |  |  | | |  |  | | | | |  | | | |  | |
| **Sugar alcohols** | | | | | | |  | | |  | | |  | | |  | |  |  | | | |  |  |  | | |  |  | | | | |  | | | |  | |
| Hexitol | | | | | | | C6H14O6 | | | [M-H]^-^ | | | 181.0718 | | | 4.280 | | 5.327 | 3.049 | | | | 1.24 | 0.71 | 0.581 | | |  | ++++ | | | | | ++++ | | | | ++++ | |
| Hexitol | | | | | | | C6H14O6 | | | [M+Cl]^-^ | | | 217.0484 | | | 0.101 | | 0.153 | 0.389 | | | | 1.51 | 3.84 | 0.437 | | |  | +++ | | | | | +++ | | | | +++ | |
|  | | | | | | |  | | |  | | |  | | |  | |  |  | | | |  |  |  | | |  |  | | | | |  | | | |  | |
| **Cluster ions** | | | | | | |  | | |  | | |  | | |  | |  |  | | | |  |  |  | | |  |  | | | | |  | | | |  | |
| Hexitol+Hex | | | | | | | C12H26O12 | | | [M’-H]^-^ | | | 361.1352 | | | 0.150 | | 0.213 | 0.178 | | | | 1.43 | 1.19 | 0.813 | | |  | ++++ | | | | | ++++ | | | | ++++ | |
| Hexitol+Hex_2_ | | | | | | | C18H36O17 | | | [M’-H]^-^ | | | 523.1880 | | | 0.009 | | 0.024 | 0.085 | | | | 2.51 | 9.07 | 0.267 | | |  | ++ | | | | | ++ | | | | ++ | |
| Hexitol+Hex_3_ | | | | | | | C24H46O22 | | | [M’-H]^-^ | | | 685.2409 | | | 0.000 | | 0.000 | 0.000 | | | | - | - | 1.000 | | |  | - | | | | | - | | | | - | |
|  | | | | | | |  | | |  | | |  | | |  | |  |  | | | |  |  |  | | |  |  | | | | |  | | | |  | |
| **Deoxy sugar** | | | | | | |  | | |  | | |  | | |  | |  |  | | | |  |  |  | | |  |  | | | | |  | | | |  | |
| Deoxyhexose | | | | | | | C6H12O5 | | | [M-H]^-^ | | | 163.0612 | | | 12.324 | | 8.521 | 13.122 | | | | 0.69 | 1.06 | 0.747 | | |  | ++++ | | | | | ++++ | | | | ++++ | |
|  | | | | | | |  | | |  | | |  | | |  | |  |  | | | |  |  |  | | |  |  | | | | |  | | | |  | |
| **Cluster ions** | | | | | | |  | | |  | | |  | | |  | |  |  | | | |  |  |  | | |  |  | | | | |  | | | |  | |
| Deoxyhexose+Hex | | | | | | | C12H24O11 | | | [M’-H]^-^ | | | 343.1246 | | | 0.013 | | 0.010 | 0.094 | | | | 0.81 | 7.40 | 0.439 | | |  | ++ | | | | | ++ | | | | ++ | |
| Deoxyhexose+Hex | | | | | | | C12H24O11 | | | [M’+Cl]^-^ | | | 379.1013 | | | 0.016 | | 0.003 | 0.002 | | | | 0.16 | 0.12 | 0.390 | | |  | + | | | | | + | | | | + | |
|  | | | | | | |  | | |  | | |  | | |  | |  |  | | | |  |  |  | | |  |  | | | | |  | | | |  | |
| **Saccharic acids** | | | | | | |  | | |  | | |  | | |  | |  |  | | | |  |  |  | | |  |  | | | | |  | | | |  | |
| Glyceric acid | | | | | | | C3H6O4 | | | [M-H]^-^ | | | 105.0193 | | | 0.533 | | 0.223 | 0.090 | | | | 0.42 | 0.17 | 0.216 | | |  | ++++ | | | | | ++++ | | | | ++ | |
| Threonic acid | | | | | | | C4H8O5 | | | [M-H]^-^ | | | 135.0299 | | | 0.207 | | 0.119 | 0.167 | | | | 0.58 | 0.81 | 0.658 | | |  | ++++ | | | | | ++++ | | | | ++++ | |
| Galacturonic acid | | | | | | | C6H10O7 | | | [M-H]^-^ | | | 193.0354 | | | 0.477 | | 0.217 | 0.184 | | | | 0.46 | 0.39 | 0.146 | | |  | ++++ | | | | | ++++ | | | | ++++ | |
| Gluconic acid | | | | | | | C6H12O7 | | | [M-H]^-^ | | | 195.0510 | | | 0.126 | | 0.089 | 0.125 | | | | 0.70 | 0.99 | 0.797 | | |  | ++++ | | | | | ++++ | | | | ++++ | |
| Galactaric acid | | | | | | | C6H10O8 | | | [M-H]^-^ | | | 209.0303 | | | 0.050 | | 0.020 | 0.029 | | | | 0.41 | 0.59 | 0.188 | | |  | +++ | | | | | +++ | | | | +++ | |
|  | | | | | | |  | | |  | | |  | | |  | |  |  | | | |  |  |  | | |  |  | | | | |  | | | |  | |
| **Cluster ions** | | | | | | |  | | |  | | |  | | |  | |  |  | | | |  |  |  | | |  |  | | | | |  | | | |  | |
| Glyceric acid+Hex | | | | | | | C9H18O10 | | | [M’-H]^-^ | | | 285.0827 | | | 0.025 | | 0.002 | 0.001 | | | | 0.06 | 0.06 | 0.180 | | |  | + | | | | | + | | | | + | |
| Glyceric acid+Hex_2_ | | | | | | | C15H28O15 | | | [M’-H]^-^ | | | 447.1355 | | | 0.017 | | 0.015 | 0.041 | | | | 0.87 | 2.37 | 0.643 | | |  | +++ | | | | | +++ | | | | ++ | |
| Threonic acid+Hex | | | | | | | C10H20O11 | | | [M’-H]^-^ | | | 315.0933 | | | 0.080 | | 1.114 | 0.191 | | | | 13.97 | 2.40 | 0.309 | | |  | +++ | | | | | ++++ | | | | ++++ | |
| Threonic acid+Hex | | | | | | | C10H20O11 | | | [M’+Cl]^-^ | | | 351.0700 | | | 0.022 | | 0.024 | 0.042 | | | | 1.06 | 1.90 | 0.630 | | |  | ++ | | | | | +++ | | | | +++ | |
| Galacturonic acid+Hex | | | | | | | C12H22O13 | | | [M’-H]^-^ | | | 373.0988 | | | 0.003 | | 0.000 | 0.005 | | | | 0.06 | 1.75 | 0.359 | | |  | + | | | | | + | | | | ++ | |
| Galacturonic acid+Hex_2_ | | | | | | | C18H32O18 | | | [M’-H]^-^ | | | 535.1516 | | | 0.011 | | 0.007 | 0.032 | | | | 0.64 | 2.92 | 0.473 | | |  | ++ | | | | | ++ | | | | ++ | |
| Gluconic acid+Hex | | | | | | | C12H24O13 | | | [M’-H]^-^ | | | 375.1144 | | | 0.004 | | 0.046 | 0.047 | | | | 12.92 | 13.36 | 0.262 | | |  | ++ | | | | | +++ | | | | +++ | |
| Gluconic acid+Hex | | | | | | | C12H24O13 | | | [M’+Cl]^-^ | | | 411.0911 | | | 0.013 | | 0.026 | 0.000 | | | | 1.98 | 0.02 | 0.391 | | |  | ++ | | | | | + | | | | + | |
| Gluconic acid+Hex_2_ | | | | | | | C18H34O18 | | | [M’-H]^-^ | | | 537.1672 | | | 0.002 | | 0.002 | 0.003 | | | | 1.06 | 1.70 | 0.864 | | |  | + | | | | | + | | | | + | |
|  |  |  |  |  |  |  | | | | |  | | |  | | |  | | |  |  |  | | | |  | | | | | | | | | | | | | |

| (*Continued from previous page*.) |  |  |  | | | | | |  | |  |  | | | | | | | | | | |
| --- | --- | --- | --- | --- | --- | --- | --- | --- | --- | --- | --- | --- | --- | --- | --- | --- | --- | --- | --- | --- | --- | --- |
| Metabolites | Molecular  formula | Ion type detected  [M=molecule]  [M’=cluster] ^&^ | Theoretical*  *m/z* | Relative abundance (%)† | | | | | | *P* value | | | Frequency of detection‡ | | | | | | | | | |
|  |  |  |  | N | N(B) | N(W) | N(B) / N | N(W) / N | |  |  |  |  | N | | | N(B) | | | N(W) | | |
| **Alcohols** |  |  |  |  |  |  |  |  | |  | | |  | | | | | |  | | |  |
| Acetol | C3H6O2 | [M-H]^-^ | 73.0295 | 0.111 | 0.093 | 0.064 | 0.84 | 0.57 | | 0.739 | | |  | ++++ | | | +++ | | | ++ | | |
| Butanol | C4H10O | [M-H]^-^ | 73.0659 | 0.024 | 0.021 | 0.019 | 0.88 | 0.79 | | 0.870 | | |  | ++++ | | | ++++ | | | ++++ | | |
| Pentanol | C5H12O | [M-H]^-^ | 87.0815 | 0.012 | 0.001 | 0.008 | 0.08 | 0.68 | | 0.130 | | |  | +++ | | | ++ | | | ++ | | |
| Hexanol | C6H14O | [M-H]^-^ | 101.0972 | 0.000 | 0.006 | 0.000 | 36.74 | - | | 0.173 | | |  | + | | | ++ | | | - | | |
| Phenylethyl alcohol | C8H10O | [M-H]^-^ | 121.0659 | 0.103 | 0.068 | 0.067 | 0.67 | 0.65 | | 0.443 | | |  | ++++ | | | ++++ | | | +++ | | |
|  |  |  |  |  |  |  |  |  | |  | | |  |  | | |  | | |  | | |
| **Esters** |  |  |  |  |  |  |  |  | |  | | |  |  | | |  | | |  | | |
| Ethyl formate | C3H6O2 | [M-H]^-^ | 73.0295 | 0.072 | 0.078 | 0.064 | 1.09 | 0.89 | | 0.970 | | |  | +++ | | | +++ | | | ++ | | |
| Ethyl acetate | C4H8O2 | [M-H]^-^ | 87.0452 | 0.020 | 0.022 | 0.014 | 1.13 | 0.73 | | 0.901 | | |  | +++ | | | +++ | | | + | | |
| Methyl butanoate  Ethyl propanoate  Propyl acetate | C5H10O2 | [M-H]^-^ | 101.0608 | 0.187 | 0.128 | 0.094 | 0.68 | 0.50 | | 0.305 | | |  | ++++ | | | ++++ | | | ++++ | | |
| Methyl acetoacetate | C5H8O3 | [M-H]^-^ | 115.0401 | 0.254 | 0.251 | 0.061 | 0.99 | 0.24 | | 0.374 | | |  | ++++ | | | ++++ | | | ++++ | | |
| Ethyl butanoate  Propyl propanoate  Butyl acetate | C6H12O2 | [M-H]^-^ | 115.0765 | 0.503 | 0.349 | 0.270 | 0.69 | 0.54 | | 0.375 | | |  | ++++ | | | ++++ | | | ++++ | | |
| Methylbutyl acetate | C7H14O2 | [M-H]^-^ | 129.0921 | 0.275 | 0.160 | 0.128 | 0.58 | 0.46 | | 0.319 | | |  | ++++ | | | ++++ | | | ++++ | | |
| Dimethyl fumarate | C6H8O4 | [M-H]^-^ | 143.0350 | 3.540 | 2.869 | 2.984 | 0.81 | 0.84 | | 0.954 | | |  | ++++ | | | ++++ | | | ++++ | | |
| Ethyl hexanoate  Ppropyl methyl butanoate  Butyl butanoate  Hexyl acetate | C8H16O2 | [M-H]^-^ | 143.1078 | 1.003 | 0.626 | 0.644 | 0.62 | 0.64 | | 0.515 | | |  | ++++ | | | ++++ | | | ++++ | | |
| Propyl hexanoate  Butyl methylbutanoate | C9H18O2 | [M-H]^-^ | 157.1234 | 0.913 | 0.607 | 0.573 | 0.67 | 0.63 | | 0.528 | | |  | ++++ | | | ++++ | | | ++++ | | |
| Dimethyl malate | C6H10O5 | [M-H]^-^ | 161.0455 | 4.625 | 3.932 | 2.876 | 0.85 | 0.62 | | 0.756 | | |  | ++++ | | | ++++ | | | ++++ | | |
| Butyl hexanoate  Hexyl butanoate | C10H20O2 | [M-H]^-^ | 171.1391 | 0.840 | 0.745 | 0.491 | 0.89 | 0.58 | | 0.607 | | |  | ++++ | | | ++++ | | | ++++ | | |
| Gluconolactone | C6H10O6 | [M-H]^-^ | 177.0405 | 3.788 | 3.179 | 2.583 | 0.84 | 0.68 | | 0.883 | | |  | ++++ | | | ++++ | | | ++++ | | |
| Hexyl methylbutanoate | C11H22O2 | [M-H]^-^ | 185.1547 | 0.124 | 0.148 | 0.063 | 1.20 | 0.51 | | 0.507 | | |  | +++ | | | ++++ | | | +++ | | |
| Hexyl hexanoate | C12H24O2 | [M-H]^-^ | 199.1704 | 0.414 | 0.472 | 0.243 | 1.14 | 0.59 | | 0.478 | | |  | ++++ | | | ++++ | | | ++++ | | |
| Galacturonic acid methyl ester | C7H12O7 | [M-H]^-^ | 207.0510 | 0.300 | 0.071 | 0.063 | 0.24 | 0.21 | | 0.068 | | |  | ++++ | | | +++ | | | +++ | | |
|  |  |  |  |  |  |  |  |  | |  | | |  |  | | |  | | |  | | |
| **Cluster ions** |  |  |  |  |  |  |  |  | |  | | |  |  | | |  | | |  | | |
| Ethyl formate+Hex | C9H18O8 | [M’-H]^-^ | 253.0929 | 0.000 | 0.001 | 0.000 | - | - | | 0.282 | | |  | - | | | + | | | - | | |
| Ethyl formate+Hex_2_ | C15H28O13 | [M’-H]^-^ | 415.1457 | 0.000 | 0.000 | 0.000 | - | - | | 1.000 | | |  | - | | | - | | | - | | |
| Ethyl acetate+Hex | C10H20O8 | [M’-H]^-^ | 267.1086 | 0.000 | 0.007 | 0.001 | - | - | | 0.161 | | |  | - | | | ++ | | | + | | |
| Ethyl acetate+Hex_2_ | C16H30O13 | [M’-H]^-^ | 429.1614 | 0.001 | 0.000 | 0.001 | - | 1.19 | | 0.531 | | |  | + | | | - | | | + | | |
| Methyl butanoate+Hex  Ethyl propanoate+Hex  Propyl acetate+Hex | C11H22O8 | [M’-H]^-^ | 281.1242 | 0.000 | 0.000 | 0.000 | - | - | | 1.000 | | |  | - | | | - | | | - | | |
| Methyl butanoate+Hex_2_  Ethyl propanoate+Hex_2_  Propyl acetate+Hex_2_ | C17H32O13 | [M’-H]^-^ | 443.1770 | 0.001 | 0.000 | 0.000 | - | - | | 0.344 | | |  | + | | | - | | | - | | |
| Methyl butanoate+Hex_2_  Ethyl propanoate+Hex_2_  Propyl acetate+Hex_2_ | C17H32O13 | [M’+Cl]^-^ | 479.1537 | 0.016 | 0.000 | 0.016 | 0.02 | 0.97 | | 0.265 | | |  | ++ | | | + | | | ++ | | |
| Methyl acetoacetate+Hex | C11H20O9 | [M’-H]^-^ | 295.1035 | 0.005 | 0.000 | 0.005 | - | 1.11 | | 0.512 | | |  | + | | | - | | | ++ | | |
| Ethyl butanoate+Hex  Propyl propanoate+Hex  Iso-Butyl acetate+Hex | C12H24O8 | [M’-H]^-^ | 295.1399 | 0.001 | 0.000 | 0.000 | 0.48 | - | | 0.488 | | |  | + | | | + | | | - | | |
| Dimethyl fumarate+Hex | C12H20O10 | [M’-H]^-^ | 323.0984 | 0.279 | 0.057 | 0.011 | 0.21 | 0.04 | | 0.337 | | |  | +++ | | | +++ | | | ++ | | |
|  |  |  |  |  |  |  |  |  | |  | | | | |  |  | |  | | |  | |

| (*Continued from previous page*.) |  |  |  | | | | | |  | |  |  | | | | | | | |
| --- | --- | --- | --- | --- | --- | --- | --- | --- | --- | --- | --- | --- | --- | --- | --- | --- | --- | --- | --- |
| Metabolites | Molecular  formula | Ion type detected  [M=molecule]  [M’=cluster] ^&^ | Theoretical*  *m/z* | Relative abundance (%)† | | | | | | *P* value | | | Frequency of detection‡ | | | | | | |
|  |  |  |  | N | N(B) | N(W) | N(B) / N | N(W) / N | |  |  |  |  | N | | N(B) | | N(W) |  |
| Ethyl hexanoate+Hex  Propyl methyl butanoate+Hex  Butyl butanoate+Hex  Hexyl acetate+Hex | C14H28O8 | [M’-H]^-^ | 323.1712 | 0.000 | 0.000 | 0.000 | - | - | | 0.282 | | |  | - | | + | | - |  |
| Ethyl hexanoate+Hex_2_  Propyl methyl butanoate+Hex_2_  Butyl butanoate+Hex_2_  Hexyl acetate+Hex_2_ | C20H38O13 | [M’-H]^-^ | 485.2240 | 0.005 | 0.000 | 0.000 | - | - | | 0.344 | | |  | + | | - | | - |  |
| Butyl hexanoate+Hex  Hexyl butanoate+Hex | C16H32O8 | [M’-H]^-^ | 351.2025 | 0.000 | 0.004 | 0.000 | - | - | | 0.207 | | |  | - | | + | | - |  |
| Gluconolactone+Hex | C12H22O12 | [M’-H]^-^ | 357.1039 | 0.117 | 0.201 | 0.065 | 1.71 | 0.56 | | 0.471 | | |  | ++++ | | ++++ | | +++ |  |
| Gluconolactone+Hex_2_ | C18H32O17 | [M’-H]^-^ | 519.1567 | 0.008 | 0.003 | 0.014 | 0.31 | 1.66 | | 0.467 | | |  | ++ | | ++ | | ++ |  |
| Gluconolactone+Hex_3_ | C24H42O22 | [M’-H]^-^ | 681.2096 | 0.001 | 0.002 | 0.002 | 3.32 | 2.86 | | 0.651 | | |  | + | | + | | + |  |
| Galacturonic acid methyl ester+Hex | C13H24O13 | [M’-H]^-^ | 387.1144 | 0.349 | 1.317 | 0.162 | 3.78 | 0.46 | | 0.342 | | |  | +++ | | ++++ | | ++++ |  |
| Hexyl hexanoate+Hex | C18H36O8 | [M’-H]^-^ | 253.0929 | 0.000 | 0.002 | 0.002 | 16.70 | 23.54 | | 0.568 | | |  | + | | + | | + |  |
|  |  |  |  |  |  |  |  |  | |  | | |  |  | |  | |  |  |
| **Aldehydes** |  |  |  |  |  |  |  |  | |  | | |  |  | |  | |  |  |
| Glycidaldehyde | C3H4O2 | [M-H]^-^ | 71.0139 | 0.513 | 1.159 | 1.396 | 2.26 | 2.72 | | 0.676 | | |  | ++++ | | ++++ | | ++++ |  |
| Butanal | C4H8O | [M-H]^-^ | 71.0502 | 0.004 | 0.005 | 0.000 | 1.17 | - | | 0.290 | | |  | ++ | | ++ | | - |  |
| Furfural | C5H4O2 | [M-H]^-^ | 95.0139 | 0.228 | 0.069 | 0.065 | 0.30 | 0.28 | | 0.262 | | |  | ++++ | | ++++ | | ++ |  |
| Hexenal | C6H10O | [M-H]^-^ | 97.0659 | 0.021 | 0.012 | 0.020 | 0.56 | 0.94 | | 0.755 | | |  | +++ | | +++ | | +++ |  |
| Hexanal | C6H12O | [M-H]^-^ | 99.0815 | 0.008 | 0.000 | 0.002 | 0.04 | 0.30 | | 0.215 | | |  | +++ | | + | | + |  |
| Succinic acid semialdehyde | C4H6O3 | [M-H]^-^ | 101.0244 | 2.345 | 2.433 | 2.396 | 1.04 | 1.02 | | 0.999 | | |  | ++++ | | ++++ | | ++++ |  |
| Benzaldehyde | C7H6O | [M-H]^-^ | 105.0346 | 0.000 | 0.001 | 0.004 | 3.25 | 14.84 | | 0.181 | | |  | + | | ++ | | ++ |  |
| Heptenal | C7H12O | [M-H]^-^ | 111.0815 | 0.011 | 0.018 | 0.004 | 1.61 | 0.32 | | 0.290 | | |  | +++ | | +++ | | ++ |  |
| Octanal | C8H16O | [M-H]^-^ | 127.1128 | 0.021 | 0.009 | 0.006 | 0.45 | 0.29 | | 0.324 | | |  | +++ | | ++ | | + |  |
| Nonanal | C9H18O | [M-H]^-^ | 141.1285 | 0.013 | 0.003 | 0.003 | 0.23 | 0.24 | | 0.095 | | |  | +++ | | + | | + |  |
| Decanal | C10H20O | [M-H]^-^ | 155.1441 | 0.009 | 0.002 | 0.000 | 0.25 | 0.05 | | 0.109 | | |  | ++ | | + | | + |  |
|  |  |  |  |  |  |  |  |  | |  | | |  |  | |  | |  |  |
| **Cluster ions** |  |  |  |  |  |  |  |  | |  | | |  |  | |  | |  |  |
| Glycidaldehyde+Hex | C9H16O8 | [M’-H]^-^ | 251.0773 | 0.021 | 0.011 | 0.003 | 0.52 | 0.14 | | 0.243 | | |  | ++ | | +++ | | + |  |
|  |  |  |  |  |  |  |  |  | |  | | |  |  | |  | |  |  |
| **Ketone** |  |  |  |  |  |  |  |  | |  | | |  |  | |  | |  |  |
| Methylheptenone | C8H14O | [M-H]^-^ | 125.0972 | 0.013 | 0.003 | 0.005 | 0.22 | 0.34 | | 0.169 | | |  | +++ | | ++ | | ++ |  |
|  |  |  |  |  |  |  |  |  | |  | | |  |  | |  | |  |  |
| **Phenylpropene** |  |  |  |  |  |  |  |  | |  | | |  |  | |  | |  |  |
| Estragole | C10H12O | [M-H]^-^ | 147.0815 | 0.011 | 0.007 | 0.010 | 0.68 | 0.88 | | 0.905 | | |  | +++ | | ++ | | + |  |
|  |  |  |  |  |  |  |  |  | |  | | |  |  | |  | |  |  |
| **Terpene** |  |  |  |  |  |  |  |  | |  | | |  |  | |  | |  |  |
| Farnesene | C15H24 | [M-H]^-^ | 203.1805 | 0.001 | 0.000 | 0.000 | - | - | | 0.344 | | |  | + | | - | | - |  |
|  |  |  |  |  |  |  |  |  | |  | | |  |  | |  | |  |  |
| **Lipid** |  |  |  |  |  |  |  |  | |  | | |  |  | |  | |  |  |
| Phosphatidyl glycerol | C6H15O8P | [M-H]^-^ | 245.0432 | 0.093 | 0.073 | 0.087 | 0.79 | 0.94 | | 0.929 | | |  | +++ | | ++++ | | +++ |  |
|  |  |  |  |  |  |  |  |  | |  | | |  |  | |  | |  |  |
| **Cluster ion** |  |  |  |  |  |  |  |  | |  | | |  |  | |  | |  |  |
| Phosphatidyl glycerol+Hex | C12H27O14P | [M’-H]^-^ | 425.1066 | 0.000 | 0.000 | 0.004 | - | - | | 0.403 | | |  | - | | - | | + |  |
|  |  |  |  |  |  |  |  |  | |  | | |  |  |  | |  | |  |

| (*Continued from previous page*.) |  |  |  | | | | | |  | |  |  | | | | | | | | | |  |
| --- | --- | --- | --- | --- | --- | --- | --- | --- | --- | --- | --- | --- | --- | --- | --- | --- | --- | --- | --- | --- | --- | --- |
| Metabolites | Molecular  formula | Ion type detected  [M=molecule]  [M’=cluster] ^&^ | Theoretical*  *m/z* | Relative abundance (%)† | | | | | | *P* value | | | Frequency of detection‡ | | | | | | | | |  |
|  |  |  |  | N | N(B) | N(W) | N(B) / N | N(W) / N | |  |  |  |  | N | | N(B) | | | | N(W) |  |  |
| **Cell wall synthesis metabolism** |  |  |  |  |  |  |  |  | |  | | |  | | | |  | | | | |  |
| Glycerol | C3H8O3 | [M-H]^-^ | 91.0401 | 0.017 | 0.006 | 0.013 | 0.37 | 0.74 | | 0.516 | | |  | +++ | | ++ | | | | ++ |  |  |
| Glycerol | C3H8O3 | [M+Cl]^-^ | 127.0167 | 0.004 | 0.000 | 0.000 | - | - | | 0.334 | | |  | + | | - | | | | - |  |  |
| Coumaryl-alcohol | C9H10O2 | [M-H]^-^ | 149.0608 | 0.043 | 0.026 | 0.023 | 0.61 | 0.53 | | 0.476 | | |  | +++ | | ++++ | | | | ++ |  |  |
| Shikimic acid | C7H10O5 | [M-H]^-^ | 173.0455 | 0.093 | 0.026 | 0.052 | 0.28 | 0.57 | | 0.326 | | |  | +++ | | ++++ | | | | ++++ |  |  |
| Coniferyl alcohol | C10H12O3 | [M-H]^-^ | 179.0714 | 0.005 | 0.001 | 0.000 | 0.15 | - | | 0.171 | | |  | + | | + | | | | - |  |  |
| Ferulic acid | C10H10O4 | [M-H]^-^ | 193.0506 | 0.020 | 0.008 | 0.005 | 0.38 | 0.23 | | 0.286 | | |  | +++ | | ++ | | | | ++ |  |  |
| Sinapyl-alcohol | C11H14O4 | [M-H]^-^ | 209.0819 | 0.017 | 0.005 | 0.005 | 0.29 | 0.28 | | 0.279 | | |  | ++ | | + | | | | ++ |  |  |
| UDP-Pentose | C14H22N2O16P2 | [M-H]^-^ | 535.0372 | 0.000 | 0.000 | 0.005 | - | - | | 0.133 | | |  | - | | - | | | | ++ |  |  |
|  |  |  |  |  |  |  |  |  | |  | | |  |  | |  | | | |  |  |  |
| **Cluster ions** |  |  |  |  |  |  |  |  | |  | | |  |  | |  | | | |  |  |  |
| Glycerol+Hex | C9H20O9 | [M’-H]^-^ | 271.0350 | 0.010 | 0.073 | 0.037 | 6.97 | 3.53 | | 0.548 | | |  | + | | ++ | | | | ++ |  |  |
| Glycerol+Hex | C9H20O9 | [M’+Cl]^-^ | 307.0802 | 0.001 | 0.000 | 0.000 | - | - | | 0.344 | | |  | + | | - | | | | - |  |  |
| Shikimic acid+Hex | C13H22O11 | [M’-H]^-^ | 353.1089 | 0.169 | 0.020 | 0.164 | 0.12 | 1.01 | | 0.510 | | |  | +++ | | +++ | | | | +++ |  |  |
| Shikimic acid+Hex_2_ | C19H32O16 | [M’-H]^-^ | 515.1617 | 0.003 | 0.003 | 0.009 | 1.12 | 2.81 | | 0.537 | | |  | + | | + | | | | ++ |  |  |
| Ferulic acid+Hex_2_ | C22H32O15 | [M’-H]^-^ | 535.1668 | 0.265 | 0.025 | 0.033 | 0.09 | 0.13 | | 0.328 | | |  | +++ | | +++ | | | | ++ |  |  |
| Sinapyl-alcohol+Hex | C17H26O10 | [M’-H]^-^ | 389.1453 | 0.001 | 0.001 | 0.001 | 0.92 | 1.18 | | 0.983 | | |  | + | | + | | | | + |  |  |
| UDP-Pentose+Hex | C20H34N2O22P2 | [M’-H]^-^ | 715.1006 | 0.043 | 0.307 | 0.152 | 7.07 | 3.49 | | 0.388 | | |  | +++ | | +++ | | | | +++ |  |  |
|  |  |  |  |  |  |  |  |  | |  | | |  |  | |  | | | |  |  |  |
| **Phytohormones and the precursors** | |  |  |  |  |  |  |  | |  | | |  | | | | |  | | | |  |
| 1-Aminocyclopropane-1-carboxylic acid | C4H7NO2 | [M-H]^-^ | 100.0404 | 0.000 | 0.002 | 0.002 | 5.15 | 3.78 | | 0.688 | | |  | + | | + | | | | + |  |  |
| 1-Aminocyclopropane-1-carboxylic acid | C4H7NO2 | [M+Cl]^-^ | 136.0171 | 0.191 | 0.106 | 0.074 | 0.56 | 0.39 | | 0.280 | | |  | ++++ | | ++++ | | | | +++ |  |  |
| Salicylic acid | C7H6O3 | [M-H]^-^ | 137.0244 | 0.159 | 0.039 | 0.039 | 0.25 | 0.25 | | 0.205 | | |  | +++ | | +++ | | | | +++ |  |  |
| 3-Indoleacetic Acid | C10H9NO2 | [M-H]^-^ | 174.0561 | 0.003 | 0.007 | 0.001 | 2.48 | 0.50 | | 0.552 | | |  | + | | + | | | | + |  |  |
| Zeatin | C10H13N5O | [M-H]^-^ | 218.1047 | 0.000 | 0.000 | 0.005 | - | - | | 0.094 | | |  | - | | - | | | | ++ |  |  |
| Abscisic acid | C15H20O4 | [M-H]^-^ | 263.1289 | 0.001 | 0.000 | 0.000 | - | 0.26 | | 0.459 | | |  | + | | - | | | | + |  |  |
| Gibberellin | C19H22O6 | [M-H]^-^ | 345.1344 | 0.000 | 0.000 | 0.000 | - | - | | 1.000 | | |  | - | | - | | | | - |  |  |
|  |  |  |  |  |  |  |  |  | |  | | |  |  | |  | | | |  |  |  |
| **Cluster ions** |  |  |  |  |  |  |  |  | |  | | |  |  | |  | | | |  |  |  |
| 1-Aminocyclopropane-1-carboxylic acid+Hex | C10H19NO8 | [M’-H]^-^ | 280.1038 | 0.020 | 0.070 | 0.022 | 3.55 | 1.14 | | 0.475 | | |  | ++ | | ++ | | | | ++ |  |  |
| 1-Aminocyclopropane-1-carboxylic acid+Hex | C10H19NO8 | [M’+Cl]^-^ | 316.0805 | 0.004 | 0.000 | 0.010 | - | 2.65 | | 0.519 | | |  | + | | - | | | | + |  |  |
| Salicylic acid+Hex | C13H18O9 | [M’-H]^-^ | 317.0878 | 0.054 | 0.025 | 0.058 | 0.45 | 1.08 | | 0.660 | | |  | +++ | | ++ | | | | ++ |  |  |
| Salicylic acid+Hex | C13H18O9 | [M’+Cl]^-^ | 353.0645 | 0.000 | 0.000 | 0.001 | - | - | | 0.403 | | |  | - | | - | | | | + |  |  |
| Salicylic acid+Hex_2_ | C19H28O14 | [M’-H]^-^ | 479.1406 | 0.003 | 0.000 | 0.000 | 0.16 | - | | 0.397 | | |  | + | | + | | | | - |  |  |
| Gibberellin+Hex | C25H34O12 | [M’-H]^-^ | 525.1978 | 0.000 | 0.000 | 0.000 | - | - | | 1.000 | | |  | - | | - | | | | - |  |  |
| Gibberellin+Hex_2_ | C31H44O17 | [M’-H]^-^ | 687.2506 | 0.000 | 0.000 | 0.000 | - | - | | 1.000 | | |  | - | | - | | | | - |  |  |
|  |  |  |  |  |  |  |  |  | |  | | |  |  |  | | | |  | |  |  |

| (*Continued from previous page*.) |  | |  |  | | |  | | |  | |  | | | | | | |  | | |  |  | | | | | | | | | | | | |
| --- | --- | --- | --- | --- | --- | --- | --- | --- | --- | --- | --- | --- | --- | --- | --- | --- | --- | --- | --- | --- | --- | --- | --- | --- | --- | --- | --- | --- | --- | --- | --- | --- | --- | --- | --- |
| Metabolites | Molecular  formula | | Ion type detected  [M=molecule]  [M’=cluster] ^&^ | | | Theoretical*  *m/z* | | Relative abundance (%)† | | | | | | | | | | | | *P* value | | | |  | | Frequency of detection‡ | | | | | | | | | |
|  |  |  |  |  |  |  |  | N | | | N(B) | | N(W) | | N(B) / N | | N(W) / N | | |  |  |  |  |  | | N | | | | N(B) | | | | N(W) | |
| **Organic acids** |  | |  | | |  | |  | | |  | |  | |  | |  | | |  | | | | |  | | | |  | | | |  | | |
| Glyoxylic acid | C2H2O3 | | [M-H]^-^ | | | 72.9931 | | 0.286 | | | 0.072 | | 0.147 | | 0.25 | | 0.51 | | | 0.321 | | | |  | | +++ | | | | ++++ | | | | ++++ | |
| Glycolic acid | C2H4O3 | | [M-H]^-^ | | | 75.0088 | | 0.279 | | | 0.242 | | 0.058 | | 0.87 | | 0.21 | | | 0.416 | | | |  | | ++++ | | | | +++ | | | | ++ | |
| Hydroxypyruvic acid | C3H4O4 | | [M-H]^-^ | | | 103.0037 | | 1.944 | | | 3.368 | | 0.235 | | 1.73 | | 0.12 | | | 0.415 | | | |  | | +++ | | | | +++ | | | | +++ | |
| Citramalic acid | C5H8O6 | | [M-H]^-^ | | | 147.0299 | | 0.642 | | | 0.313 | | 0.403 | | 0.49 | | 0.63 | | | 0.417 | | | |  | | ++++ | | | | ++++ | | | | ++++ | |
| Ascorbic acid | C6H8O6 | | [M-H]^-^ | | | 175.0247 | | 1.140 | | | 0.291 | | 0.320 | | 0.26 | | 0.28 | | | 0.145 | | | |  | | ++++ | | | | ++++ | | | | ++++ | |
| Quinic acid | C7H12O6 | | [M-H]^-^ | | | 191.0561 | | 3.579 | | | 7.218 | | 6.858 | | 2.02 | | 1.92 | | | 0.418 | | | |  | | ++++ | | | | ++++ | | | | ++++ | |
| Homocitric acid | C7H10O7 | | [M-H]^-^ | | | 205.0354 | | 0.310 | | | 0.087 | | 0.090 | | 0.28 | | 0.29 | | | 0.082 | | | |  | | ++++ | | | | ++++ | | | | ++++ | |
| Chlorogenic acid | C16H18O9 | | [M-H]^-^ | | | 353.0875 | | 0.287 | | | 0.203 | | 0.387 | | 0.71 | | 1.35 | | | 0.304 | | | |  | | ++++ | | | | ++++ | | | | ++++ | |
|  |  | |  | | |  | |  | | |  | |  | |  | |  | | |  | | | |  | |  | | | |  | | | |  | |
| **Cluster ions** |  | |  | | |  | |  | | |  | |  | |  | |  | | |  | | | |  | |  | | | |  | | | |  | |
| Glyoxylic acid+Hex | C8H14O9 | | [M’-H]^-^ | | | 253.0565 | | 0.331 | | | 0.229 | | 0.110 | | 0.69 | | 0.33 | | | 0.574 | | | |  | | ++++ | | | | ++ | | | | +++ | |
| Glycolic acid+Hex | C8H16O9 | | [M’-H]^-^ | | | 255.0722 | | 0.309 | | | 0.281 | | 0.097 | | 0.91 | | 0.31 | | | 0.548 | | | |  | | ++++ | | | | ++++ | | | | ++ | |
| Hydroxypyruvic acid+Hex | C9H16O10 | | [M’-H]^-^ | | | 283.0671 | | 0.047 | | | 0.072 | | 0.067 | | 1.52 | | 1.41 | | | 0.931 | | | |  | | ++ | | | | ++ | | | | ++ | |
| Hydroxypyruvic acid+Hex_2_ | C15H26O15 | | [M’-H]^-^ | | | 445.1199 | | 0.018 | | | 0.003 | | 0.022 | | 0.16 | | 1.23 | | | 0.582 | | | |  | | +++ | | | | + | | | | ++ | |
| Glycolic acid+Hex_2_ | C14H26O14 | | [M’-H]^-^ | | | 417.1250 | | 0.026 | | | 0.023 | | 0.005 | | 0.89 | | 0.20 | | | 0.188 | | | |  | | +++ | | | | +++ | | | | ++ | |
| Citramalic acid+Hex | C11H20O12 | | [M’-H]^-^ | | | 327.0933 | | 0.578 | | | 0.138 | | 0.511 | | 0.24 | | 0.88 | | | 0.545 | | | |  | | +++ | | | | +++ | | | | +++ | |
| Citramalic acid+Hex_2_ | C17H30O17 | | [M’-H]^-^ | | | 489.1461 | | 0.001 | | | 0.000 | | 0.006 | | 0.24 | | 9.40 | | | 0.051 | | | |  | | + | | | | + | | | | ++ | |
| Ascorbic acid+Hex | C12H20O12 | | [M’-H]^-^ | | | 355.0882 | | 0.031 | | | 0.026 | | 0.041 | | 0.84 | | 1.29 | | | 0.899 | | | |  | | +++ | | | | +++ | | | | ++ | |
| Quinic acid+Hex | C13H24O12 | | [M’-H]^-^ | | | 371.1195 | | 0.195 | | | 0.355 | | 0.421 | | 1.82 | | 2.15 | | | 0.355 | | | |  | | ++++ | | | | ++++ | | | | +++ | |
| Quinic acid+Hex_2_ | C19H34O17 | | [M’-H]^-^ | | | 533.1723 | | 0.113 | | | 0.162 | | 0.307 | | 1.42 | | 2.70 | | | 0.192 | | | |  | | +++ | | | | ++++ | | | | +++ | |
| Chlorogenic acid+Hex | C22H30O15 | | [M’-H]^-^ | | | 533.1509 | | 0.005^b^ | | | 0.017^ab^ | | 0.059^a^ | | 3.66 | | 12.45 | | | 0.019 | | | |  | | + | | | | ++ | | | | +++ | |
| Chlorogenic acid+Hex_2_ | C28H40O20 | | [M’-H]^-^ | | | 695.2037 | | 0.000 | | | 0.014 | | 0.027 | | 52.41 | | 103.35 | | | 0.188 | | | |  | | + | | | | ++ | | | | ++ | |
|  | |  | | |  | | | |  | | |  | |  | |  | |  | | |  | | | | | |  |  | | |  |  | | |  |
| * All the theoretical values are quoted from Metlin (http://metlin.scripps.edu/index.php).  † Values were calculated as a percentage to the base peak. Different letters indicate a significant difference (Tukey-Kramer test, p < 0.05)  ‡ Signal detected (+); Signal not detected (-). Frequencies of detection (x) in 4 individual measurements are indicated as: ++++; 75%≦ x < 100%, +++; 50%≦ x < 75%, ++; 25%≦ x < 50%, +; 0%< x <25%.  ^&^ These clusters are the molecular aggregates stably formed in solution and in gas state because the intermolecular interaction among the molecular unities is quite strong (e.g., hydrogen bridge); its formation depends on the concentration of the molecular constituent in the analysed solution; at higher concentrations, the chance to obtain cluster signals in electrospray ionization mass spectrometry is higher. PicoPPESI mass spectrometry is an electrospray ionization mass spectrometry technique. | | | | | | | | | | | | | | | | | | | | | | | | | | | | | | | | | | | |

**Table S3. Cell area and the number and size of two major organelles in the cytosol of the cells located at normal outer parenchyma region and watercore region in watercored apples.** Cell area, the number and average area of mitochondria, and whole area and its ratio to cell area of vacuole-like structures are shown. The data are the means±SE of 23-26 cells collected from each region in 7 fruit. Apoplastic space per cell indicates the means±SE of 9 sections collected from 3 fruit. The *p* values were determined by *t*-test.

|  | Normal | Watercore | *P* value |
| --- | --- | --- | --- |
| Cell area (*µm^2^*) | 24458.6 | 22316.7 | 0.633 |
| Apoplastic space per cell (*%*) | 26.2 | 24.9 | 0.515 |
| Mitochondria |  |  |  |
| Number per cell  (*number ^.^ cell^-1^*) | 11.9 | 12.6 | 0.752 |
| Average area  (*µm^2^*) | 0.14 | 0.12 | 0.263 |
| Small vacuole-like structures |  |  |  |
| Whole area per cell  (*µm^2^ ^.^ cell^-1^*) | 13.2 | 18.5 | 0.403 |
| Ratio to cell area  (*%*) | 0.060 | 0.093 | 0.210 |

**Figure S1**

**
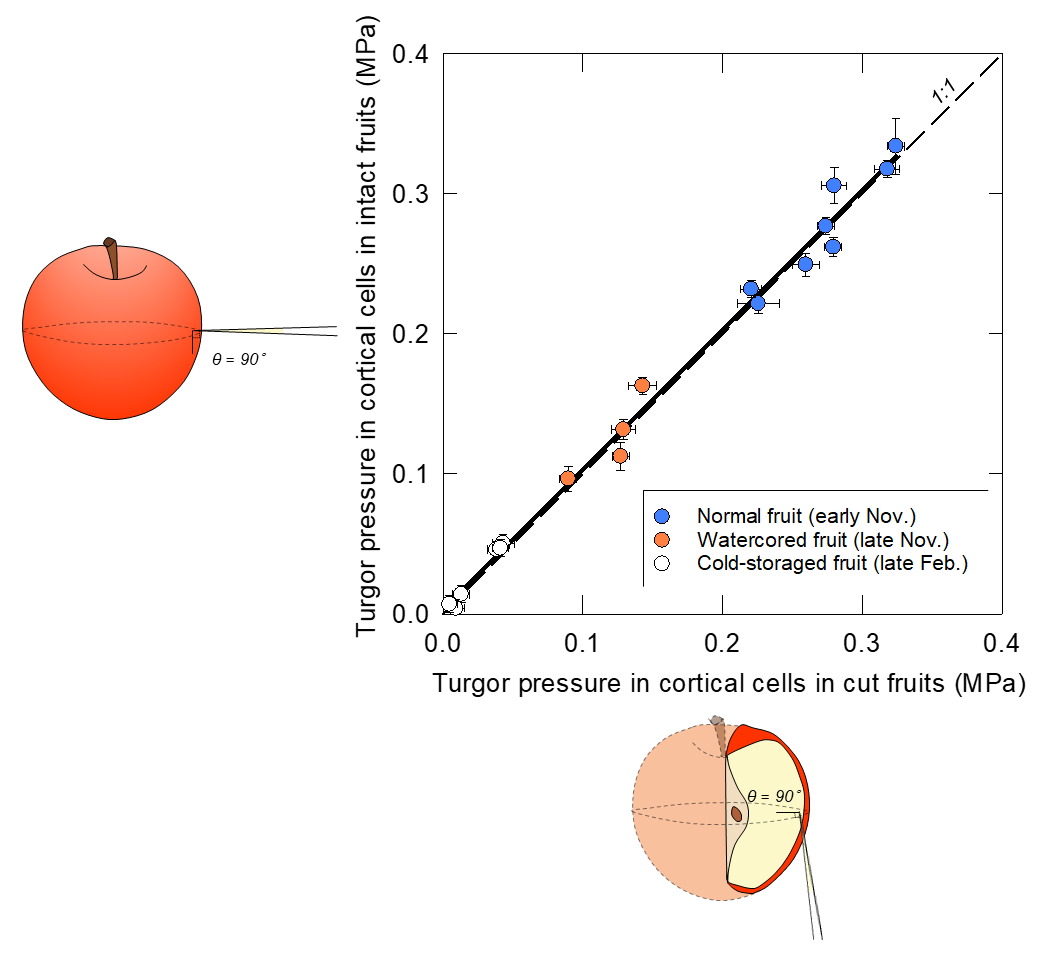
**

**Figure S1. Turgor pressure in cortical cells in intact fruit plotted against turgor pressure of the corresponding cortical cells in one of ‘Fuji’ apple segments after cutting into quarters**. By using a cell pressure probe, turgor pressure of the cortical cells, located between 300-1500 μm below epidermis in intact fruit, were assayed. And thereafter, the fruit were cut into quarters under humid conditions, and then the newly cut ¼ segment was fixed on the sample holder, so that the probe tip could be perpendicularly inserted into the corresponding region of the same fruit through the cut surface (see illustration). And then, turgor was determined in 30 min, avoiding water loss after cutting. Each point indicates the means±SE of 2-6 cells collected from each fruit. A slanting dotted line with (1:1) indicates an equipotential line. The solid line indicates regression line. The regression line between turgor in cut fruit (*x*) and turgor in intact fruit (*y*) was *y* = 1.005*x* - 0.002 with r^2^ = 0.99 (*p*<0.001).

**Figure S2-1**


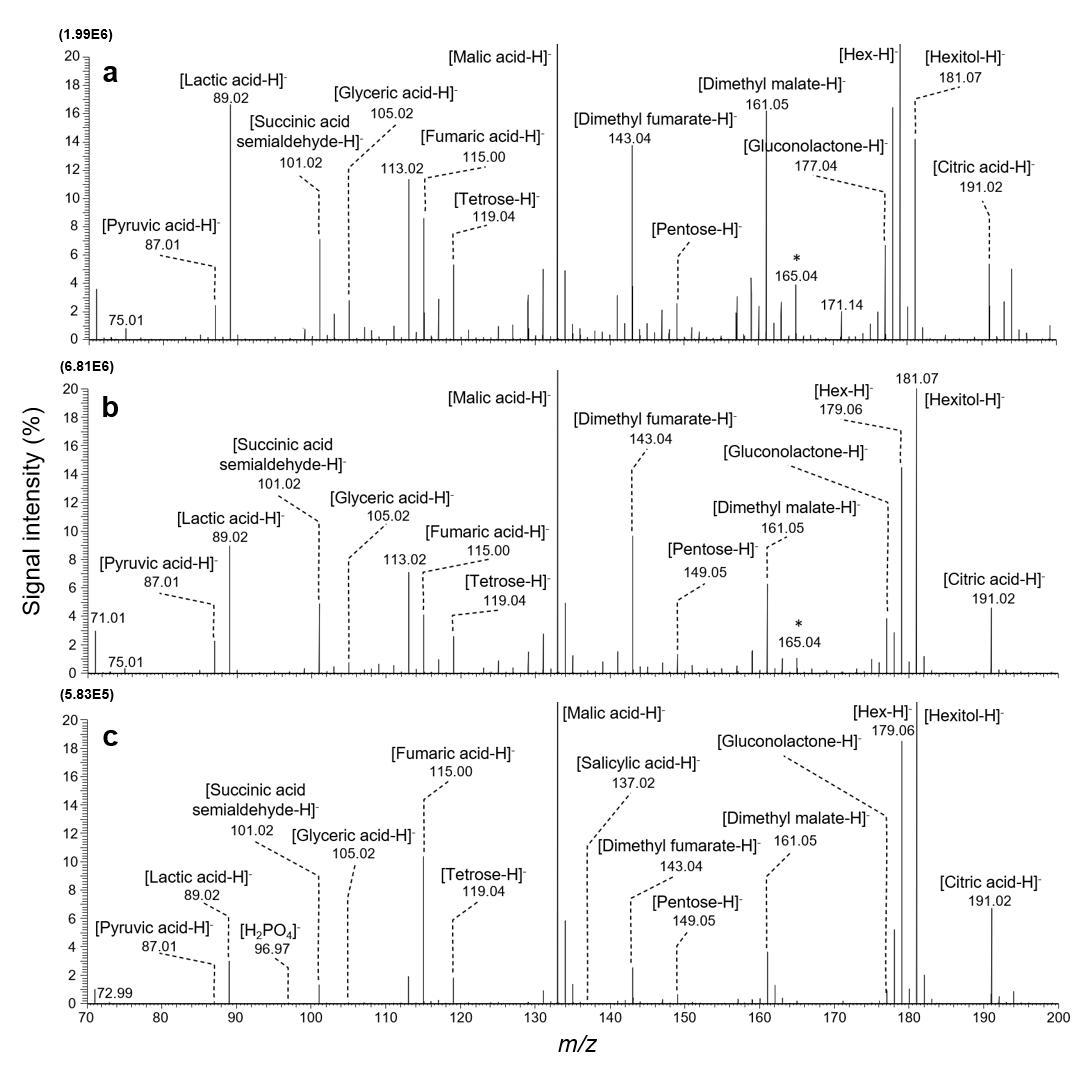


**Figure S2-1. The range of *m/z* 70-200 in picoPPESI negative ion mode mass spectra obtained from mesocarp cells of each region in watercored apple fruit.** Data in normal outer parenchyma (**a**), border (**b**), and watercore (**c**) regions are representative of similar experiments with 10 apples. Asterisk markings indicate assigned background peaks from mixture silicone oil+ionic solution filling into the pressure probe capillary. The full mass spectra are shown in Fig. 2. Hex: hexose.

**Figure S2-2**


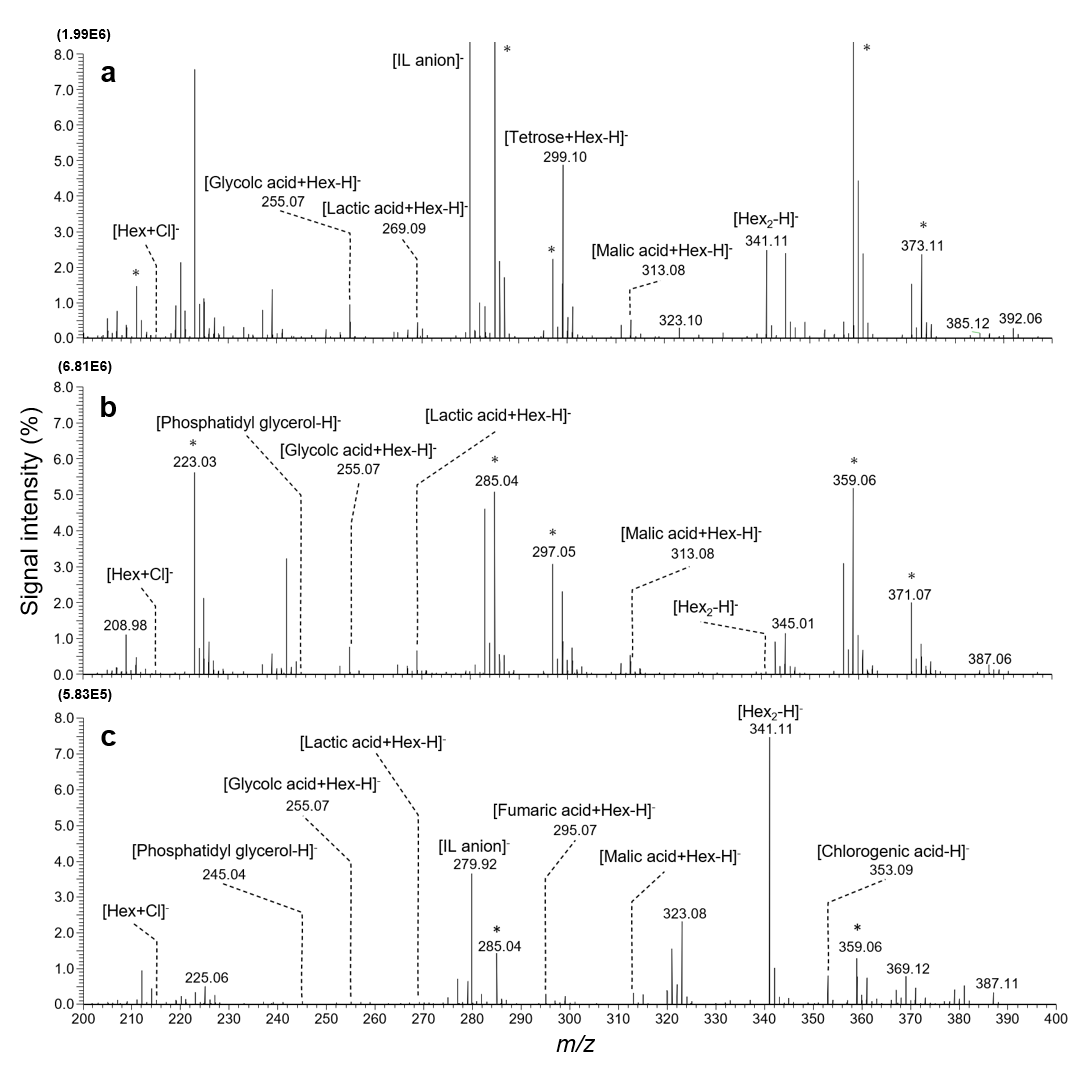


**Figure S2-2. The range of *m/z* 200-400 in picoPPESI negative ion mode mass spectra obtained from mesocarp cells of each region in watercored apple fruit.** Data in normal outer parenchyma (**a**), border (**b**), and watercore (**c**) regions are representative of similar experiments with 10 apples. Asterisk markings indicate assigned background peaks from mixture silicone oil+ionic solution filling into the pressure probe capillary. The full mass spectra are shown in Fig. 2. Hex: hexose; IL: ionic liquid.

**Figure S2-3**


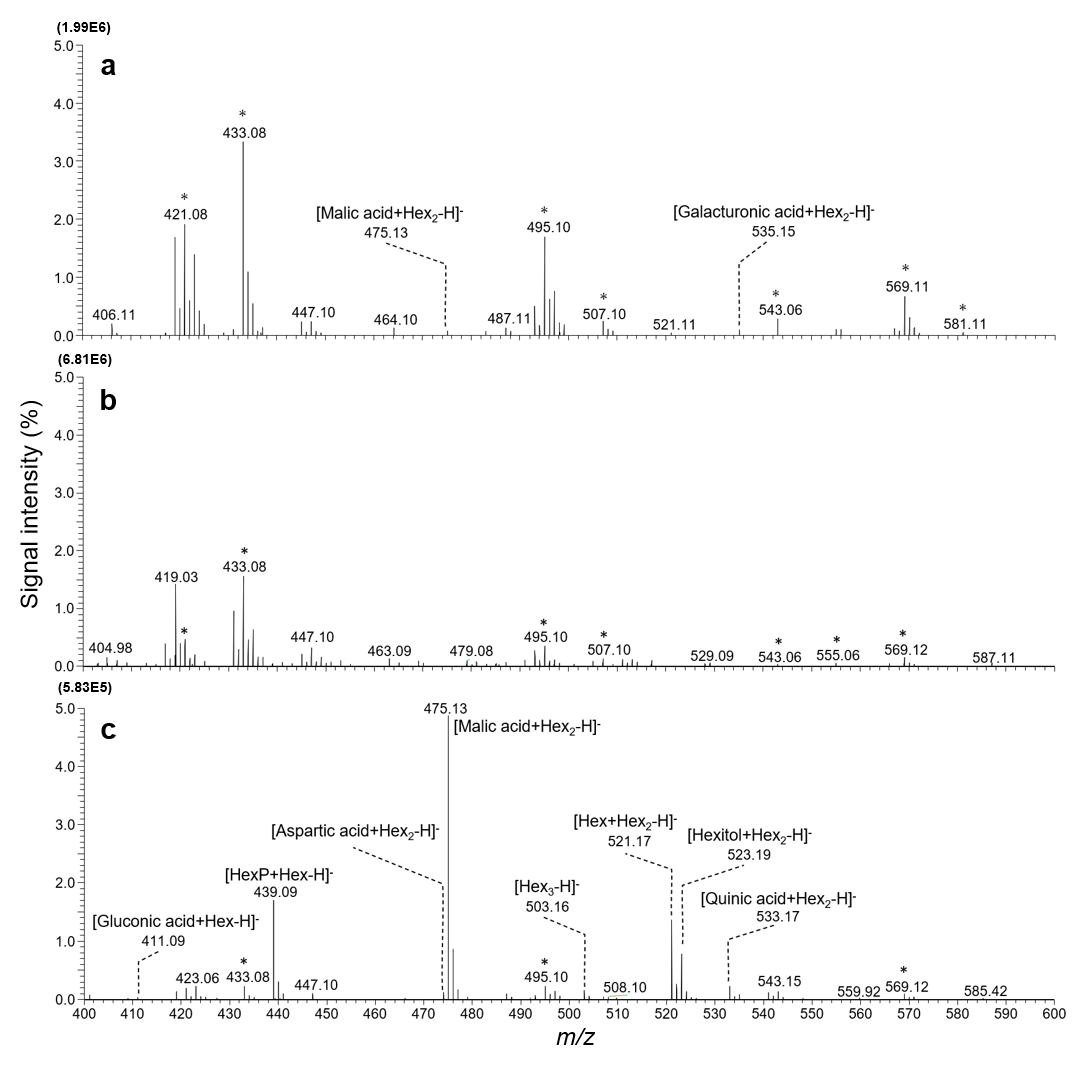


**Figure S2-3. The range of *m/z* 400-600 in picoPPESI negative ion mode mass spectra obtained from mesocarp cells of each region in watercored apple fruit.** Data in normal outer parenchyma (**a**), border (**b**), and watercore (**c**) regions are representative of similar experiments with 10 apples. Asterisk markings indicate assigned background peaks from mixture silicone oil+ionic solution filling into the pressure probe capillary. The full mass spectra are shown in Fig. 2. Hex: hexose.

**Figure S2-4**


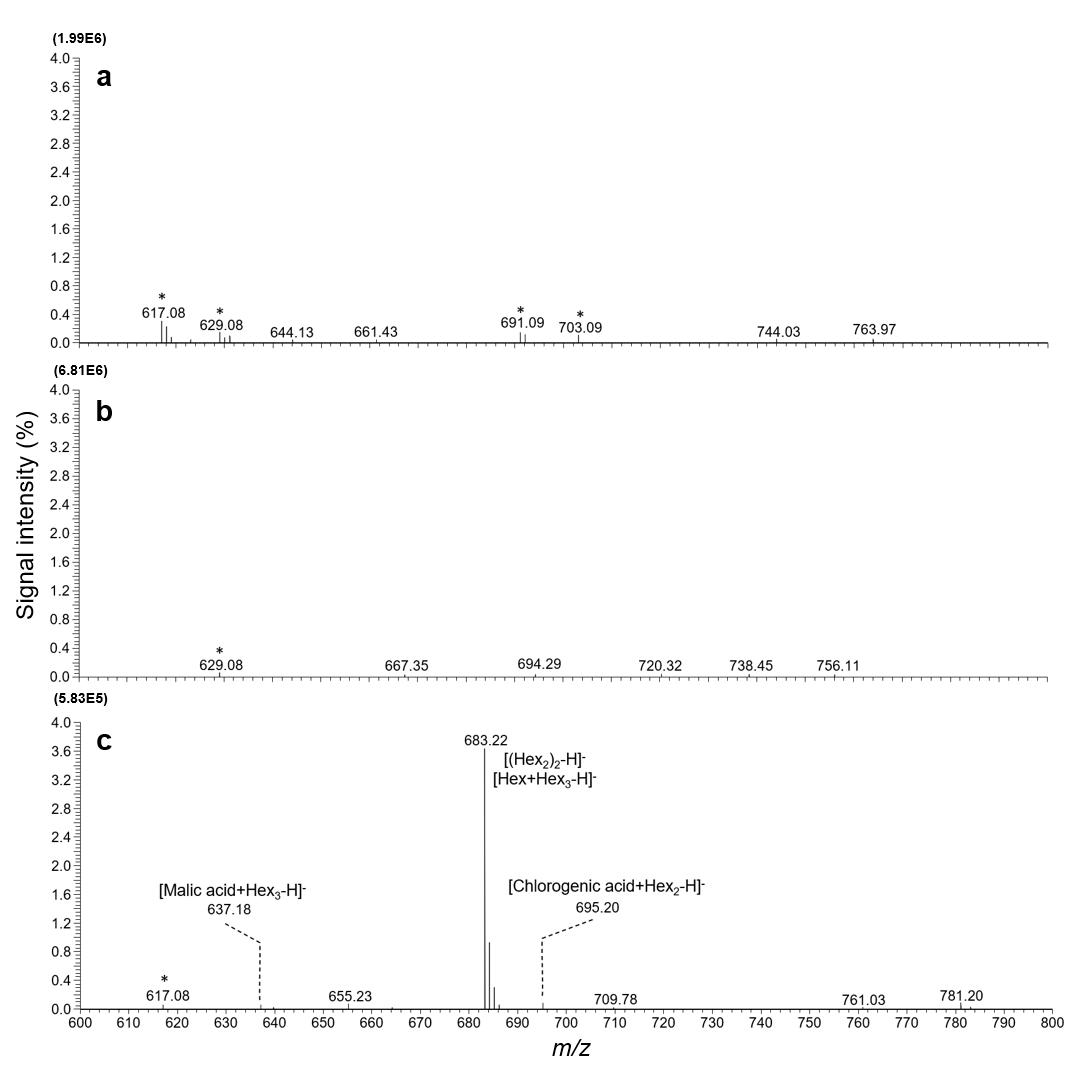


**Figure S2-4. The range of *m/z* 600-800 in picoPPESI negative ion mode mass spectra obtained from mesocarp cells of each region in watercored apple fruit.** Data in normal outer parenchyma (**a**), border (**b**), and watercore (**c**) regions are representative of similar experiments with 10 apples. Asterisk markings indicate assigned background peaks from mixture silicone oil+ionic solution filling into the pressure probe capillary. The full mass spectra are shown in Fig. 2. Hex: hexose.

**Figure S2-5**


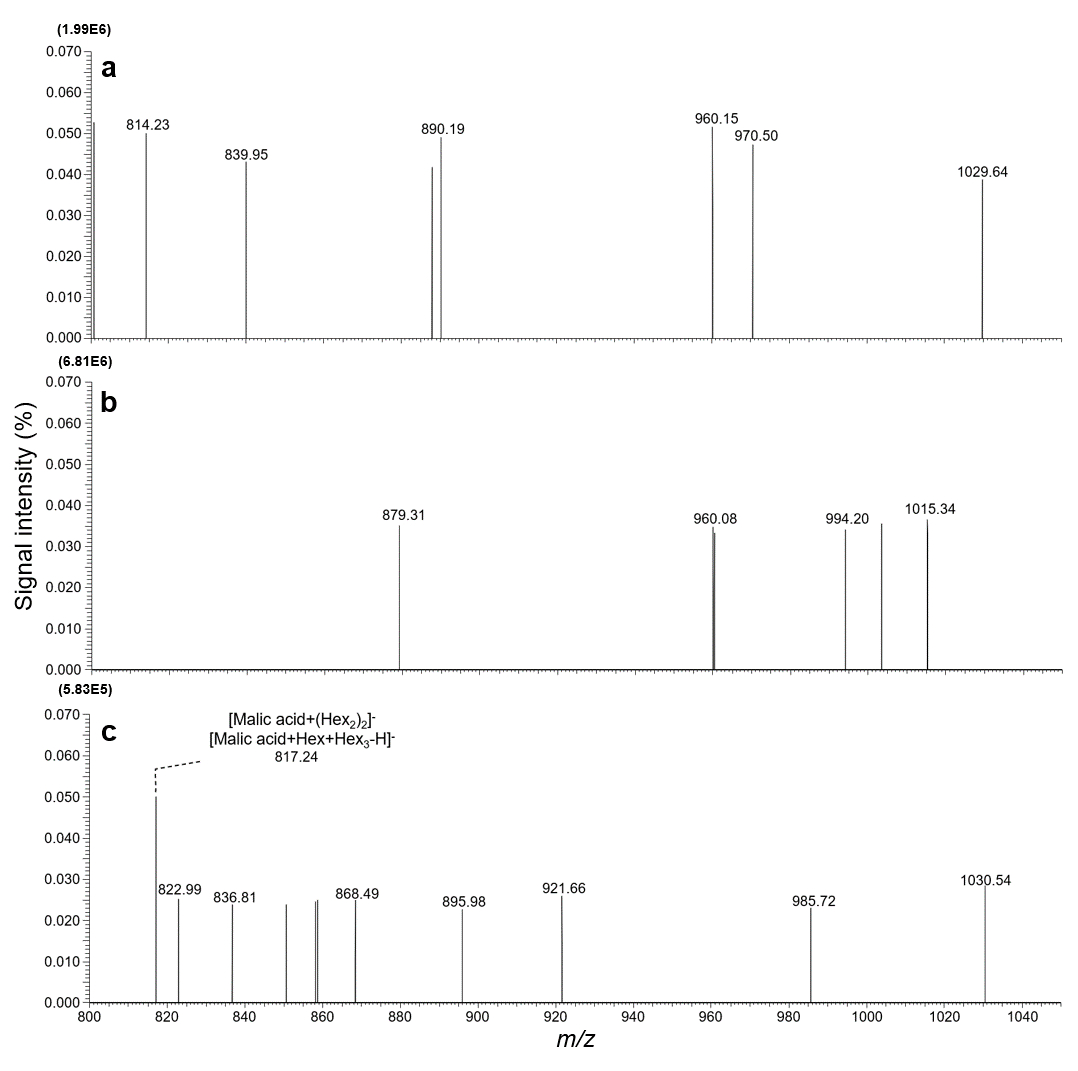


**Figure S2-5. The range of *m/z* 800-1050 in picoPPESI negative ion mode mass spectra obtained from mesocarp cells of each region in watercored apple fruit.** Data in normal outer parenchyma (**a**), border (**b**), and watercore (**c**) regions are representative of similar experiments with 10 apples Asterisk markings indicate assigned background peaks from mixture silicone oil+ionic solution filling into the pressure probe capillary. The full mass spectra are shown in Fig. 2. Hex: hexose.

**Figure S3-1**


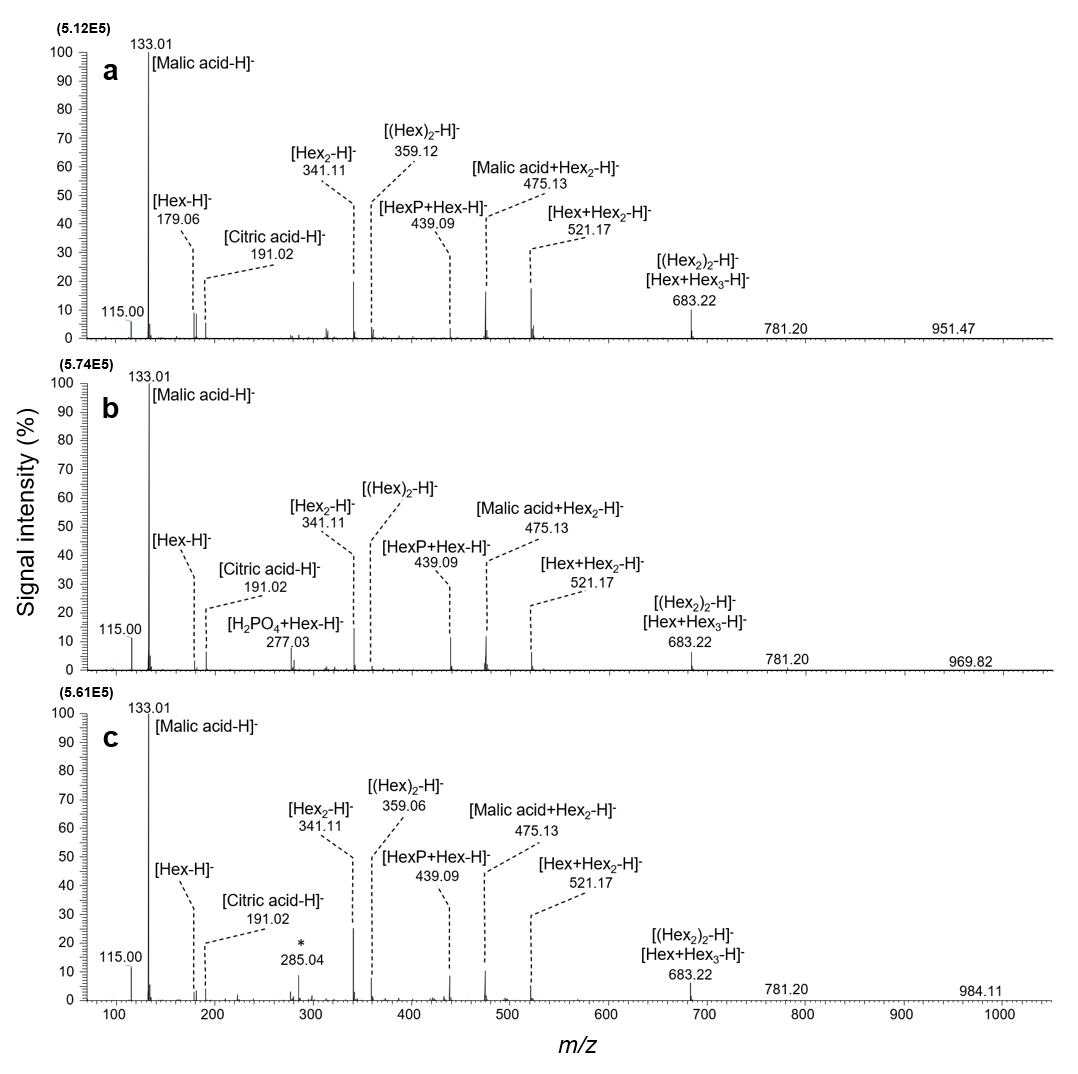


**Figure S3-1. PicoPPESI negative ion mode mass spectra obtained from each region tissue extract solution in watercored apple fruit.** Data in normal outer parenchyma (**a**), border (**b**), and watercore (**c**) regions are representative of similar experiments with 4 apples Asterisk markings indicate assigned background peaks from mixture silicone oil+ionic solution filling into the pressure probe capillary. Hex: hexose.

**Figure S3-2**


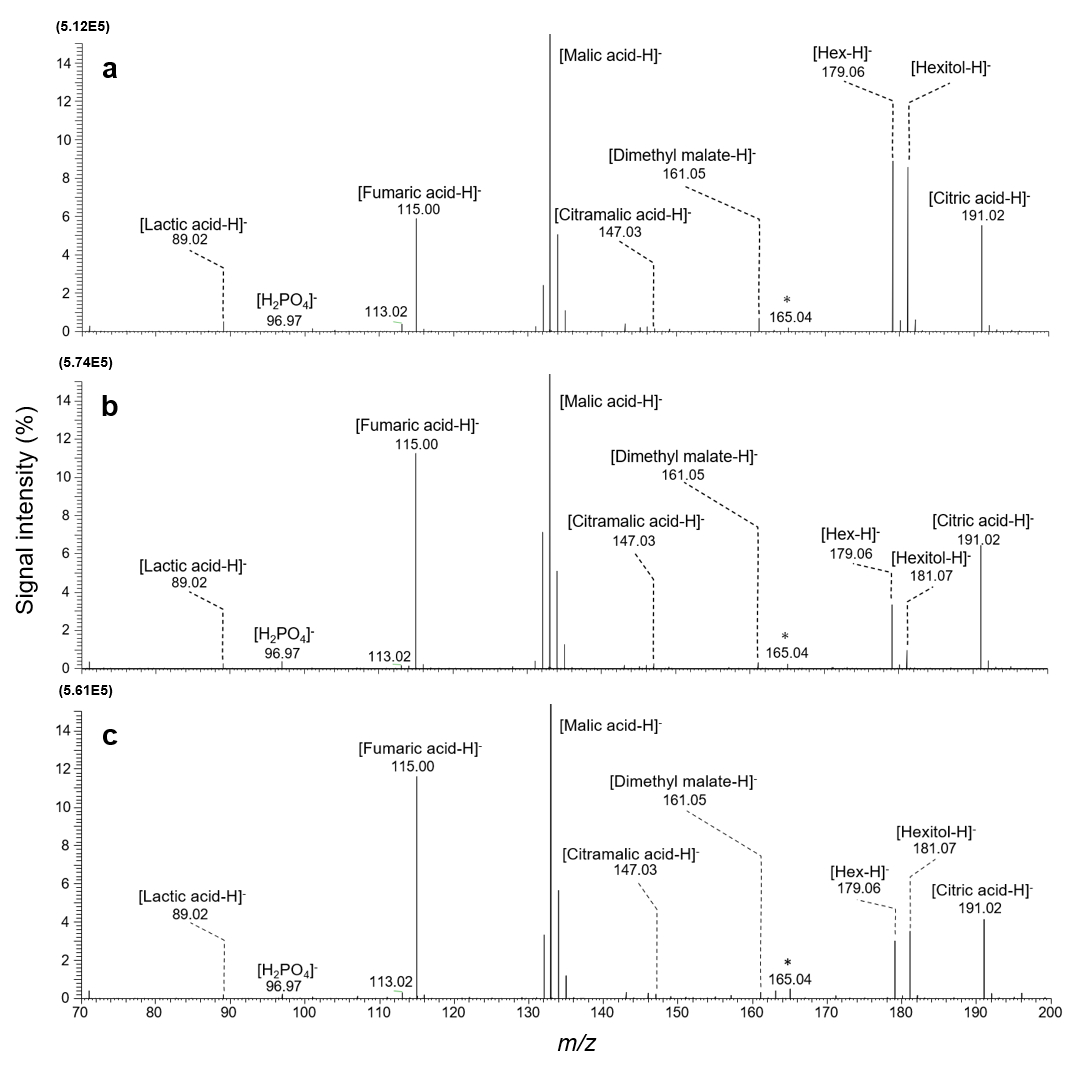


**Figure S3-2. The range of *m/z* 70-200 in picoPPESI negative ion mode mass spectra obtained from each region tissue extract solution in watercored apple fruit.** Data in normal outer parenchyma (**a**), border (**b**), and watercore (**c**) regions are representative of similar experiments with 4 apples Asterisk markings indicate assigned background peaks from mixture silicone oil+ionic solution filling into the pressure probe capillary. The full mass spectra are shown in Fig. S3-1. Hex: hexose.

**Figure S3-3.**


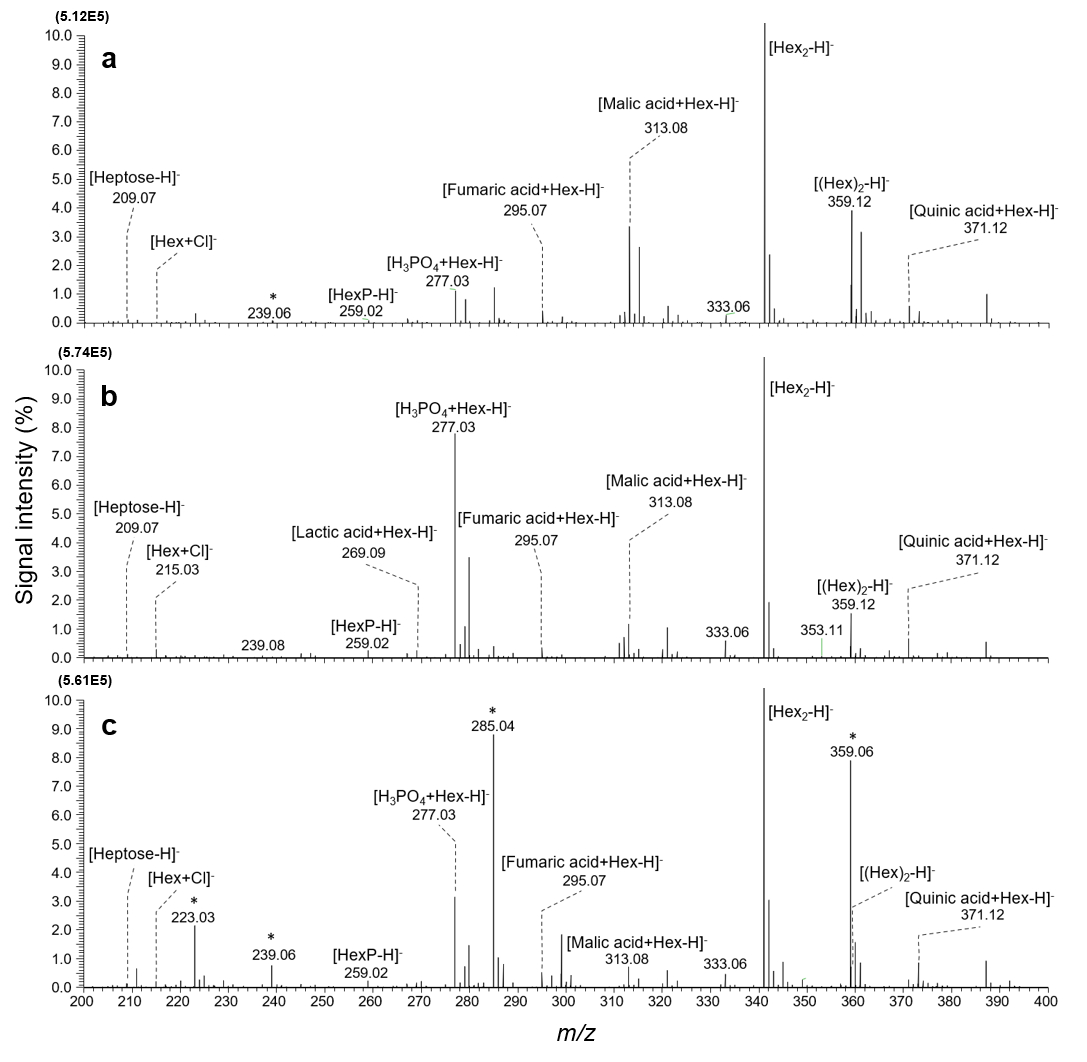


**Figure S3-3. The range of *m/z* 200-400 in picoPPESI negative ion mode mass spectra obtained from each region tissue extract solution in watercored apple fruit.** Data in normal outer parenchyma (**a**), border (**b**), and watercore (**c**) regions are representative of similar experiments with 4 apples Asterisk markings indicate assigned background peaks from mixture silicone oil+ionic solution filling into the pressure probe capillary. The full mass spectra are shown in Fig. S3-1. Hex: hexose; HexP: hexose phosphate.

**Figure S3-4**


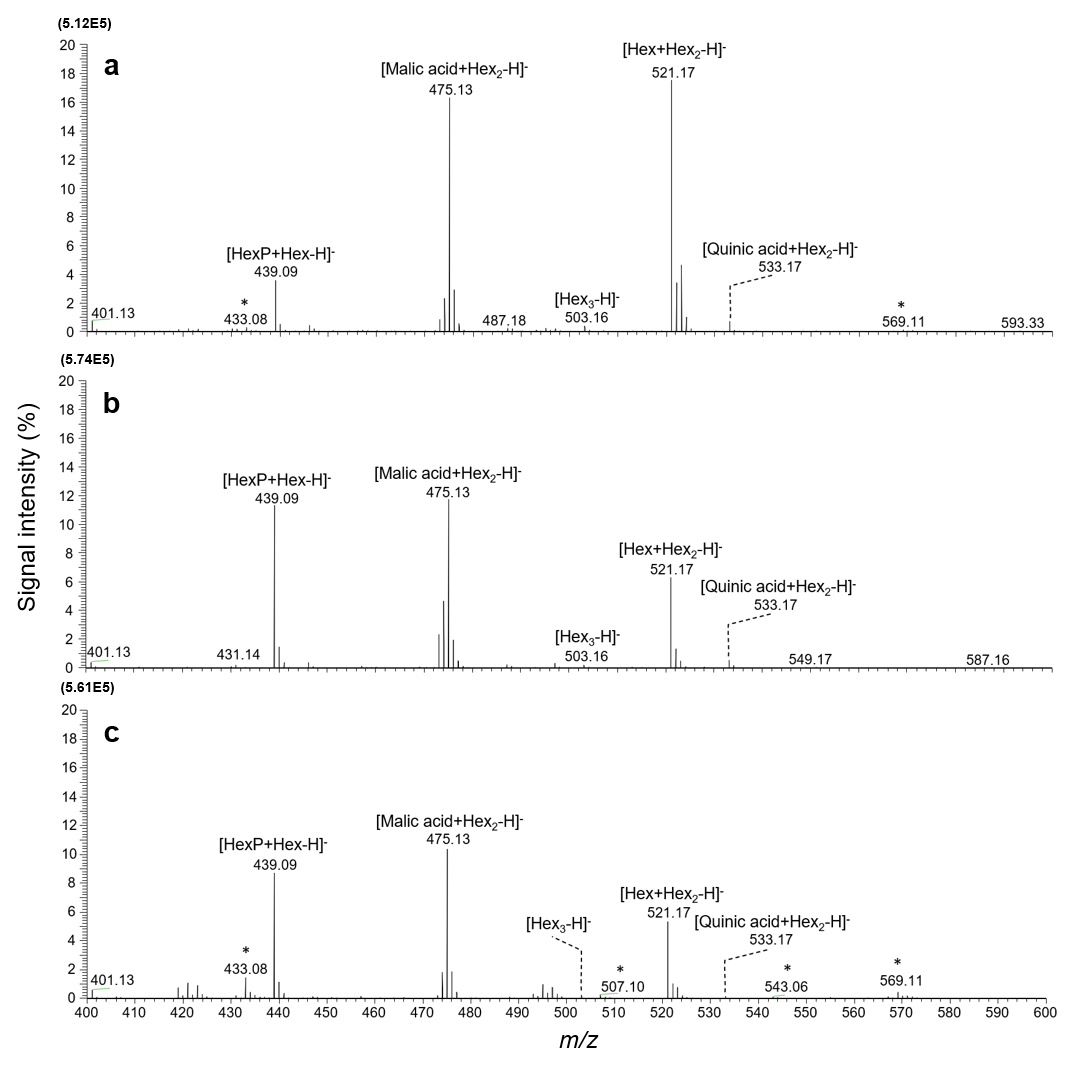


**Figure S3-4. The range of *m/z* 400-600 in picoPPESI negative ion mode mass spectra obtained from each region tissue extract solution in watercored apple fruit.** Data in normal outer parenchyma (**a**), border (**b**), and watercore (**c**) regions are representative of similar experiments with 4 apples Asterisk markings indicate assigned background peaks from mixture silicone oil+ionic solution filling into the pressure probe capillary. The full mass spectra are shown in Fig. S3-1. Hex: hexose.

**Figure S3-5**


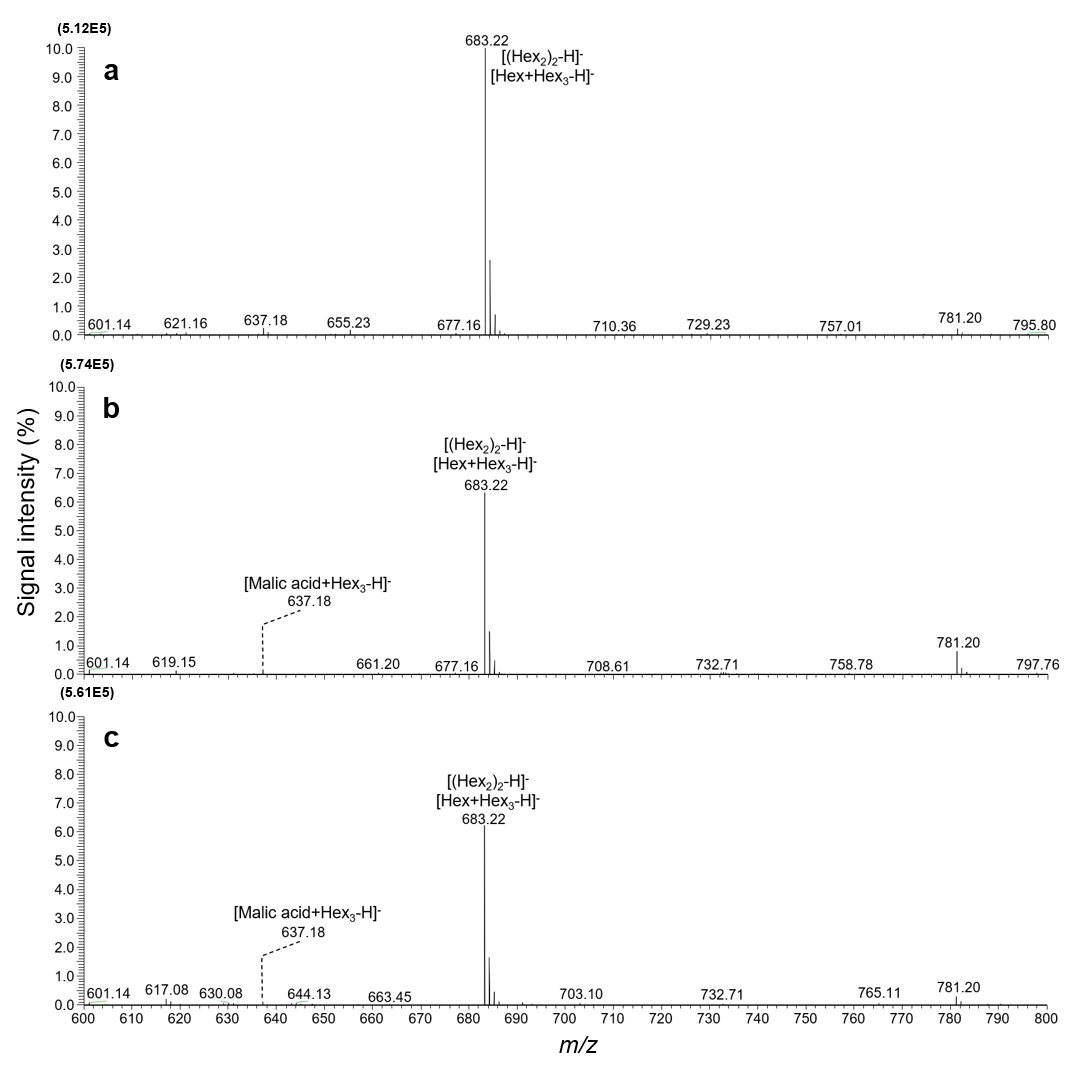


**Figure S3-5. The range of *m/z* 600-800 in picoPPESI negative ion mode mass spectra obtained from each region tissue extract solution in watercored apple fruit.** Data in normal outer parenchyma (**a**), border (**b**), and watercore (**c**) regions are representative of similar experiments with 4 apples Asterisk markings indicate assigned background peaks from mixture silicone oil+ionic solution filling into the pressure probe capillary. The full mass spectra are shown in Fig. S3-1. Hex: hexose.

**Figure S3-6**


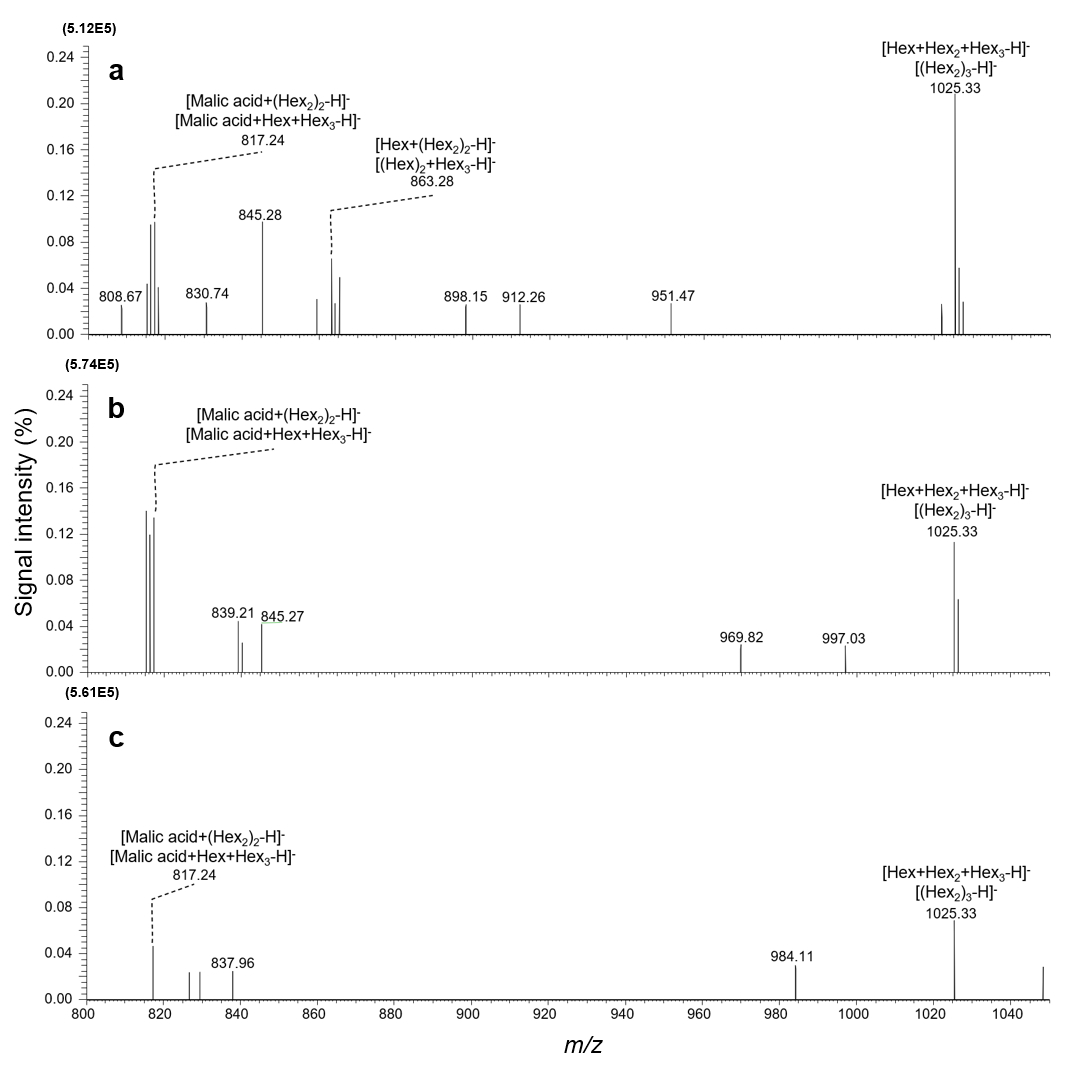


**Figure S3-6. The range of *m/z* 800-1050 in picoPPESI negative ion mode mass spectra obtained from each region tissue extract solution in watercored apple fruit.** Data in normal outer parenchyma (**a**), border (**b**), and watercore (**c**) regions are representative of similar experiments with 4 apples Asterisk markings indicate assigned background peaks from mixture silicone oil+ionic solution filling into the pressure probe capillary. The full mass spectra are shown in Fig. S3-1. Hex: hexose.

**Figure S4-1.**


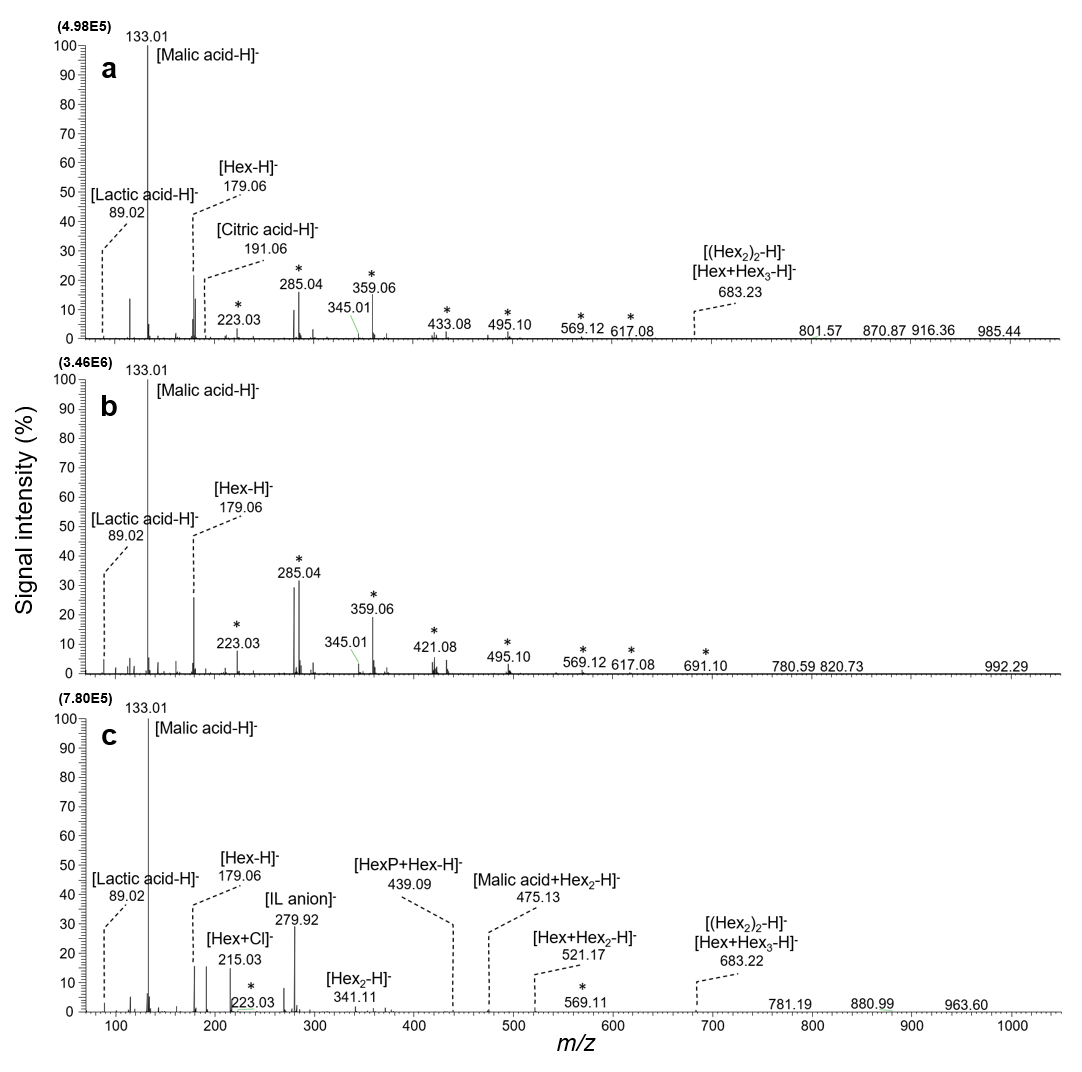


**Figure S4-1. PicoPPESI negative ion mode mass spectra obtained from mesocarp cells located at the outer parenchyma (a) and two regions corresponding to the border (b) and watercore (c) in normal apple fruit.** Data in the regions are representative of similar experiments with 7 apples. Asterisk markings indicate assigned background peaks from mixture silicone oil+ionic solution filling into the pressure probe capillary. Hex: hexose; HexP: hexose phosphate; IL: ionic liquid.

**Figure S4-2**


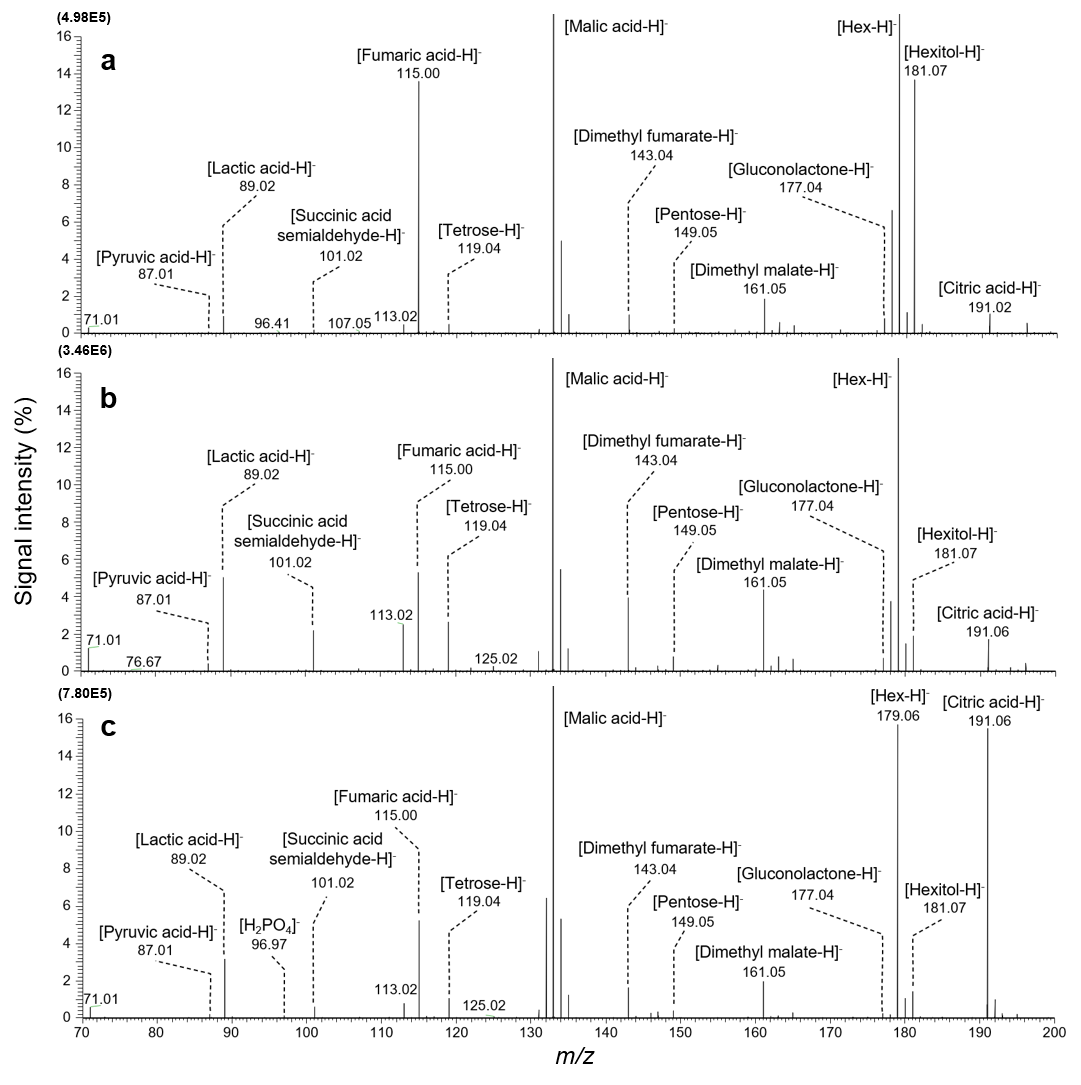


**Figure S4-2. The range of *m/z* 70-200 in picoPPESI negative ion mode mass spectra obtained from mesocarp cells located at the outer parenchyma (a) and two regions corresponding to the border (b) and watercore (c) in normal apple fruit.** Data in the regions are representative of similar experiments with 7 apples Asterisk markings indicate assigned background peaks from mixture silicone oil+ionic solution filling into the pressure probe capillary. The full mass spectra are shown in Fig. S4-1. Hex: hexose.

**Figure S4-3**


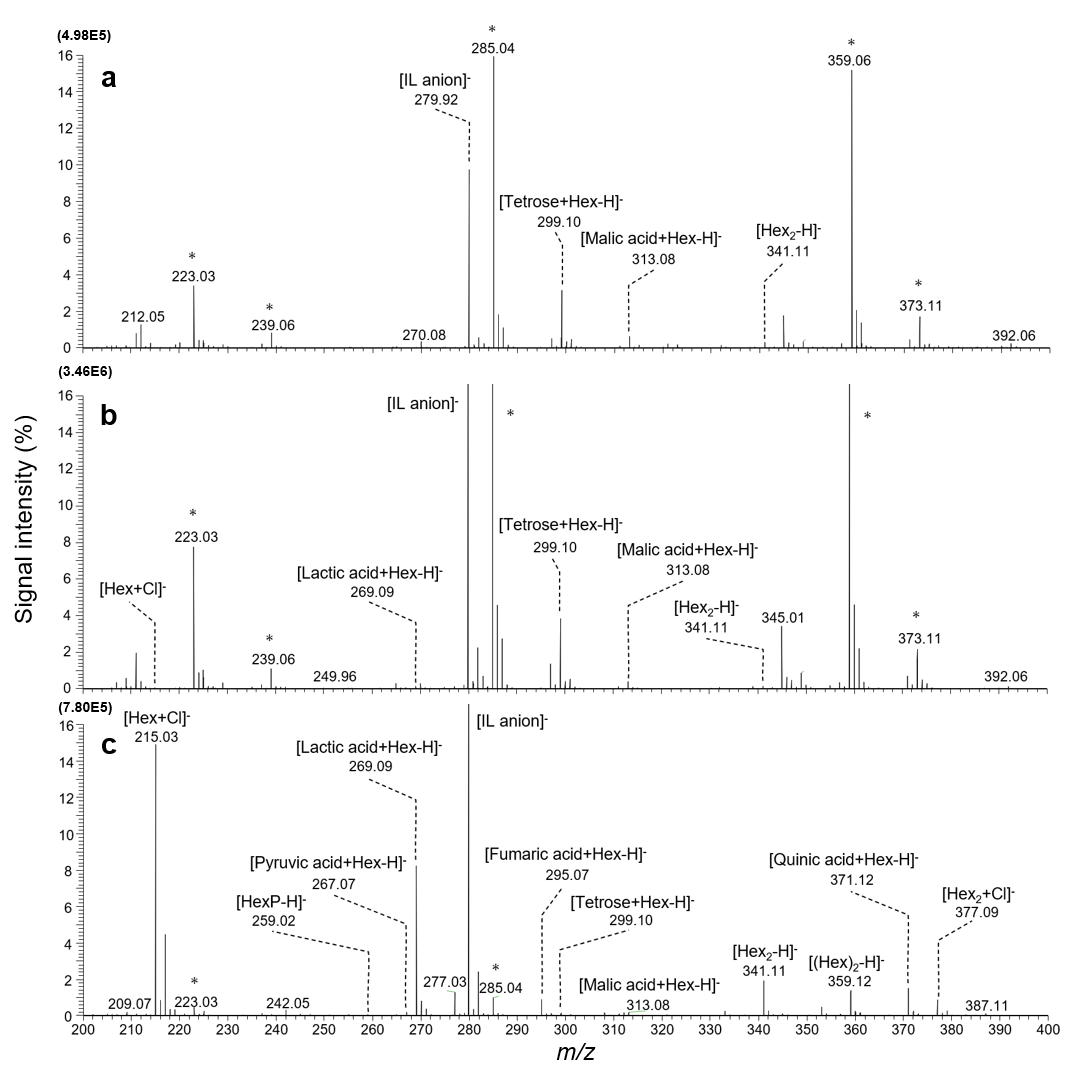


**Figure S4-3. The range of *m/z* 200-400 in picoPPESI negative ion mode mass spectra obtained from mesocarp cells located at the outer parenchyma (a) and two regions corresponding to the border (b) and watercore (c) in normal apple fruit.** Data in the regions are representative of similar experiments with 7 apples. Asterisk markings indicate assigned background peaks from mixture silicone oil+ionic solution filling into the pressure probe capillary. The full mass spectra are shown in Fig. S4-1. IL: ionic liquid.

**Figure S4-4.**


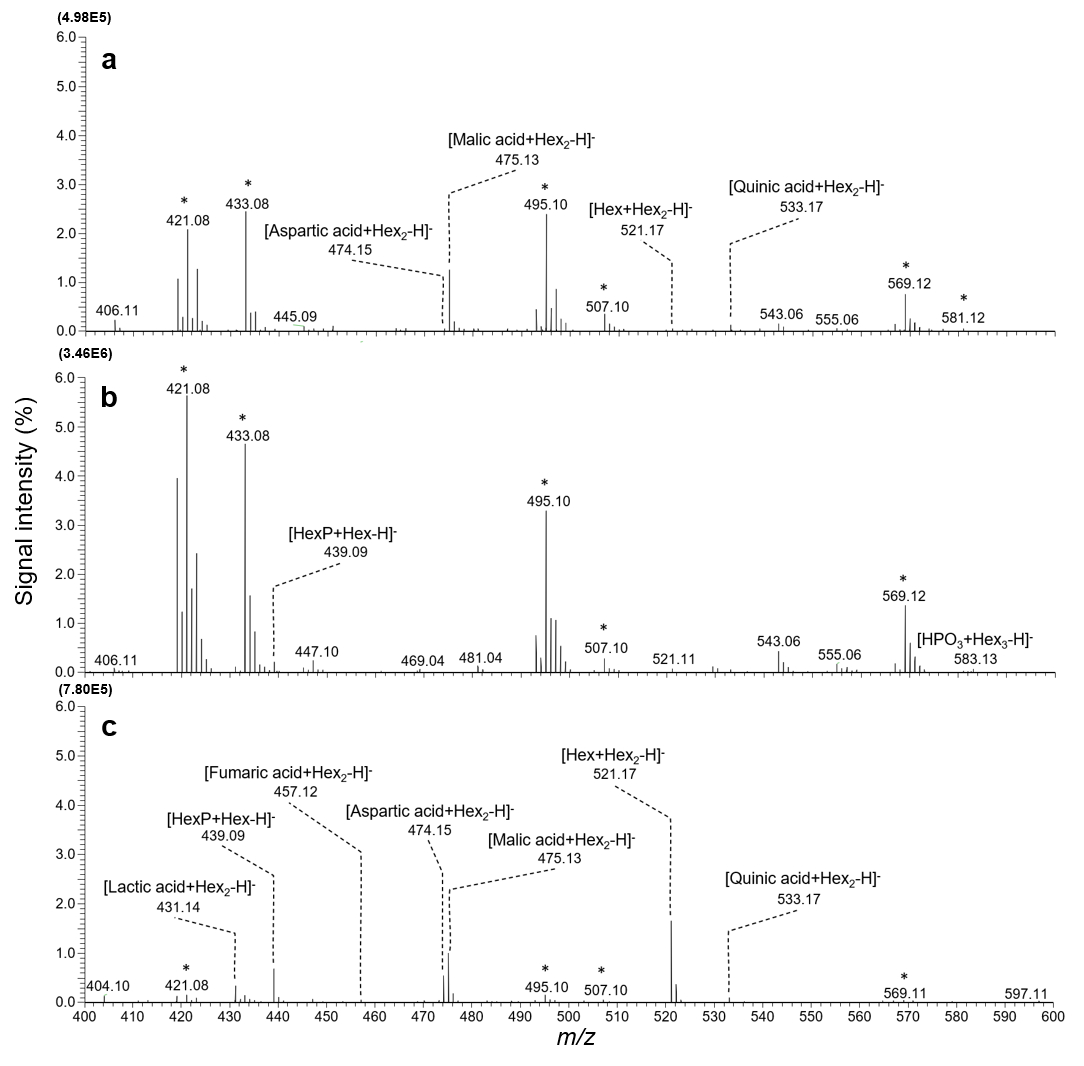


**Figure S4-4. The range of *m/z* 400-600 in picoPPESI negative ion mode mass spectra obtained from mesocarp cells located at the outer parenchyma (a) and two regions corresponding to the border (b) and watercore (c) in normal apple fruit.** Data in the regions are representative of similar experiments with 7 apples Asterisk markings indicate assigned background peaks from mixture silicone oil+ionic solution filling into the pressure probe capillary. The full mass spectra are shown in Fig. S4-1. Hex: hexose.

**Figure S4-5.**


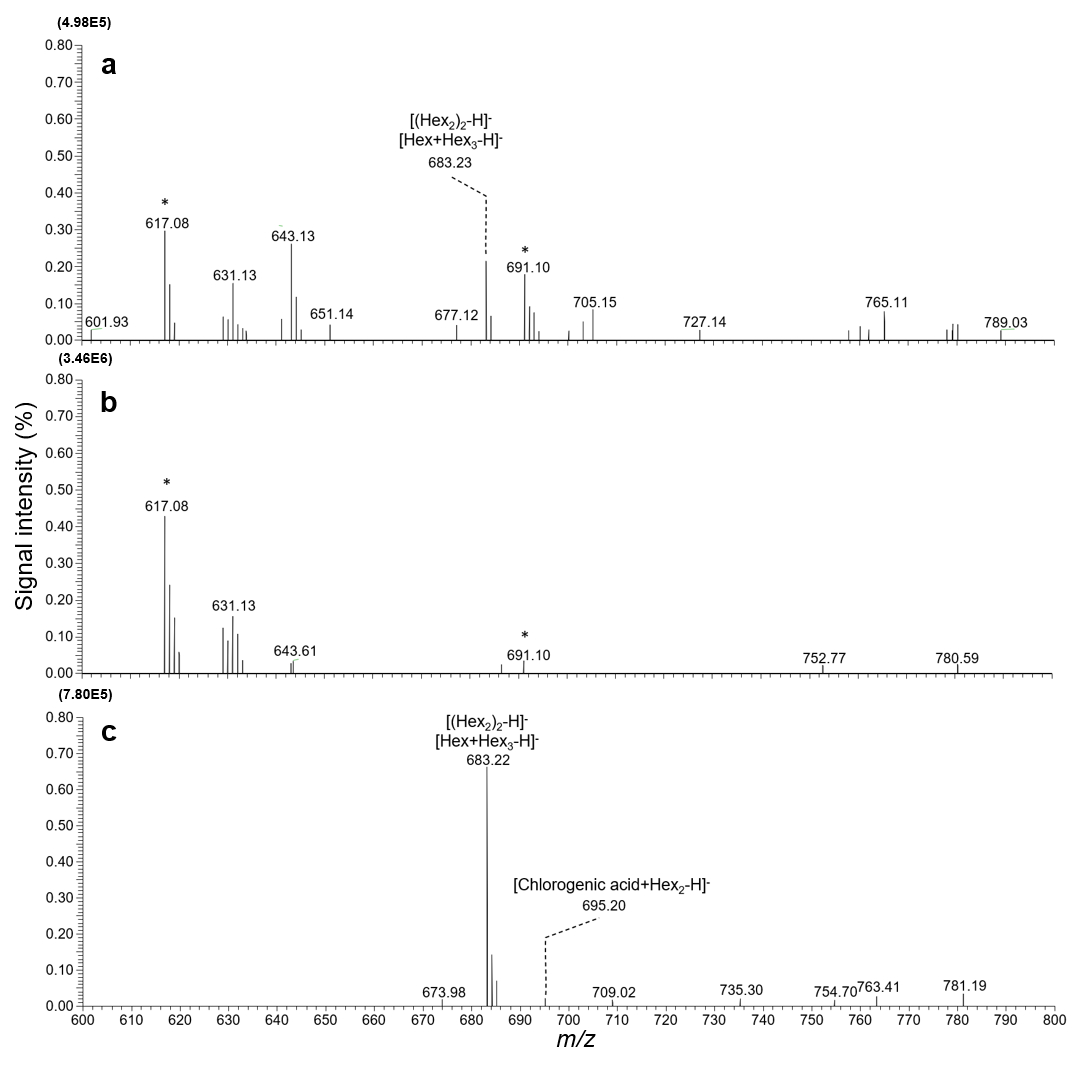


**Figure S4-5. The range of *m/z* 600-800 in picoPPESI negative ion mode mass spectra obtained from mesocarp cells located at the outer parenchyma (a) and two regions corresponding to the border (b) and watercore (c) in normal apple fruit.** Data in the regions are representative of similar experiments with 7 apples. Asterisk markings indicate assigned background peaks from mixture silicone oil+ionic solution filling into the pressure probe capillary. The full mass spectra are shown in Fig. S4-1. Hex: hexose.

**Figure S4-6**


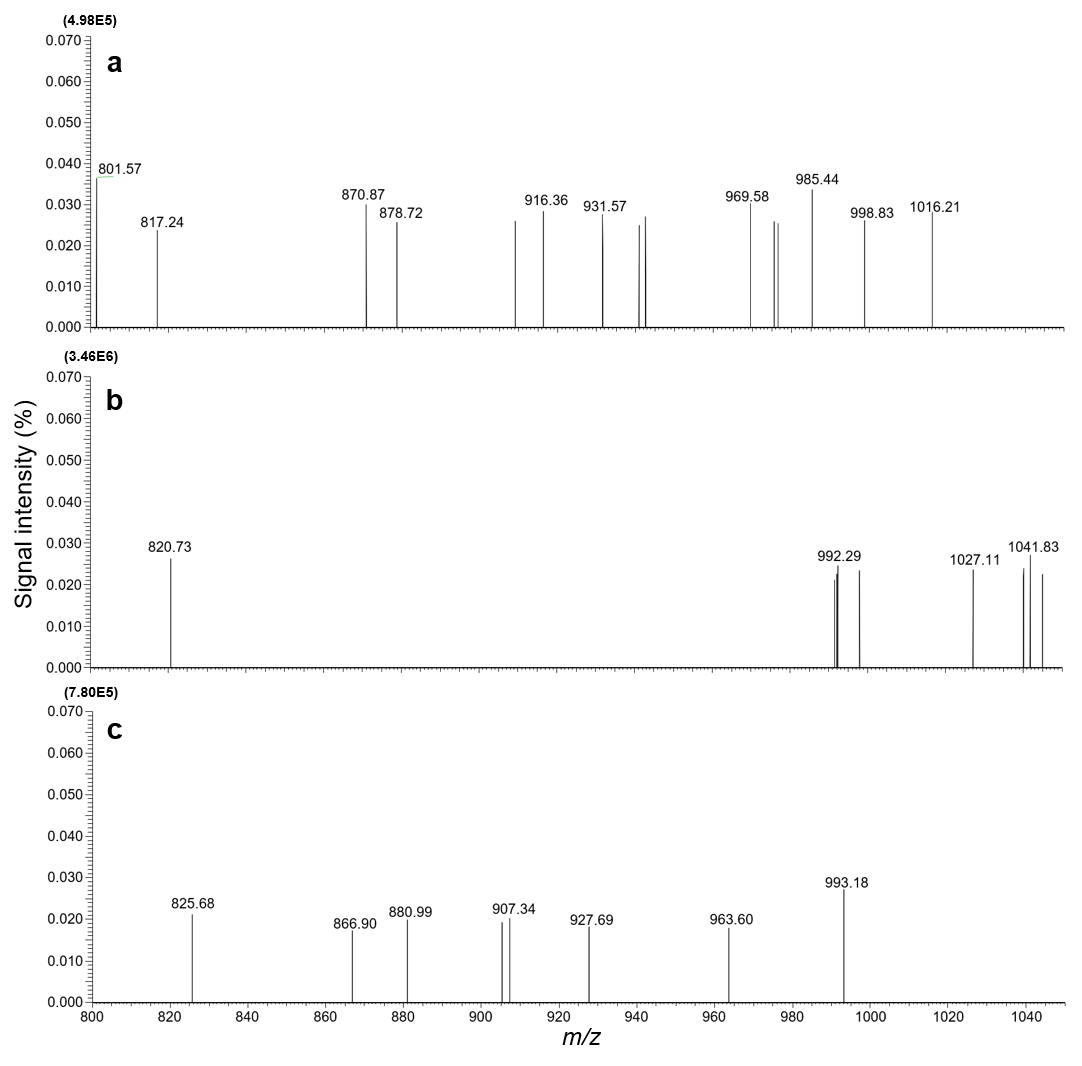


**Figure S4-6. The range of *m/z* 800-1050 in picoPPESI negative ion mode mass spectra obtained from mesocarp cells located at the outer parenchyma (a) and two regions corresponding to the border (b) and watercore (c) in normal apple fruit.** Data in the regions are representative of similar experiments with 7 apples. Asterisk markings indicate assigned background peaks from mixture silicone oil+ionic solution filling into the pressure probe capillary. The full mass spectra are shown in Fig. S4-1.

**Figure S5-1**


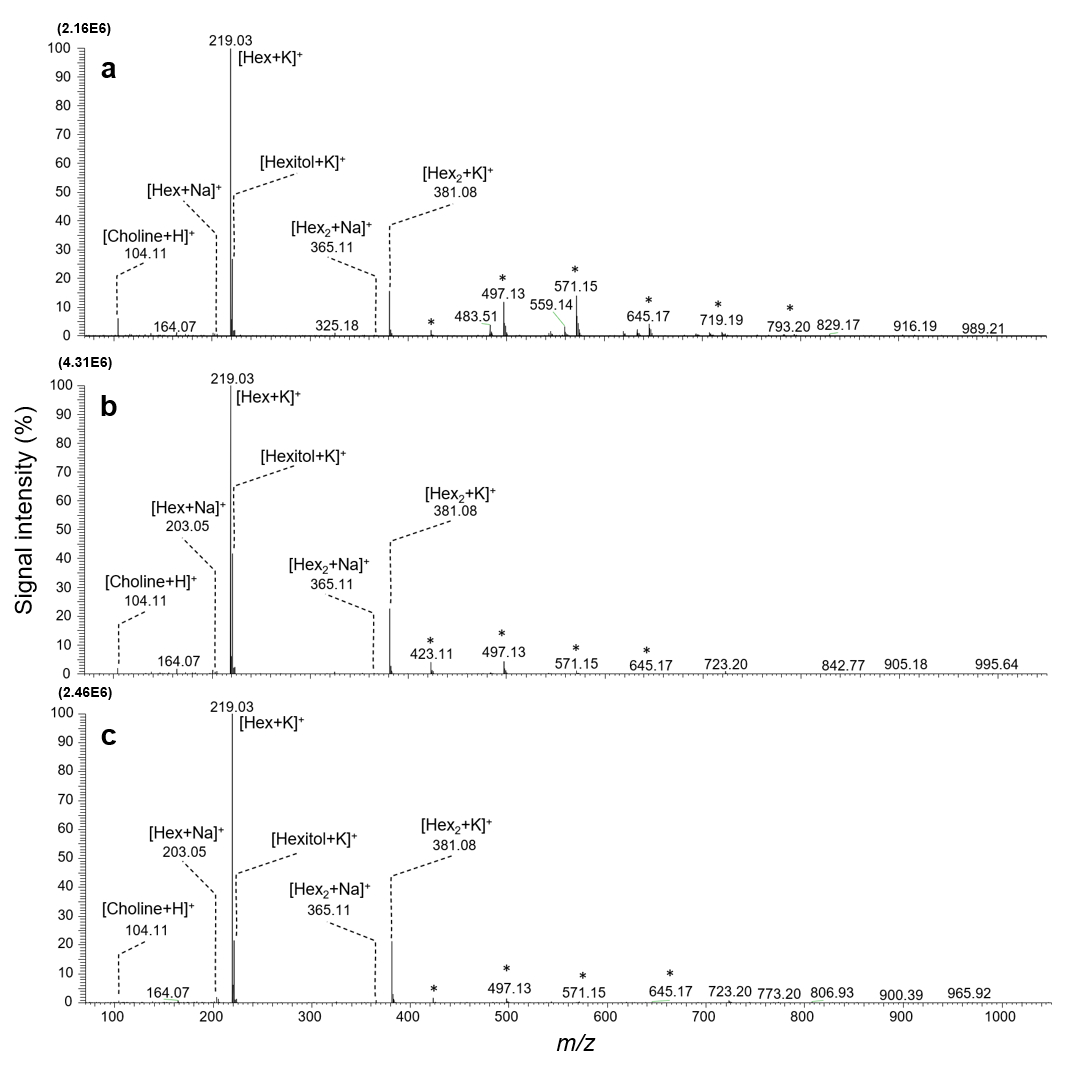


**Figure S5-1. PicoPPESI positive ion mode mass spectra obtained from each region tissue extract solution in watercored apple fruit.** Data in normal outer parenchyma (**a**), border (**b**), and watercore (**c**) regions are representative of similar experiments with 4 apples Asterisk markings indicate assigned background peaks from mixture silicone oil+ionic solution filling into the pressure probe capillary. Hex: hexose.

**Figure S5-2**


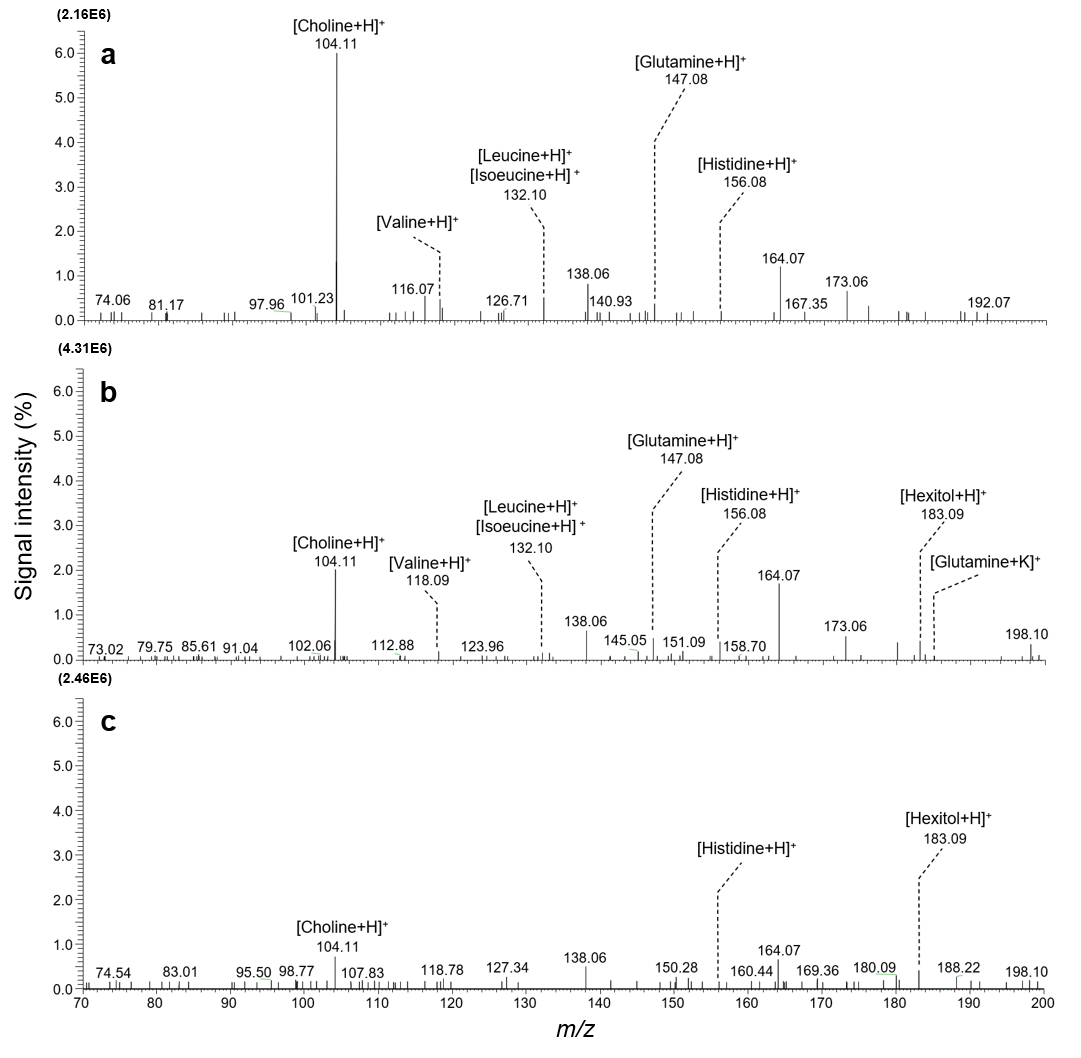


**Figure S5-2. The range of *m/z* 70-200 in picoPPESI positive ion mode mass spectra obtained from each region tissue extract solution in watercored apple fruit.** Data in normal outer parenchyma (**a**), border (**b**), and watercore (**c**) regions are representative of similar experiments with 4 apples. Asterisk markings indicate assigned background peaks from mixture silicone oil+ionic solution filling into the pressure probe capillary. The full mass spectra are shown in Fig. S5-1.

**Figure S5-3**


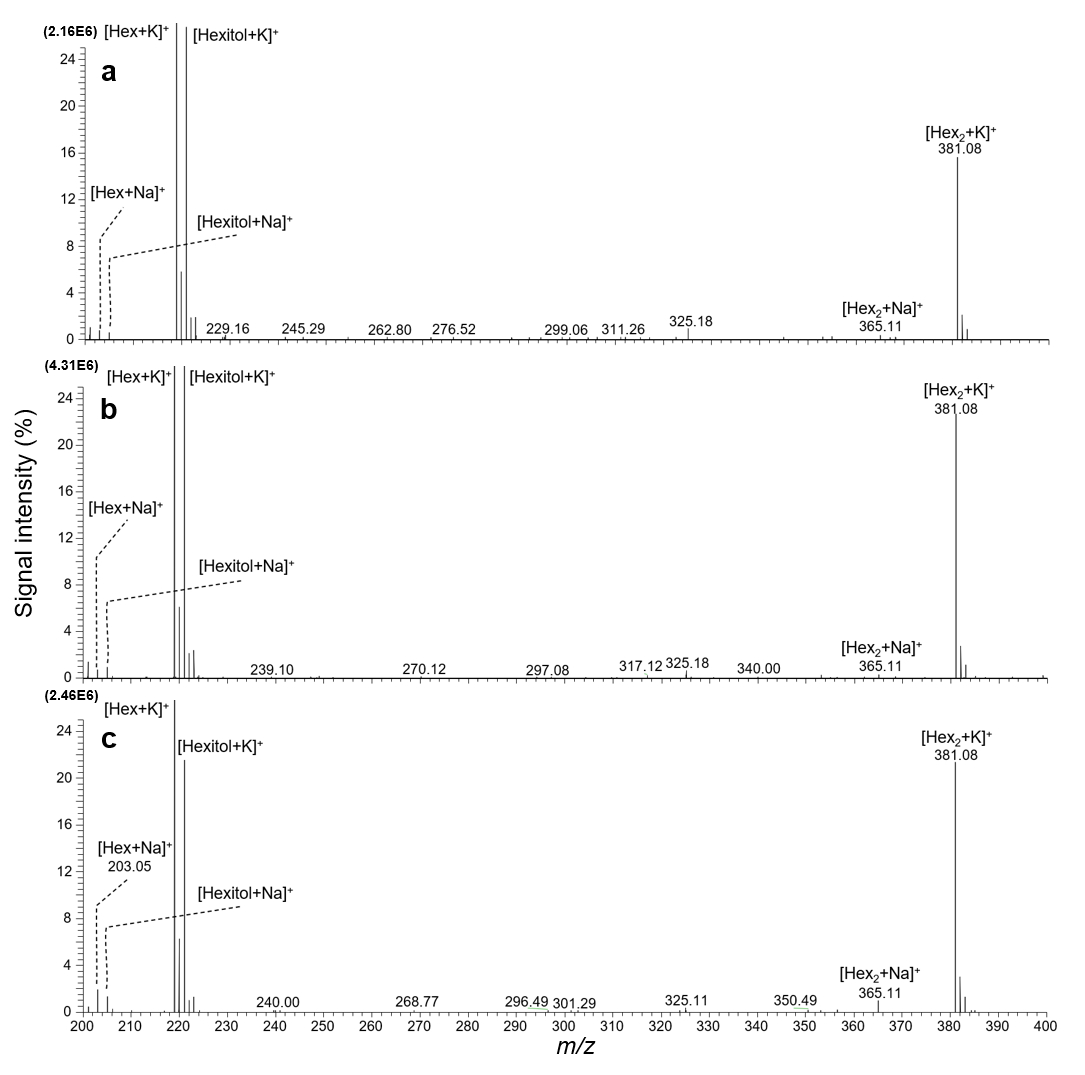


**Figure S5-3. The range of *m/z* 200-400 in picoPPESI positive ion mode mass spectra obtained from each region tissue extract solution in watercored apple fruit.** Data in normal outer parenchyma (**a**), border (**b**), and watercore (**c**) regions are representative of similar experiments with 4 apples. Asterisk markings indicate assigned background peaks from mixture silicone oil+ionic solution filling into the pressure probe capillary. The full mass spectra are shown in Fig. S5-1. Hex: hexose.

**Figure S5-4**


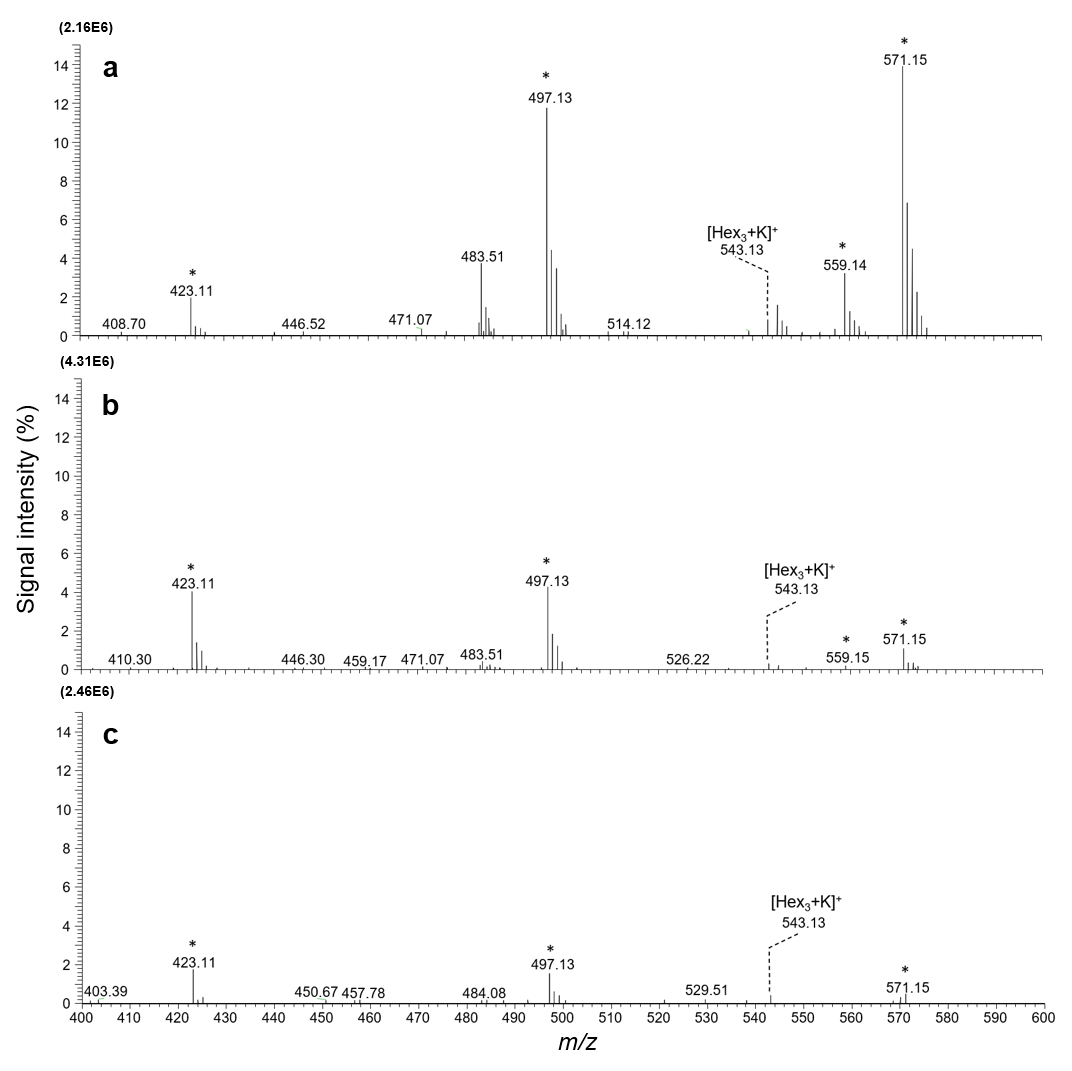


**Figure S5-4. The range of *m/z* 400-600 in picoPPESI positive ion mode mass spectra obtained from each region tissue extract solution in watercored apple fruit.** Data in normal outer parenchyma (**a**), border (**b**), and watercore (**c**) regions are representative of similar experiments with 4 apples. Asterisk markings indicate assigned background peaks from mixture silicone oil+ionic solution filling into the pressure probe capillary. The full mass spectra are shown in Fig. S5-1. Hex: hexose.

**Figure S5-5**


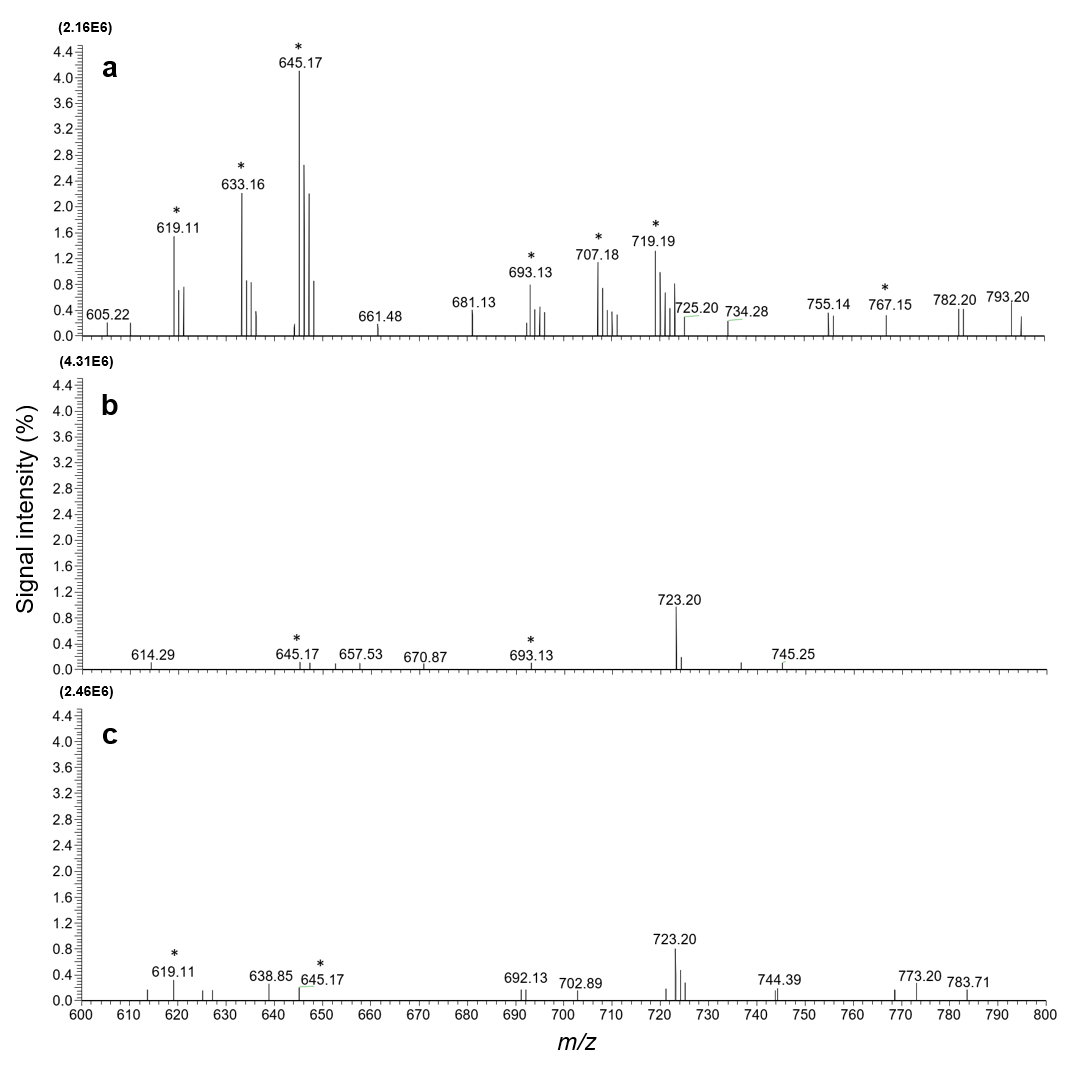


**Figure S5-5. The range of *m/z* 600-800 in picoPPESI positive ion mode mass spectra obtained from each region tissue extract solution in watercored apple fruit.** Data in normal outer parenchyma (**a**), border (**b**), and watercore (**c**) regions are representative of similar experiments with 4 apples. Asterisk markings indicate assigned background peaks from mixture silicone oil+ionic solution filling into the pressure probe capillary. The full mass spectra are shown in Fig. S5-1.

**Figure S5-6**


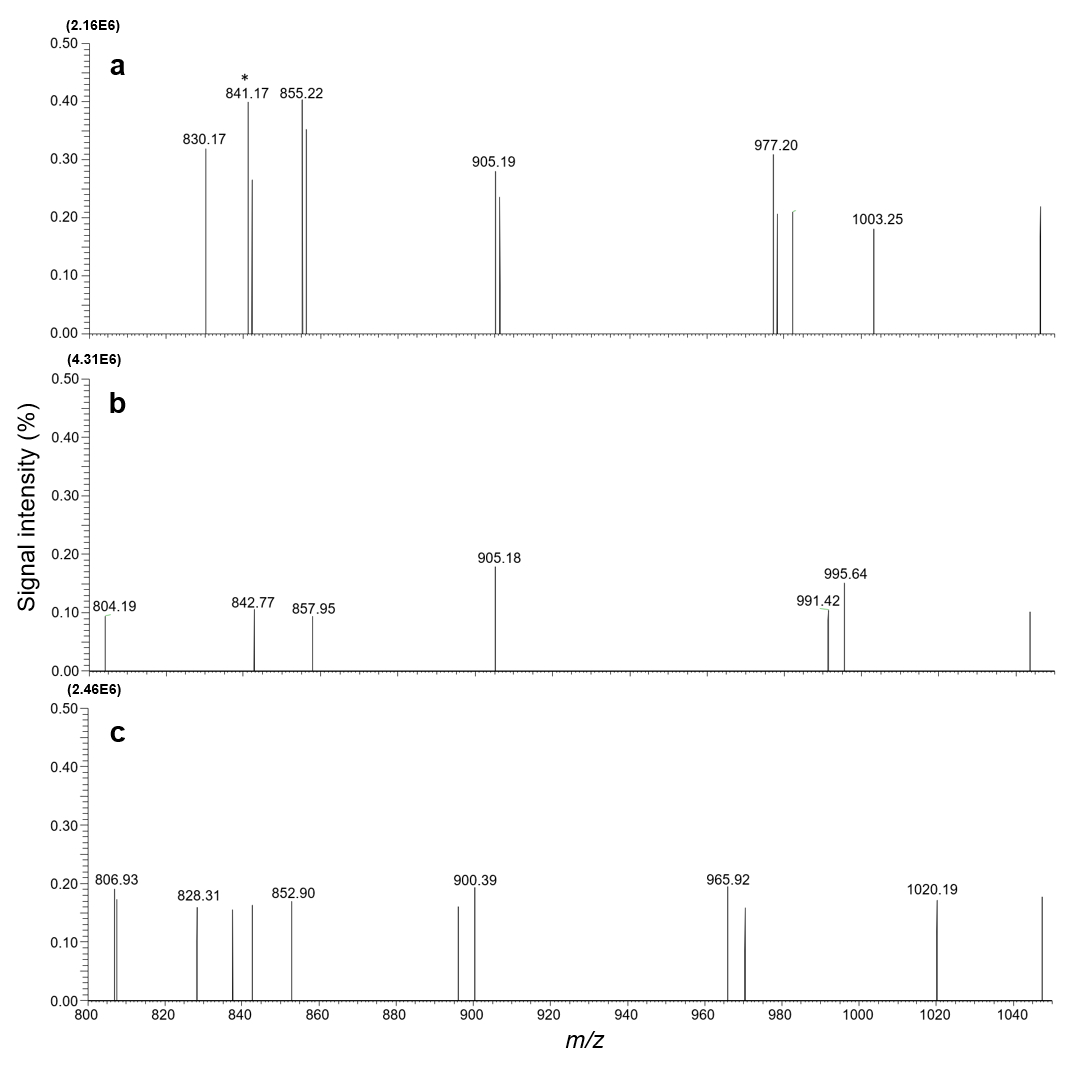


**Figure S5-6. The range of *m/z* 800-1050 in picoPPESI positive ion mode mass spectra obtained from each region tissue extract solution in watercored apple fruit.** Data in normal outer parenchyma (**a**), border (**b**), and watercore (**c**) regions are representative of similar experiments with 4 apples. Asterisk markings indicate assigned background peaks from mixture silicone oil+ionic solution filling into the pressure probe capillary. The full mass spectra are shown in Fig. S5-1.

**Figure S6-1**


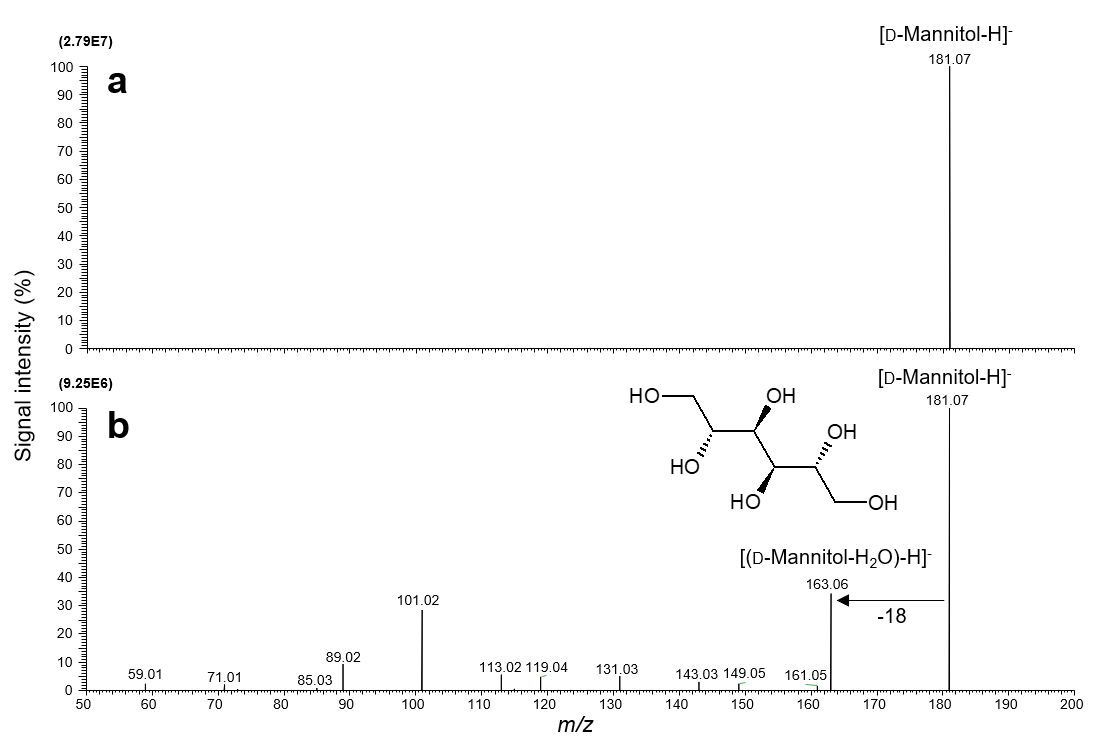


**Figure S6-1. PicoPPESI-MS/MS spectra of a D-Mannitol peak at *m/z* 181.07 from 1.0 mM D-Mannitol standard solution in negative ion mode.** Precursor ion and the selector gate range was *m/z* 181.07±0.5. Normalized collision energy was set to 0% (**a**) or 20% (**b**), and CID fragmentation ions were detected in the Orbitrap at a resolution setting of 240,000. Solvent: water, LC/MS grade (Kanto Kagaku (Tokyo, Japan)); Voltage: -4.0 kV.

**Figure S6-2**


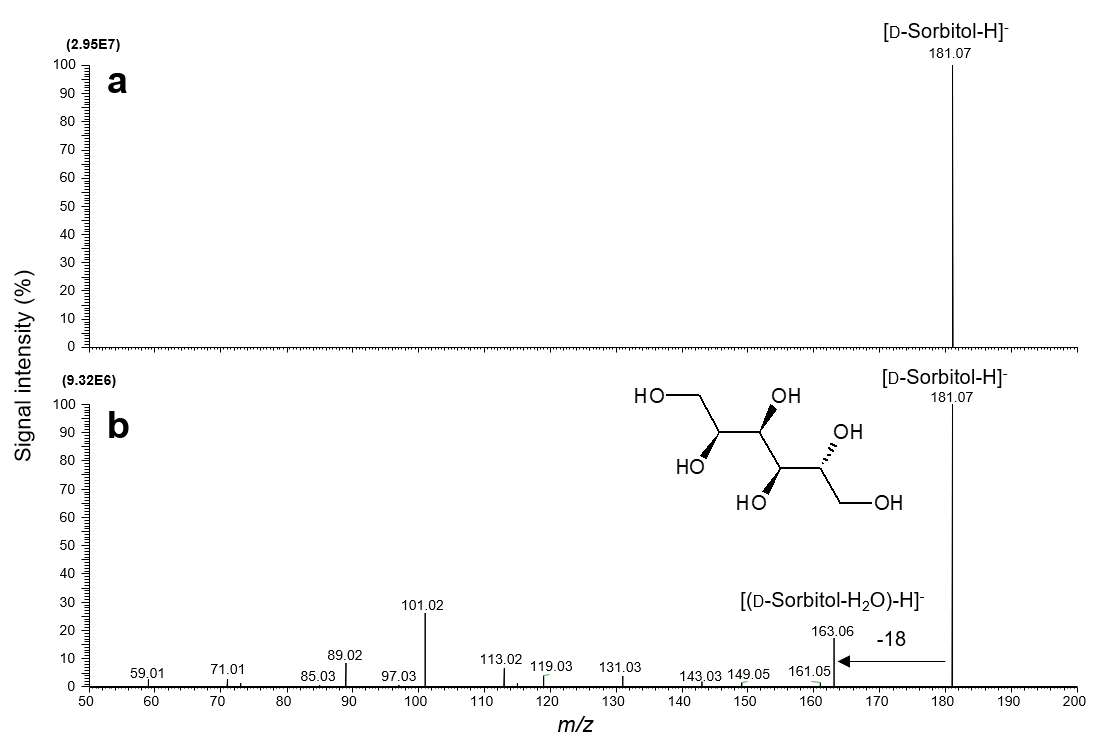


**Figure S6-2. PicoPPESI-MS/MS spectra of a D-Sorbitol peak at *m/z* 181.07 from 1.0 mM D-Sorbitol standard solution in negative ion mode.** Precursor ion and the selector gate range was *m/z* 181.07±0.5. Normalized collision energy was set to 0% (**a**) or 20% (**b**), and CID fragmentation ions were detected in the Orbitrap at a resolution setting of 240,000. Solvent: water, LC/MS grade (Kanto Kagaku (Tokyo, Japan)); Voltage: -4.0 kV.

**Figure S6-3**


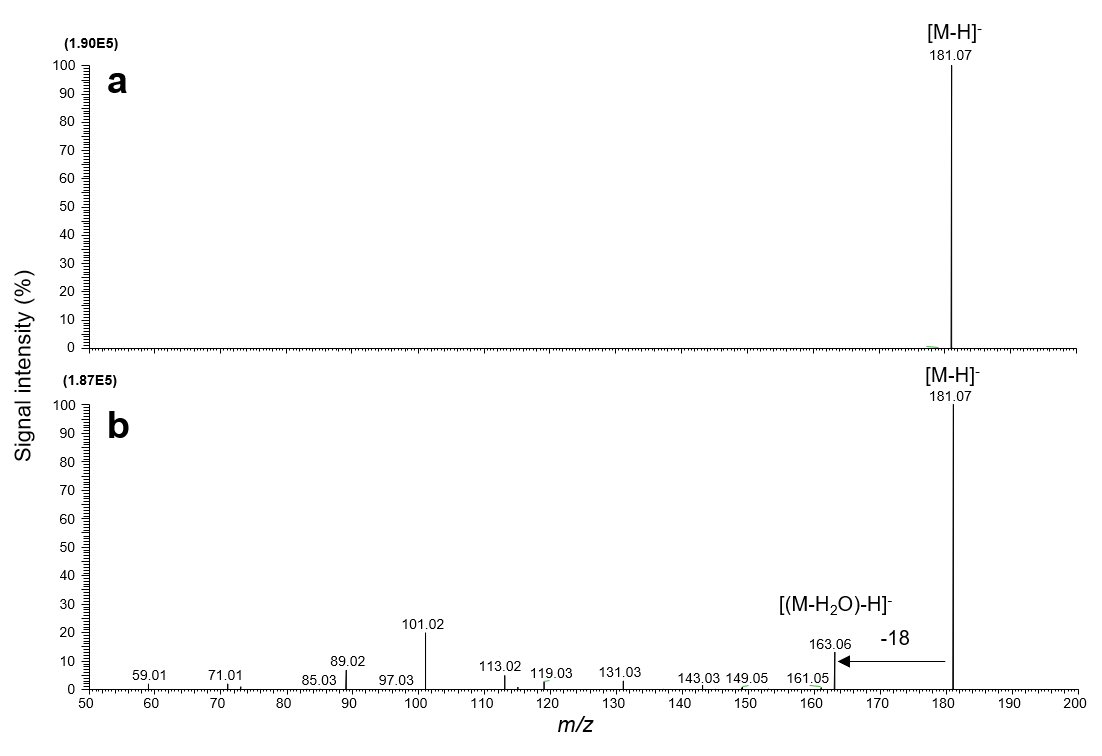


**Figure S6-3. PicoPPESI-MS/MS spectra of a peak at *m/z* 181.07 from apple tissue extract (see Materials and Methods) in negative ion mode.** Precursor ion and the selector gate range was *m/z* 181.07±0.5. Normalized collision energy was set to 0% (**a**) or 20% (**b**), and CID fragmentation ions were detected in the Orbitrap at a resolution setting of 240,000. Voltage: -4.0 kV.

**Figure S7**


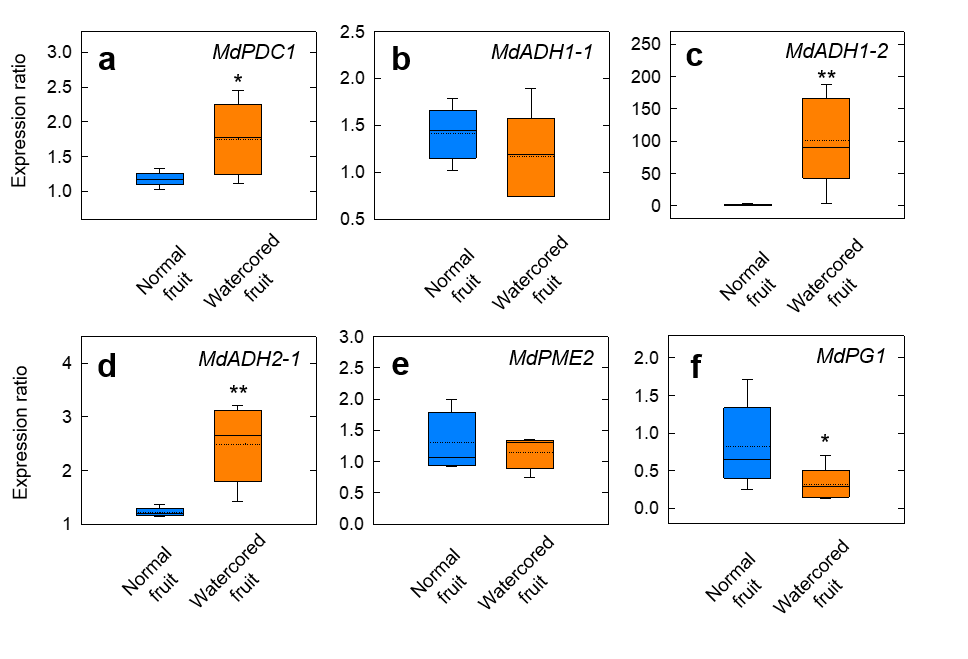


**Figure S7. Difference of gene expression between normal and watercored apple.** The expression ratio indicates the ratio of each tissue located at the two regions corresponding to the normal outer parenchyma and watercore region in normal and watercored apple fruit. The data were obtained from 5 apple fruit. The *p*-value at the 0.15 and 0.05 probability levels indicated with * and **, determined using Wilcoxon rank sum test.

**Figure S8**


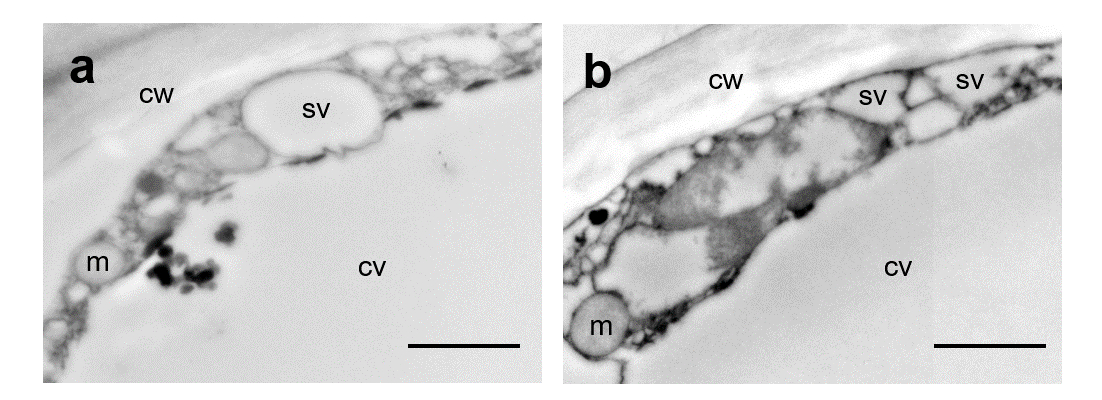


**Figure S8. Ultrastructure in the watercore region of watercored apples. TEM images of the cells in normal outer** **parenchyma (a) and watercore regions (b) in the fruit.** cv: central vacuole; cw: cell wall; m: mitochondria; sv, small vacuole-like structure. Bars = 1 μm.

**Figure S9**

**
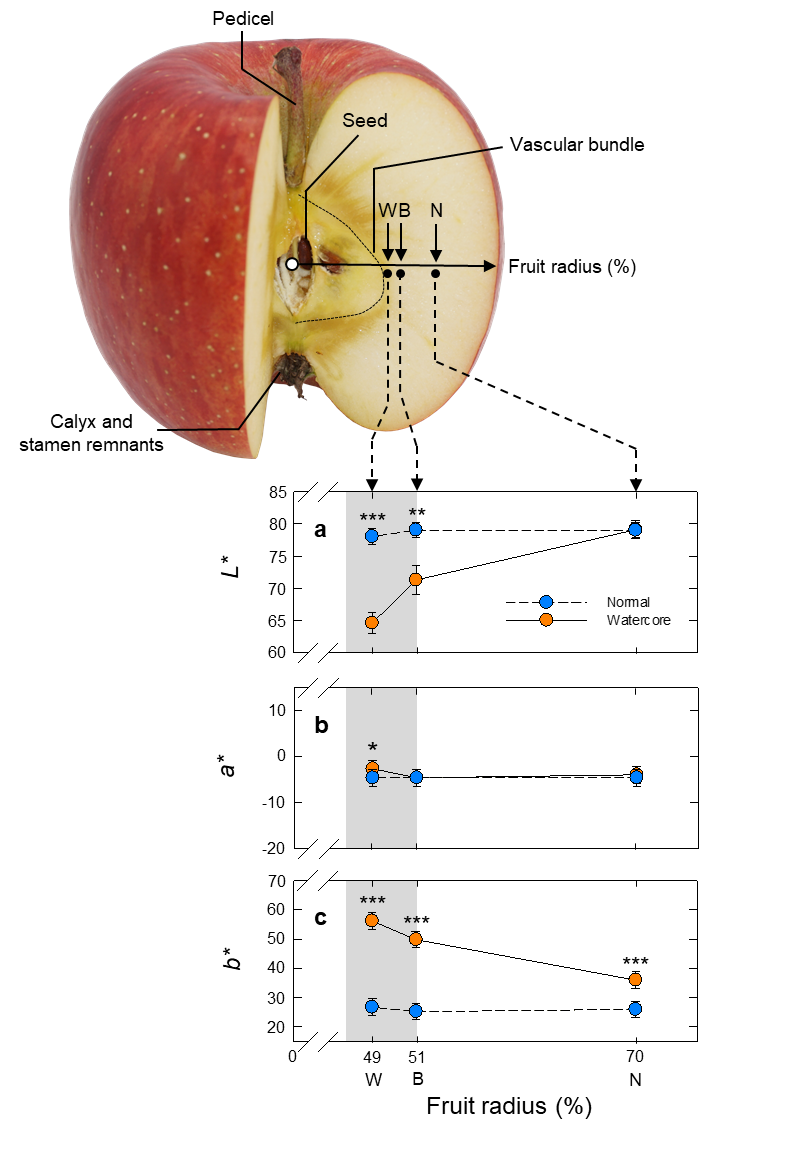
**

**Figure S9. *L** (a), *a** (b), and *b** (c) of colour profiles expressed in *L*a*b** colour space in each position in the fruit (corresponding to watercore region (W), border (B), and normal outer parenchyma (N) regions) plotted as a function of radius (%) in normal or watercored apple fruit.** The data are the means±SE of 7-10 apple fruit. Significant difference at the 0.05, 0.01, and 0.001 probability levels by *t*-test is indicated by *, **, and ***, respectively. Orange and blue circles indicate watercored and normal fruit, respectively.

**Figure S10**


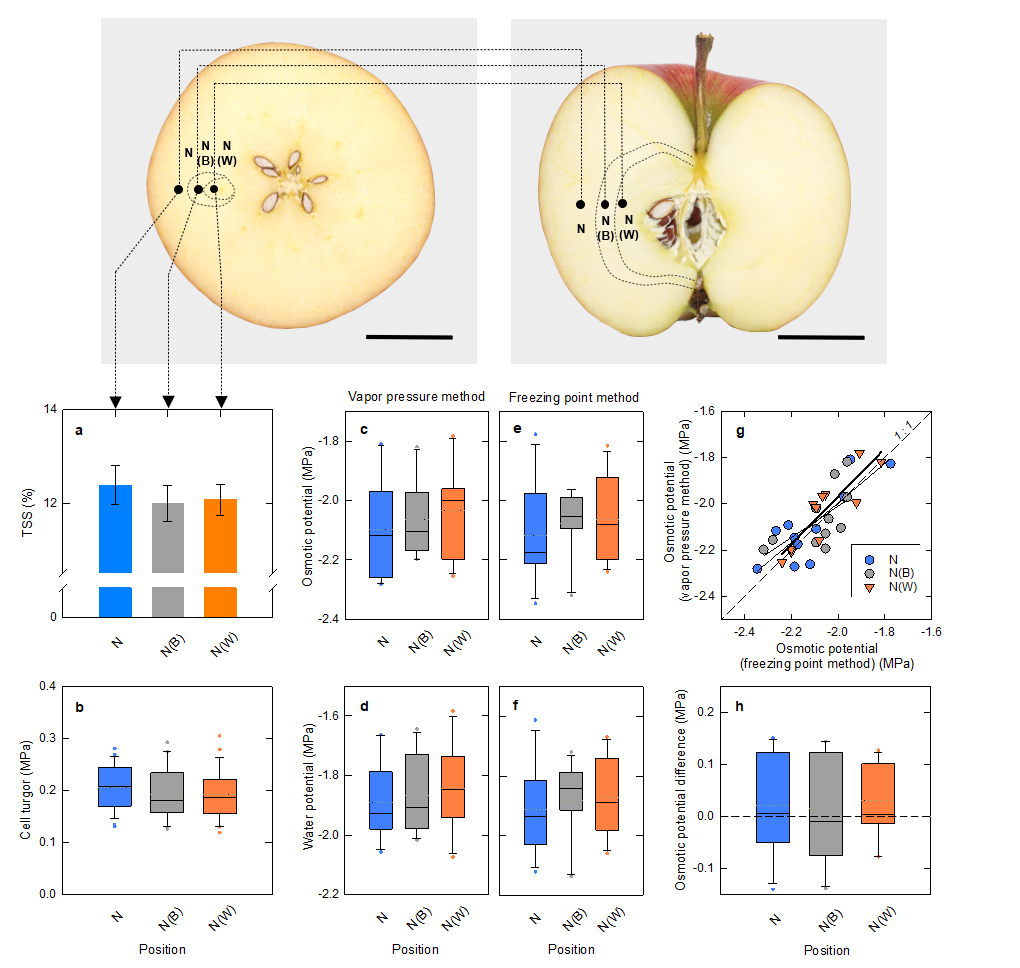


**Figure S10. Water relations in each tissue located at the outer parenchyma (N) and two regions corresponding to the border (N(B)) and watercore (N(W)) in normal apple fruit.** Total soluble solids (TSS) (**a**), cell turgor (**b**), osmotic potential determined by vapor pressure method (**c**), vapor pressure method-based calculated water potential (**d**), osmotic potential determined by freezing point method (**e**), vapor pressure method-based calculated water potential (**f**) in each tissue in the normal apple fruit. The osmotic potential determined by the vapor pressure method was plotted against the osmotic potential determined by the freezing point method in each tissue (**g**). The osmotic potential differences in each position were shown in **h**. The data in **a**, **c**-**f**, and **h** indicate the means±SE of 11 tissues collected from 7 fruit in each treatment. The data in **b** indicate the means±SE of 23-26 cells collected from 7 fruit in each treatment. Different letters indicate a significant difference (Tukey–Kramer test, *p*<0.05). The dashed line in **g** indicates a 1:1 line. Bars = 5 cm.

**Figure S11**


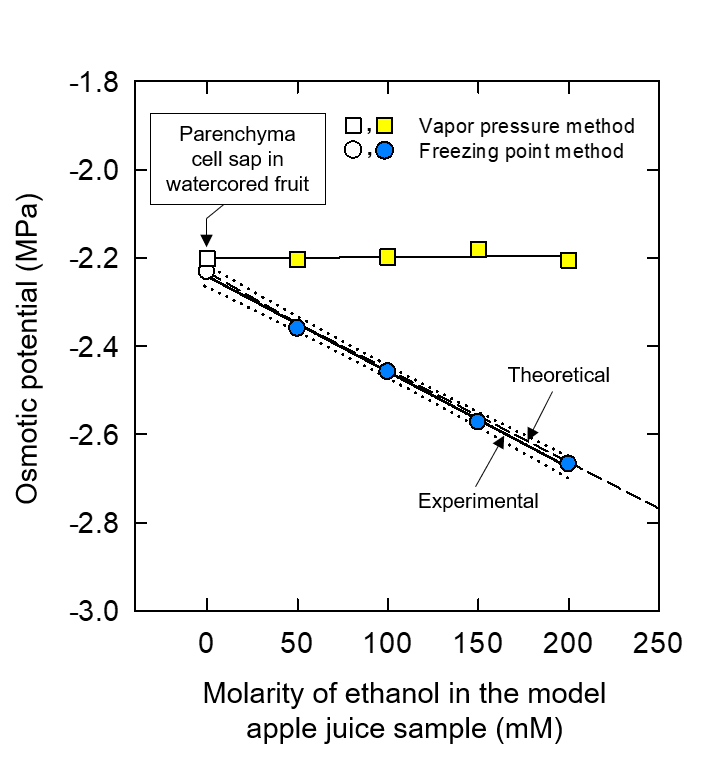


**Figure S11. Osmotic potentials determined with vapor pressure and freezing point methods as a function of molarity of ethanol in the model apple juice sample.** Parenchyma cell sap from watercored fruit were mixed and centrifuged. Aliquots of the supernatant were used to produce 4 model juice samples, adding 4 different concentrations of ethanol each. The regression line between molarity of ethanol in the model apple juice sample (*x*) and the osmotic potential determined by using the freezing point method (*y*) is *y*=-0.002*x*-2.24, with R^2^=1.00 (*p*<0.0001). Dotted lines indicate the 95% confidence intervals as calculated from Student’s percentage *t* distribution. The theoretical line was obtained from Wolf et al. (1983)^20^. Empty square and circle, parenchyma cell sap in the cell; yellow squares, vapor pressure method; blue circles Freezing point method.

**Figure S12**


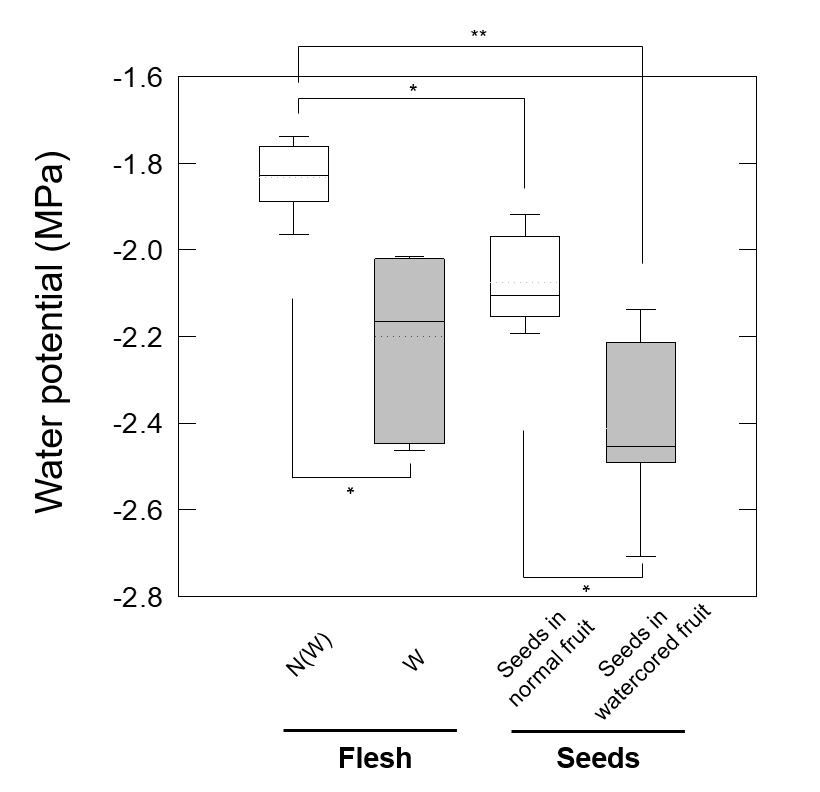


**Figure S12. Water potential gradient from tissue located at the region corresponding to watercore region (N(W)) to seeds in normal apple fruit, and that from watercore region (W) to seeds in watercored apples.** Water potentials in the flesh determined by *in situ* turgor assay combined with freezing point depression method, and seed water potential determined by using isopiestic psychrometers. The data are the means±SE of 6-7 apple fruit; significant difference at the 0.01 and 0.001 probability levels by *t*-test is indicated by * and **, respectively.

**Figure S13**

**
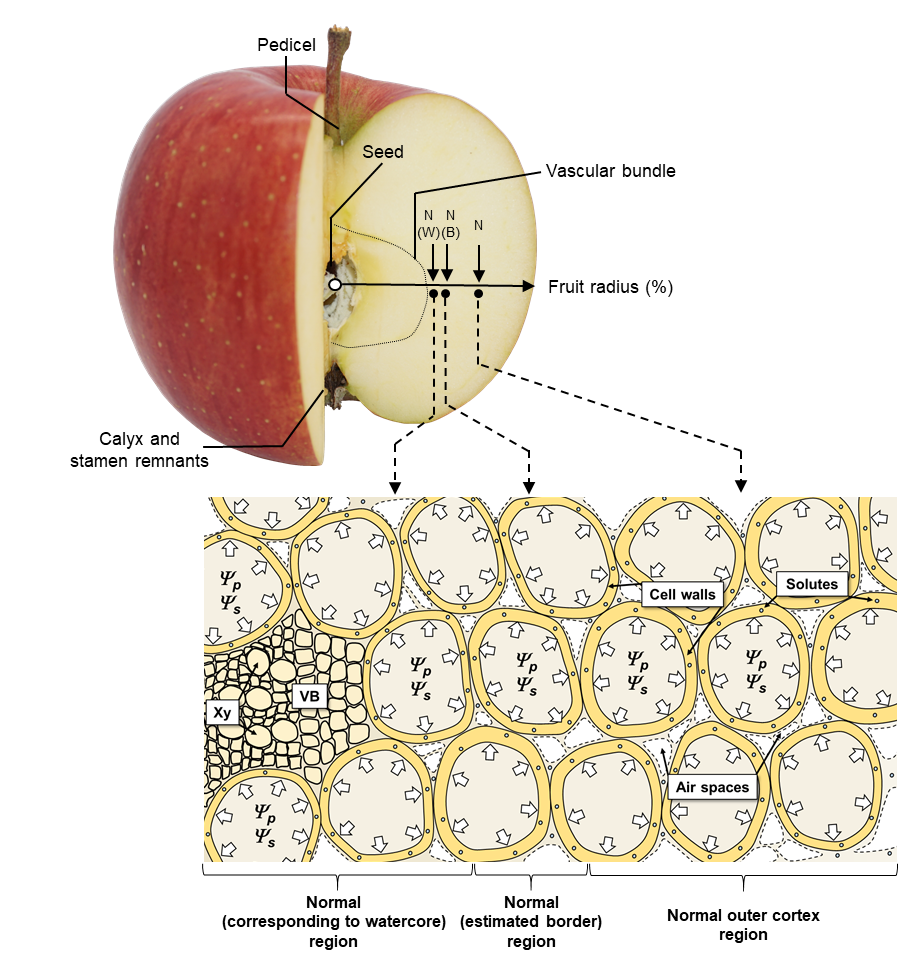
**

**Figure S13. Diagram illustrating water potential equilibrium between normal outer parenchyma (N) and two regions corresponding to the border (N(B)) and watercore region (N(W)) in the normal apple fruit.** There is essentially little water potential gradient established in the normal fruit, contrastingly different from watercored fruit, and cell turgor and osmotic potential were observed to be spatially similar in the flesh. In the cartoon putative apoplastic solutes were drawn and the solutes contained in the cells are removed to simplify. VB: vascular bundle; Xy: xylem.

**Figure S14**


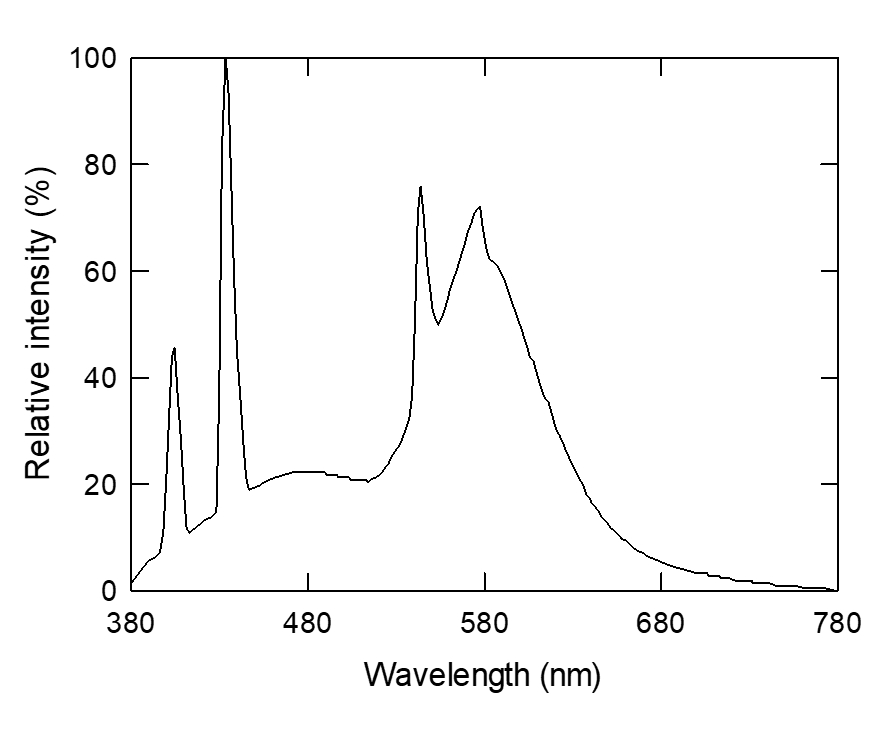


**Figure S14. Spectral energy distribution of the lighting source for colour measurements.**
